# Prevalence of hypoxaemia in children with pneumonia in low-income and middle-income countries: a systematic review and meta-analysis

**DOI:** 10.1016/S2214-109X(21)00586-6

**Published:** 2022-02-15

**Authors:** Ahmed Ehsanur Rahman, Aniqa Tasnim Hossain, Harish Nair, Mohammod Jobayer Chisti, David Dockrell, Shams El Arifeen, Harry Campbell

**Affiliations:** a The University of Edinburgh, Edinburgh, UK; b International Centre for Diarrhoeal Disease Research, Bangladesh, Dhaka, Bangladesh

## Abstract

**Background:**

Pneumonia accounts for around 15% of all deaths of children younger than 5 years globally. Most happen in resource-constrained settings and are potentially preventable. Hypoxaemia is one of the strongest predictors of these deaths. We present an updated estimate of hypoxaemia prevalence among children with pneumonia in low-income and middle-income countries.

**Methods:**

We conducted a systematic review using the following key concepts “children under five years of age” AND “pneumonia” AND “hypoxaemia” AND “low- and middle-income countries” by searching in 11 bibliographic databases and citation indices. We included all articles published between Nov 1, 2008, and Oct 8, 2021, based on observational studies and control arms of randomised and non-randomised controlled trials. We excluded protocol papers, articles reporting hypoxaemia prevalence based on less than 100 pneumonia cases, and articles published before 2008 from the review. Quality appraisal was done with the Joanna Briggs Institute tools. We reported pooled prevalence of hypoxaemia (SpO_2_ <90%) by classification of clinical severity and by clinical settings by use of the random-effects meta-analysis models. We combined our estimate of the pooled prevalence of pneumonia with a previously published estimate of the number of children admitted to hospital due to pneumonia annually to calculate the total annual number of children admitted to hospital with hypoxaemic pneumonia.

**Findings:**

We identified 2825 unique records from the databases, of which 57 studies met the eligibility criteria: 26 from Africa, 23 from Asia, five from South America, and four from multiple continents. The prevalence of hypoxaemia was 31% (95% CI 26–36; 101 775 children) among all children with WHO-classified pneumonia, 41% (33–49; 30 483 children) among those with very severe or severe pneumonia, and 8% (3–16; 2395 children) among those with non-severe pneumonia. The prevalence was much higher in studies conducted in emergency and inpatient settings than in studies conducted in outpatient settings. In 2019, we estimated that over 7 million children (95% CI 5–8 million) were admitted to hospital with hypoxaemic pneumonia. The studies included in this systematic review had high τ^2^ (ie, 0·17), indicating a high level of heterogeneity between studies, and a high *I*^2^ value (ie, 99·6%), indicating that the heterogeneity was not due to chance. This study is registered with PROSPERO, CRD42019126207.

**Interpretation:**

The high prevalence of hypoxaemia among children with severe pneumonia, particularly among children who have been admitted to hospital, emphasises the importance of overall oxygen security within the health systems of low-income and middle-income countries, particularly in the context of the COVID-19 pandemic. Even among children with non-severe pneumonia that is managed in outpatient and community settings, the high prevalence emphasises the importance of rapid identification of hypoxaemia at the first point of contact and referral for appropriate oxygen therapy.

**Funding:**

UK National Institute for Health Research (Global Health Research Unit on Respiratory Health [RESPIRE]; 16/136/109).

## Introduction

Pneumonia accounts for approximately 15% of all deaths in children younger than 5 years, and most of these deaths occur in low-income and middle-income countries (LMICs).^1^, ^2^, ^3^ Cognisant of the high burden of mortality, WHO declared pneumonia a “forgotten killer of children”.^4^ Without effectively preventing and treating pneumonia, countries with a high burden of pneumonia will not reach the ambitious UN Sustainable Development Goal target of reducing the mortality rate of children younger than 5 years to less than or equal to 25 per 1000 livebirths by 2030.^5^

Hypoxaemia, defined as low oxygen saturation in arterial blood (ie, SpO_2_ <90%), is common among children with pneumonia and other acute lower respiratory infections, and is one of their strongest predictors of mortality.^3^, ^6^, ^7^, ^8^, ^9^, ^10^ WHO recommends measuring SpO_2_ in routine practice, even in settings with constrained resources, as it can substantially improve the accuracy of classification of pneumonia and other acute lower respiratory infections by clinical severity.^11^, ^12^

**Research in context**

**Evidence before this study**

A review paper by Lozano and colleagues in 2001 reported a hypoxaemia prevalence of 31% among children presenting with acute lower respiratory infection in emergency settings, 43% among children with clinical pneumonia, and 47% among children who were admitted to hospital with pneumonia. The previous global estimate of hypoxaemia prevalence is based on a systematic review conducted by Subhi and colleagues in 2009, which reported a median hypoxaemia prevalence of 13% (IQR 9·3–37·5) among young children with WHO-classified pneumonia requiring admission to hospital. A systematic review conducted by Lazzerini and colleagues in 2015 reported that hypoxaemia was associated with five-times higher odds of death from acute lower respiratory infections. This updated systematic review was done following WHO's revised clinical pneumonia classification for children in 2014, the introduction of pneumococcal and *Haemophilus influenzae* vaccines into the routine childhood immunisation programmes of many low-income and middle-income countries, and concerns about oxygen security in the context of the COVID-19 pandemic. We searched MEDLINE, Embase, Global Health via Ovid, and Cochrane Central Register of Controlled Trials (CENTRAL) with the following search terms: “children under five years of age” AND “pneumonia” AND “hypoxaemia” AND “low- and middle-income countries” AND “systematic review/meta-analysis” for studies published in English between Nov 1, 2008, and Oct 8, 2021.

**Added value of this study**

This Article reports an updated estimate of hypoxaemia prevalence among children with WHO-classified pneumonia in low-income and middle-income countries on the basis of searches of 11 bibliographic databases and citation indices. We identified 57 studies and presented both the pooled and median prevalence estimates on the basis of more than 100 000 observations, whereas the previous estimate in 2009 was based on around 18 000 observations. The estimates were further disaggregated by clinical severity classifications, clinical settings, hospitalisation status, and altitude levels.

**Implications of all the available evidence**

This study presents an estimated number of children who are admitted to hospital due to pneumonia with hypoxaemia annually. The high prevalence of hypoxaemia among children with WHO-classified severe pneumonia, reported in this systematic review, emphasises the importance of overall oxygen security within health systems, particularly in the context of COVID-19. The high prevalence of hypoxaemia among children with non-severe pneumonia accentuates the need for novel diagnostics, such as pulse oximetry, at the first point of contact and urgent referral for prompt management of children with hypoxaemia.

Despite the high mortality risk, little is known about the prevalence of hypoxaemia among children with pneumonia in LMICs.^13^ The most recent global estimate is based on Subhi and colleagues’ systematic review published in 2009,^8^ which reported a median hypoxaemia prevalence of 13·3% (IQR 9·3–37·5) among young children with WHO-classified pneumonia requiring admission to hospital. The prevalence varied widely across countries, age groups, and classifications of clinical severity.^8^ Subhi and colleagues’ systematic review predates the revised WHO pneumonia classifications of 2014, which included recommendations for assessing SpO_2_ in routine practice.^11^, ^12^, ^14^ It is an appropriate time to update the systematic review and meta-analysis to include children classified with WHO's 2014 pneumonia classification.

We aimed to report an updated prevalence of hypoxaemia among children younger than 5 years with WHO-classified pneumonia in LMICs, stratified by classification of clinical severity, clinical settings, and altitude levels, on the basis of articles published between 2008 and 2021.

## Methods

### Search strategy and selection criteria

In this systematic review and meta-analysis, we conducted an initial scoping review to help inform our search strategy. The search terms were “children under five years of age” AND “pneumonia” AND “hypoxaemia” AND “low- and middle- income countries”. The detailed search strategy is presented in the (appendix pp 1–50). The list of LMICs (appendix pp 50–54) was obtained from the UN Statistics Division.^15^ We searched MEDLINE (via OVID), Embase (via OVID), Global Health (via OVID), Cochrane Central Register of Controlled Trials, ClinicalTrials.gov, Scopus, CINHAL, PubMed, Global Index Medicus, IndMED, Global Index Medicus by WHO, including African Index Medicus, the literature of Latin America and the Caribbean (AMRO/Pan American Health Organization), the Index Medicus for the Eastern Mediterranean Region (IMEMR/ EMRO), the Index Medicus for South-East Asia Region (IMSEAR/ SEARO), Western Pacific Region Index Medicus (WPRIM/ WPRO), Web of Science, and Social Sciences Citation Index (SCI index) for all studies published between Nov 1, 2008, and Oct 8, 2021, without any language restrictions.

We included studies with children younger than 5 years with WHO-classified pneumonia (ie, assessed with the Integrated Management of Childhood Illness or WHO's Pocket Book).^11^, ^12^ Hypoxaemia assessed by any health-service provider (eg, doctors, nurses, paramedics, and community health workers) through pulse oximetry and reported as a prevalence estimate was considered as the outcome.^16^, ^17^ We did not include any grey literature or any unpublished studies in our systematic review. Both observational studies and control groups of randomised and non-randomised controlled trials were included. We excluded articles reporting hypoxaemia prevalence based on less than 100 patients with pneumonia to minimise the error margin and articles where pulse oximetry was used to identify a particular disease or condition.^8^ Finally, we excluded articles published before 2008 (ie, the upper limit of the previous review by Subhi and colleagues) during the full-text review process.^8^ Here, we present our main findings based on the WHO-classified pneumonia cases. We also assessed the prevalence of hypoxaemia among non-WHO pneumonia cases to contextualise our results, and we present these results in the appendix.

The review team consisted of topic experts in pneumonia and hypoxaemia (AER, HC, HN, and DD), an epidemiologist (SEA), a statistician (ATH), and a librarian and systematic review expert (Marshall Dozier). After removing duplicates, two reviewers (AER and Sabrina Jabeen) independently screened the articles with a structured checklist (appendix p 56). Then two reviewers (AER and Shema Mhajabin) independently reviewed the full-text articles and abstracted data using another structured checklist (appendix pp 56–58). The details of reviewed articles are provided in the appendix (pp 59–62). The inclusion criteria were the same for the systematic review and the meta-analysis.

We adapted the tool developed by the Joanna Briggs Institute for quality assessment of studies included for data abstraction, which was undertaken by two reviewers independently (appendix pp 63–65).^18^ In case of disagreement, a third reviewer was consulted.^19^

### Data analysis

We abstracted data by use of a structured checklist (appendix pp 55–58) and two reviewers independently entered data using a template designed in SPSS (version 19). Duplicate articles were primarily removed with EndNote (version 20), and afterwards duplicate data from the selected articles were removed through manual checks. We used Stata version 14 and R package (version 4.0.5) for statistical analysis. We calculated the pooled prevalence of hypoxaemia with 95% CIs using the random-effects models.^20^, ^21^ We also reported medians with IQR. The pooled estimates were stratified by classification of clinical severity (ie, “very severe pneumonia” or “severe pneumonia” was stratified as “very severe or severe pneumonia” and “pneumonia” was stratified as “non-severe pneumonia” and “unclassified pneumonia” where the study used WHO classification but did not differentiate between severe and non-severe pneumonia), clinical settings (ie, indoor, emergency, or outdoor), and altitude levels (ie, high altitude if at least 2400 m above sea level).^11^, ^12^ We could not report the hypoxaemia prevalence by ethnicity or sex as we could not extract such disaggregated data from the selected articles. Publication bias was assessed with funnel plots by use of R package's funnel command.^22^, ^23^ We reported the τ^2^ value as a measure of heterogeneity across studies and *I*^2^ value to explain the proportion of heterogeneity that was not due to chance (appendix p 66).

We also estimated the total number of children admitted to hospital with hypoxaemic pneumonia annually by use of McAllister and colleagues’ estimated number of children admitted to hospitals due to pneumonia annually and the pooled hypoxaemia prevalence among children admitted to hospital in LMICs reported in this systematic review.^24^

We followed the Preferred Reporting Items for Systematic Reviews and Meta-Analyses guidelines for reporting.^25^ This protocol is registered at PROSPERO, CRD42019126207.^19^

### Role of the funding source

The funder of the study had no role in study design, data collection, data analysis, data interpretation, or writing of the report.

## Results

**Figure 1:**
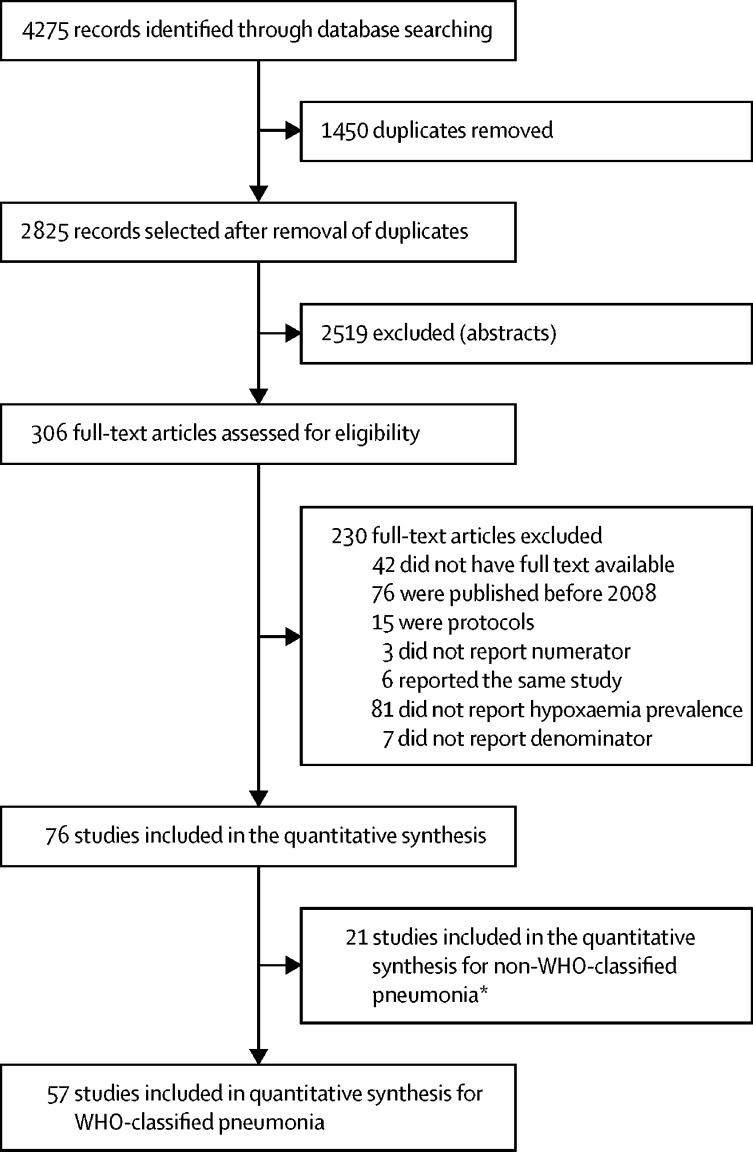
Flow diagram of the study selection process *Two studies reported both WHO-classified and non-WHO-classified pneumonia.

**Figure 2:**
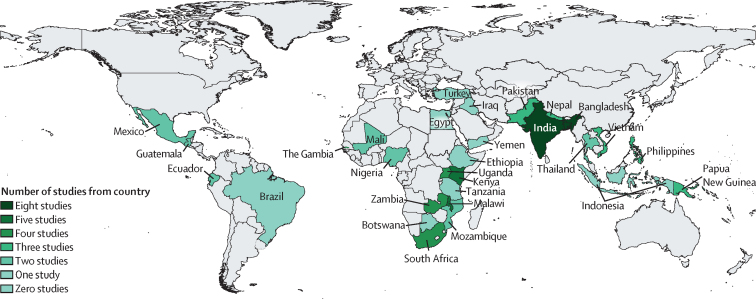
Distribution of countries and the number of studies reporting hypoxaemia prevalence among children with WHO-classified pneumonia 57 articles reporting hypoxaemia prevalence among children with WHO-classified pneumonia by country. Among these studies, 26 were from Africa, 23 from Asia, five from South America, and four from multiple continents.

We identified 4275 records, of which 1450 were duplicates (appendix pp 66–67; figure 1). After title and abstract screening, 306 articles were selected for full-text review. 230 articles were excluded because they did not meet the eligibility criteria. Data were abstracted from 76 articles (two articles reported hypoxaemia among children with WHO-classified pneumonia and non-WHO-classified pneumonia), of which 57 studies reported hypoxaemia prevalence among children with WHO-classified pneumonia (with 61 prevalence estimates) and 101 775 children were involved (appendix p 77); the remaining articles (ie, 21 articles) reported hypoxaemia prevalence among children with non-WHO-classified pneumonia or acute lower respiratory infection. Among the 57 studies reporting hypoxaemia prevalence among children with WHO-classified pneumonia, 26 studies were from Africa, 23 from Asia, five from South America, and four from multiple continents (figure 2).

Five of 57 studies were conducted at high-altitude sites (appendix pp 67–68). 30 studies were done in tertiary-level referral facilities, 12 studies were in primary-level or secondary-level referral facilities, six studies were in health centres, and six studies were in mixed settings. Facility information could not be extracted from three articles. Eight studies measured SpO_2_ in the emergency setting, 32 studies in the in-patient setting, 12 studies in the outpatient setting, and five studies in mixed settings. 56 studies reported hypoxaemia prevalence (40 in hospitalised children, 12 in non-hospitalised children, and four in a mixed population of hospitalised and non-hospitalised children). We could not ascertain the patient status from one study. 28 studies reported hypoxaemia prevalence among children with WHO-classified very severe or severe pneumonia, and five studies reported prevalence among children with non-severe pneumonia. Classification of clinical severity was not explicitly mentioned in 28 studies, which are presented as unclassified pneumonia. 29 studies mentioned the type (ie, table-top, handheld, fingertip), manufacturer, and models of the pulse oximeters used, and 13 studies explicitly mentioned use of a paediatric probe. 38 studies used the WHO-recommended SpO_2_ cutoff of less than 90%, 12 studies used less than 92%, two studies used less than 93%, one study used less than 94%, two studies used less than 95%, one study used less than 87%, and one study used 90–93%.

**Figure 3:**
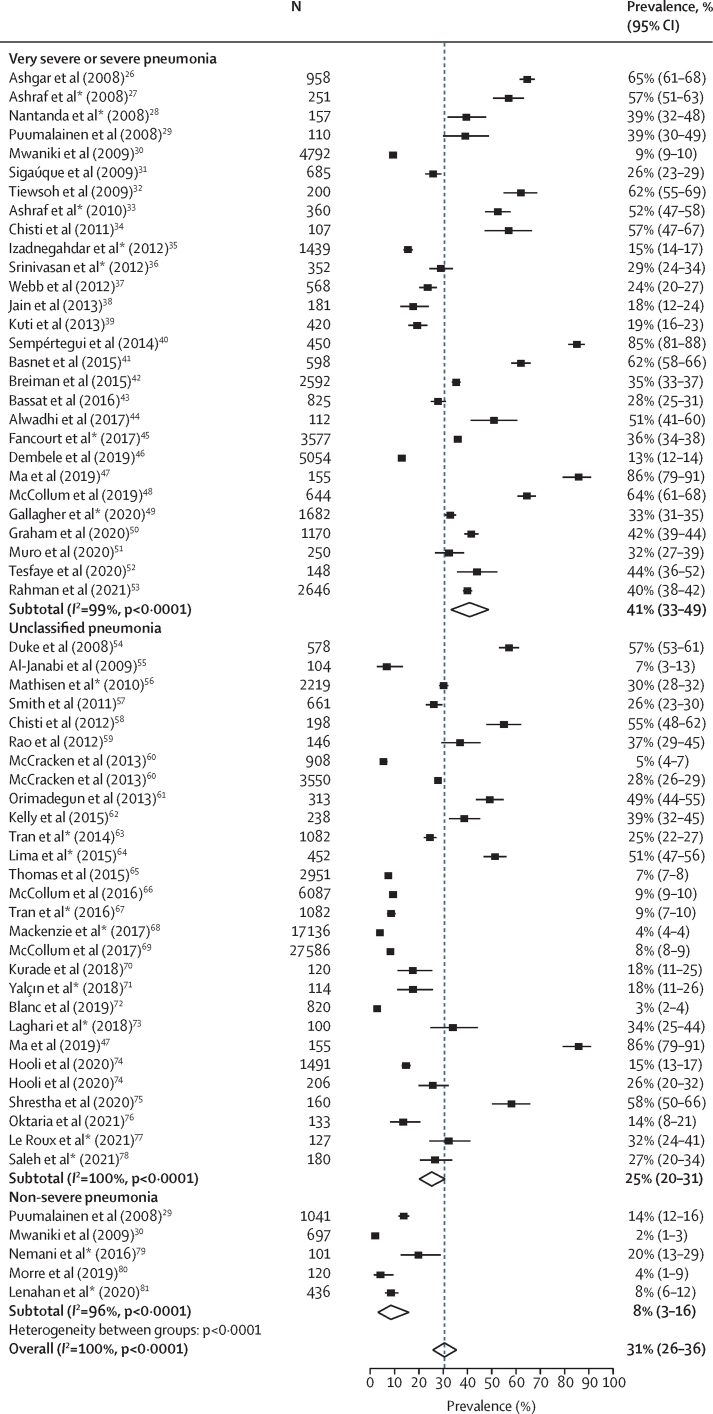
Hypoxaemia prevalence among children with WHO-classified pneumonia by clinical severity Dashed line indicates overall prevalence. *Studies where SpO_2_ cutoff is less than 90%.

Figure 3 presents the hypoxaemia prevalence among WHO-classified pneumonia by clinical severity. The pooled overall prevalence of hypoxaemia was 31% (95% CI 26–36) among 101 775 children with WHO-classified pneumonia**.** The median overall prevalence was 29% (IQR 15–49). The prevalence was 41% (95% CI 33–49) among 30 483 children with very severe or severe pneumonia, 8% (3–16) among 2395 children with non-severe pneumonia, and 25% (20–31) among 68 897 children with unclassified pneumonia. The studies included in this review had high τ^2^ values (τ^2^=0·18 for very severe or severe pneumonia, τ^2^=0·06 for non-severe pneumonia, and τ^2^=0·10 for unclassified pneumonia), indicating a high level of heterogeneity between studies, and high *I*^2^ values (99·5% for very severe or severe pneumonia, 96·3% for non-severe pneumonia, and 99·5% for unclassified pneumonia), indicating that the heterogeneity was not due to chance.

**Figure 4:**
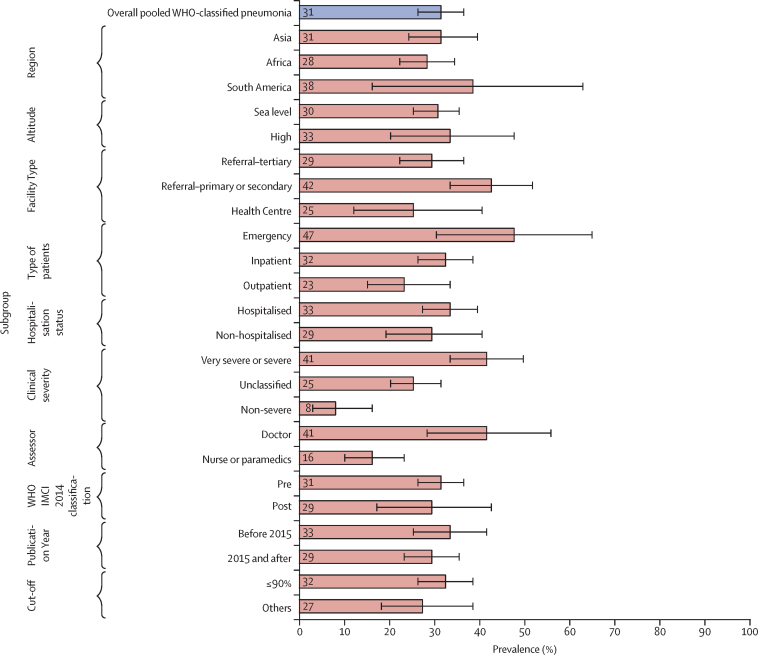
Hypoxaemia prevalence among children with WHO-classified pneumonia and comparison between random-effects pooled estimate across different subgroups IMCI=Integrated Management of Childhood Illness.

Figure 4 shows wide variations in hypoxaemia prevalence among children with WHO-classified pneumonia across different categories. Regarding classification of clinical severity, hypoxaemia prevalence was highest among children classified with severe pneumonia. Although the confidence intervals are overlapping, the point prevalence was higher among studies conducted in emergency (47%, 95% CI 30–64) and inpatient (32%, 26–38) settings than among those conducted in outpatient settings (23%, 15–33). The estimates were not substantially different between regions (Asia 31%, 24–39; Africa 28%, 22–34) or by hospitalisation status of children (33%, 27–39 in children admitted to hospital; 29%, 19–40 in children not admitted to hospital). The overall estimate did not vary between classifications before 2014 and classifications after 2014, but there were some differences in unclassified pneumonia estimates (appendix p 69). Median (IQR) prevalence across different subgroups are found in the appendix (pp 69–70).

**Figure 5:**
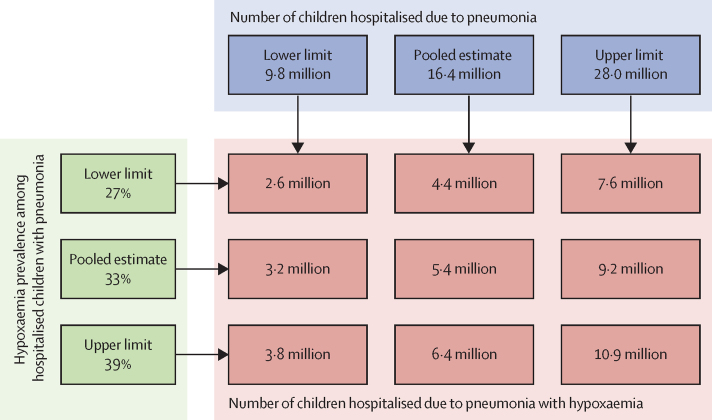
Global estimated number of children admitted to hospital due to pneumonia with hypoxaemia annually

Figure 5 presents a scenario-based projection of the estimated number of children admitted to hospital due to pneumonia with hypoxaemia annually. On the basis of the estimated number (ie, 16·4 million) of children admitted to hospital due to pneumonia annually reported by McAllister and colleagues, and hypoxaemia prevalence (ie, 33%) among children admitted to hospital with pneumonia reported in our systematic review, we estimate that approximately 5·4 million children were admitted to hospital due to pneumonia with hypoxaemia in 2015. If we consider the lower limits and upper limits of hypoxaemia prevalence (27–39%) based on our study, the estimated number was between 4·4 million and 6·4 million. Assuming an average annual growth rate of 7% over the period of 2000–15 and continued over the period 2015–19, hospitalisations due to hypoxaemic pneumonia would have been close to 7·2 million in 2019.^24^

The hypoxaemia prevalence was 36% (95% CI 26–46) among children with non-WHO classified clinical pneumonia (13 studies), 19% (11–29) among children with radiological pneumonia (six studies), and 33% (29–37) among organism-specific pneumonia (ie, respiratory syncytial virus; two studies; appendix p 78).

During the screening and full-text review, we included only observational studies and the control group of randomised and non-randomised controlled trials with adequate sampling size (≥100 participants). During the quality assessment, we found that the description regarding the sampling strategy was inadequate in two articles. Funnel plots assessing the publication bias of the selected articles can be found in the appendix (pp 78–80). Overall, the τ^2^ value was 0·17, indicating a high level of heterogeneity between studies, and *I*^2^ value was 99·6%, indicating that the heterogeneity was not due to chance.

## Discussion

This systematic review reports a high prevalence of hypoxaemia among children with WHO-classified pneumonia, which is one of the strongest predictors of mortality due to pneumonia.^3^, ^6^, ^7^, ^8^, ^9^, ^10^ Ensuring rapid identification of hypoxaemia through routine assessment of SpO_2_ and immediate hospital admission for oxygen therapy with supportive care can substantially reduce pneumonia-related childhood mortality.^54^, ^82^ In this Article, we addressed a notable evidence gap by presenting the burden of hypoxaemia among children with pneumonia in LMICs. The pooled estimates included 57 studies with more than 100 000 children with pneumonia and reasonably represented different regions, facility types, patient types, hospitalisation status, and clinical severity based on WHO classifications. The overall prevalence of hypoxaemia was high, particularly among patients with clinically severe pneumonia or those who were admitted to hospitals. We also found a moderately high prevalence among children with non-severe pneumonia.

The overall prevalence of hypoxaemia reported here is higher than that reported by Subhi and colleagues^8^ in 2009 but similar to that reported by Lozano in 2001.^83^ In our systematic review, the overall prevalence was 31% (95% CI 26–36), and the prevalence was 33% (27–39) among hospitalised children and 47% (30–64) among children presenting in the emergency department. Lozano reported a prevalence of 31% among children presenting with acute lower respiratory infection in emergency settings, 43% among children with clinical pneumonia, and 47% among hospitalised children with pneumonia. By contrast, Subhi and colleagues reported a much lower prevalence of 13% among young children who were admitted to hospital with pneumonia. The introduction of pneumococcal and *Haemophilus influenzae* vaccines in the routine childhood immunisation programmes in many LMICs in the past decade is expected to reduce the community burden of pneumonia and severe pneumonia, including hypoxaemic pneumonia. However, during the same period, the increasing trend towards improving the socioeconomic conditions, availability and access to health care, and provision and practice of SpO_2_ assessment and oxygen security in health facilities might have led to better care-seeking practices and a higher level of detection of hypoxaemia in lower-level health centres and in referral facilities than before.^84^, ^85^, ^86^ Another reason could be the proportional contribution of hospitalised children in our review. Of the 57 studies, eight reported measuring SpO_2_ in emergency settings, whereas 32 reported measuring in inpatient settings. It is expected that these cases will be more severe and have a higher prevalence of hypoxaemia than those in outpatient settings.^87^ Moreover, Subhi and colleagues included children younger than 12 years, whereas we included only children younger than 5 years. This difference in age might be one of the reasons explaining the difference in prevalence, as the prevalence of hypoxaemia is lower among older children than younger children.^53^ In our systematic review, the prevalence of hypoxaemia varied only slightly between the Asian and African regions, with overlapping confidence intervals, which is different from the large difference reported by Subhi and colleagues.^8^ One possible explanation is the larger sample size of our review than in Subhi and colleagues’ review, which has contributed to generating more precise estimates with overlapping confidence intervals. The other explanation is the inclusion of more children with very severe or severe pneumonia among Asian studies and a differing spectrum of causes of pneumonia across different countries of Asia and Africa.^88^, ^89^ Moreover, Subhi and colleagues reported a hypoxaemia prevalence of less than 10% among children with pneumonia in the African region,^8^ which was much lower than the prevalence estimates (ie, 28–57%) reported in most studies from the same region.^43^, ^61^, ^86^, ^90^, ^91^, ^92^, ^93^ Lastly, hypoxaemia can be a transient phenomenon, and SpO_2_ status can change after initial stabilisation.^61^ The studies included in our review have exclusively reported hypoxaemia on the basis of the initial contact with health systems. Since few studies have follow-up SpO_2_ status, the pooled prevalence could be an overestimation of the actual burden.

We noted the hypoxaemia prevalence to be much higher among children who were admitted to hospital and studies conducted in inpatient and emergency settings than those that were conducted in outpatient and community settings. According to WHO, children presenting with clinical features of severe pneumonia require admission to hospital with supportive care.^94^ Therefore, it is highly likely that the studies conducted among children who were admitted to hospital and in emergency and inpatient settings predominantly enrolled children with clinically severe pneumonia, for whom the prevalence of hypoxaemia is expected to be higher than in those managed as outpatients. The prevalence was also higher in emergency settings than in inpatient settings, possibly because hypoxaemia was measured after initial stabilisation in studies reporting the prevalence among children who were inpatients.

We estimate that the total number of children admitted to hospital due to pneumonia with hypoxaemia was 5·5 million in 2015.^24^ Given the increase in the rate of hospitalisations during 2000–15, this estimate might be an underestimation of the current burden. If this rate of increase continued to apply over 2015–19, then the estimated number of hypoxaemic pneumonia-related hospitalisations in young children would be close to 7·2 million in 2019.^24^ This estimate does not include patients with hypoxaemic severe pneumonia who could not reach hospitals for appropriate care or died in the community or those who were classified with non-severe pneumonia.

In our systematic review, the prevalence of hypoxaemia among children with non-severe pneumonia was 8%, implying that some children with non-severe pneumonia (with no other danger signs) actually would require oxygen therapy with supportive care.^16^ This implication could potentially explain the findings of Fox and colleagues’ systematic review,^95^ which reported that children with chest in-drawing pneumonia who were treated with only oral antibiotics, on the basis of the 2014 WHO recommendations, had a high rate of non-response to treatment.^94^, ^96^, ^97^, ^98^, ^99^

It is important to consider the strengths and limitations of our systematic review and compare our search strategy and execution with the previous reviews. We conducted a scoping review to fine-tune the search strategy and keywords. In addition to international databases, we expanded our search to multiple national databases representing south Asia, the Middle East, Africa, and Latin America, which added robustness to our review with adequate sensitivity. Although our systematic review and meta-analysis had similar objectives to Subhi and colleagues^8^ and Lozano and colleagues,^83^ there were some differences in execution. Subhi and colleagues used around 18 000 observations of children younger than 12 years, which included 12 published papers (up to November, 2008) from three international databases and 12 unpublished studies.^8^ Lozano and colleagues included 17 published articles, involving around 4000 children from one database and covering January, 1966, to August, 1999.^83^ Our review was more comprehensive than these two reviews and included 57 published studies from 11 international and national bibliographic databases, covering the period of January, 2008, to October, 2021, and involved over 100 000 children younger than 5 years with pneumonia. We did not include unpublished studies and Chinese databases, which could have influenced our estimates.

WHO changed its pneumonia classification in 2014, and our review included studies that were published from 2008 and onwards.^94^ In many instances, we could not determine the version of WHO pneumonia classifications that different studies had adopted when reporting hypoxaemia prevalence. Although we did not have enough information regarding the presenting signs and symptoms to map the clinical severity classification on the basis of the 2014 WHO guidelines, we reported the pooled estimates by different clinical severity classifications separately. Additionally, different studies used different definitions of hypoxaemia and different SpO_2_ cutoffs. However, these differences did not affect the overall estimate, since the difference was not notable between the studies that used the WHO recommended cutoff of less than 90% and those that used 90–95%. We struggled to extract prevalence estimates by different age bands, type of providers who measured SpO_2_ (eg, doctors, nurses, paramedics, community health workers, etc), type of training or orientation received, and type of pulse oximeters and probes used for assessments. We could not report the validity and reliability of assessments by different providers with different types of pulse oximetry in various clinical settings. There could be some selection bias in the studies, but inadequate information existed to assess bias due to inconsistency when reporting hypoxaemia prevalence by various categories across different studies. We recommend a minimum set of standards to report hypoxaemia prevalence in future studies. Lastly, we acknowledge that we reported hypoxaemia prevalence on the basis of SpO_2_ status measured by pulse oximeters, whereas the gold standard is arterial blood-gas analysis. Although several studies have reported a strong correlation between pulse oximetry and blood-gas analysis measures in controlled clinical settings, pulse oximeter measures can be affected by various clinical, mechanical, and device-related factors.^100^, ^101^, ^102^, ^103^ Unfortunately, we could not adjust our estimates for these variables.

Although we conducted a rigorous quality assessment by use of the checklist developed by the Joanna Briggs Institute, which is specially designed for studies reporting prevalence or cumulative incidence, we acknowledge the potential effect of publication bias.^18^ Although we reported some extreme values in the funnel plot (appendix pp 78–80), the individual prevalence estimates were widely dispersed in both directions (with no apparent skewing) from the pooled estimate. This dispersion could be explained by the large heterogeneity among these studies, indicated by high τ^2^ and *I*^2^ values.^104^ Therefore, we used the random-effects model to generate the pooled prevalence estimate. We also reported median (IQR) for all reporting categories to maintain comparability with previous estimates.^8^

In addition to the high prevalence of hypoxaemia among children with WHO-classified pneumonia, we also reported somewhat similar prevalence among children with non-WHO-classified pneumonia. Hypoxaemia is also common among children admitted to hospital with asthma, meningitis or encephalitis, malnutrition, acute febrile encephalopathy, sepsis, or malaria and neonates with neonatal encephalopathy, prematurity, or sepsis.^86^, ^105^ Hence, hypoxaemia can be considered as a stand-alone danger sign among young children, irrespective of clinical classification. The use of pulse oximetry with appropriate oxygen therapy can reduce pneumonia-related mortality rates and length of hospital stay.^106^ Hence, WHO recommends routine assessment of SpO_2_, rapid identification of hypoxaemia, and immediate admission to hospital for oxygen therapy with other supportive care to avert these hypoxaemia-related deaths.^94^, ^107^, ^108^ However, there are gaps between policy and practices. The availability of pulse oximeters, the provision of oxygen therapy during referral, transportation, and inpatient care, and the overall oxygen security are still inadequate in most LMICs.^109^, ^110^, ^111^, ^112^ Therefore, urgent attention should be given to improving the access, provision, and quality of care, including SpO_2_ assessment and oxygen therapy in resource-poor settings.^82^ Although, pulse oximeters are reasonably valid, affordable, easy-to-use devices that identify hypoxaemia immediately, there are several health systems barriers and operational challenges associated with introducing pulse oximeters in LMICs.^113^, ^114^, ^115^ The characteristics of public health systems and the health-service providers’ skills and capacity in each country are different and might present a unique set of context-specific challenges. Further research studies on implementation are needed to understand the feasibility aspects, focusing on providers’ capacity to measure SpO_2_, choice of device, and time required for SpO_2_ assessment. Emphasis should be given to training staff, building a culture of hypoxaemia assessment, and promoting judicial use of oxygen.^16^ National programmes should give increased attention and funding support to addressing these challenges, particularly in the context of the COVID-19 pandemic, as this support might improve the overall readiness and performance of health systems regarding emergency triage and management.^116^, ^117^

In conclusion, the high prevalence of hypoxaemia among children with WHO-classified severe pneumonia, particularly among children who were admitted to hospital, emphasises the importance of oxygen security within LMIC health systems. Without the provision of rapid identification and prompt management of hypoxaemia, it will not be possible to substantially reduce pneumonia-related deaths. On the basis of the findings of this systematic review and the context of the COVID-19 pandemic, we strongly recommend introducing pulse oximetry at the first point of contact after feasibility assessment and health systems integration of pulse oximetry, especially in resource-constrained settings. We also emphasise the need for regular monitoring SpO_2_ levels in hospital settings to use oxygen correctly and efficiently.

## Data sharing

The study protocol, with all relevant tools, is publicly available at PROSPERO, CRD42019126207. The data used for this meta-analysis will be made available on request to the corresponding author.

## Declaration of interests

HN reports grants from Innovative Medicines Initiative, Pfizer, and WHO and honoraria from Sanofi, Janssen, Novavax, and ReViral, outside the submitted work. HC reports consulting fees from the Bill & Melinda Gates Foundation via his institution, and funding to attend meetings from the National Institute for Health Research via his institution, outside the submitted work.
