# Supplementary material for: Prevalence of hypoxaemia in children with pneumonia in low-income and middle-income countries: a systematic review and meta-analysis
Source: Lancet Glob Health. 2022 Feb 15;10(3):e348–59. doi: 10.1016/S2214-109X(21)00586-6 (PMC8864303; doi:10.1016/S2214-109X(21)00586-6)
Supplement: Supplementary appendix [file mmc1.pdf]

THE LANCET
Global Health

Supplementary appendix

This appendix formed part of the original submission and has been peer reviewed. We post it as supplied by the authors.

Supplement to: Rahman AE, Hossain AT, Nair H, et al. Prevalence of hypoxaemia in children with pneumonia in low-income and middle-income countries: a systematic review and meta-analysis. *Lancet Glob Health* 2022; **10:** e348–59.

Supplementary table 1: Detailed search language with keywords and their synonyms

	1	BO	2	BO	3	BO	4
	**Outcome**		**Population**		**Population**		**Context**
**Concepts**	**hypoxaemia**	**AND**	**pneumonia**	**AND**	**children under 5 years of age**	**AND**	**low- and middle-income countries**
**Keywords**	hypoxaemia/<br>hypoxaemia/<br>anoxia/<br>hypoxia/<br>oximetry/<br>oximeter/<br>oximetre/<br>oxymetry/<br>oxymeter/<br>oxymetre/<br>blood oxygen saturation/<br>arterial oxygen saturation/<br>blood gas monitoring/<br>blood gas analysis/<br>analysis blood gas/<br>SpO2/<br>SPO2		pneumonia/<br>severe pneumonia/<br>severe disease/<br>very severe disease/<br>bronchiolitis/<br>acute respiratory infection/<br>ARI/<br>acute respiratory infections/<br>acute respiratory tract infection/<br>ARTI/<br>acute respiratory tract infections/<br>respiratory illness/<br>respiratory illnesses/		child*/<br>infant*/<br>pediatric*/<br>paediatric*/		developing countr*/<br>low-and middle income countr*<br>LMIC*/<br>low income countr*/<br>middle income countr* /<br>lower middle income countr*/<br>upper middle income countr*/<br>'name of the country(appendix-01)'
	**BO:** Boolean Operator						

Search Summary-1

Name of the database

MEDLINE (via Ovid)

Date of search

08 October 2021

Timeline

Not applicable

Search Language

Line #	Theme	Search Language	Output #
1	Hypoxaemia	hypoxemia.mp. or exp Hypoxia/	91820
2		exp Hypoxia/ or hypoxaemia.mp.	84800
3		anoxia.mp. or exp Hypoxia/	87644
4		hypoxia.mp. or exp Hypoxia/	155924
5		oximetry.mp. or exp Oximetry/	19678
6		exp Oximetry/ or oximeter.mp.	16924
7		exp Oximetry/ or oximetre.mp.	15892
8		exp Oximetry/ or oxymetry.mp.	16149
9		exp Oximetry/ or oxymeter.mp.	15980
10		exp Oximetry/ or oxymeter.mp.	15980
11		blood oxygen saturation.mp.	1426
12		arterial oxygen saturation.mp.	4055
13		exp Blood Gas Monitoring, Transcutaneous/ or blood gas monitoring.mp. or exp Blood Gas Analysis/	37305
14		blood gas analysis.mp. or exp Blood Gas Analysis/	40194
15		analysis blood gas.mp. or exp Blood Gas Analysis/	37226
16		SpO2.mp.	4886
17		SPO2.mp.	4886
18		1 or 2 or 3 or 4 or 5 or 6 or 7 or 8 or 9 or 10 or 11 or 12 or 13 or 14 or 15 or 16 or 17	211488
19	Pneumonia	exp Pneumonia, Bacterial/ or exp Pneumonia/ or exp Pneumonia, Viral/ or pneumonia.mp.	258674
20		exp Pneumonia, Bacterial/ or exp Pneumonia/ or severe pneumonia.mp. or exp Pneumonia, Viral/	205112
21		severe disease.mp.	18122

22		very severe disease.mp.	199
23		exp Bronchiolitis Obliterans/ or bronchiolitis.mp. or exp Bronchiolitis/ or exp Bronchiolitis, Viral/	13139
24		exp Respiratory Tract Infections/ or acute respiratory infection.mp. or exp Respiratory Syncytial Virus Infections/ ()	482032
25		exp Respiratory Tract Infections/ or ARI.mp. ()	478927
26		exp Respiratory Tract Infections/ or acute respiratory infections.mp. or exp Respiratory Syncytial Virus Infections/	482231
27		exp Respiratory Tract Infections/ or acute respiratory tract infection.mp.	476982
28		exp Respiratory Tract Infections/ or ARTI.mp.	478414
29		exp Respiratory Tract Infections/ or acute respiratory tract infections.mp.	477025
30		exp Respiratory Tract Infections/ or respiratory illness.mp. or exp Respiratory Tract Diseases/	1511022
31		exp Respiratory Tract Infections/ or respiratory illnesses.mp. or exp Respiratory Tract Diseases/	1510299
32		19 or 20 or 21 or 22 or 23 or 24 or 25 or 26 or 27 or 28 or 29 or 30 or 31	1565662
33	Children under-5 years of age	Child.mp. or exp Child Health Services/ or exp Child Care/ or exp Child, Hospitalized/ or exp Child/ or exp Child Health/	2175248
34		Children.mp. or exp Child/	2200753
35		Infant.mp. or exp Infant Care/ or exp Infant Health/ or exp Infant/	1230190
36		Infants.mp. or exp Infant/	1226015
37		Pediatric.mp. or exp Pediatrics/	320962
38		Pediatrics.mp. or exp Pediatrics/	82943
39		exp Child/ or exp Child, Preschool/ or Paediatric.mp.	2022745
40		exp Child/ or exp Child, Preschool/ or Paediatrics.mp.	2012017
41		33 or 34 or 35 or 36 or 37 or 38 or 39 or 40	2914291
42		18 and 32 and 41	7758
43	Low and Middle Income country	exp Developing Countries/ or developing countr*.mp.	122287
44		exp Developing Countries/ or low-and middle income country.mp.	78317

45		exp Developing Countries/ or low-and middle income countries.mp.	86721
46		exp Developing Countries/ or LMIC*.mp.	80240
47		exp Developing Countries/ or low income country.mp.	78344
48		exp Developing Countries/ or middle income country.mp.	79281
49		exp Developing Countries/ or low income countries.mp.	81795
50		exp Developing Countries/ or middle income countries.mp.	88815
51		exp Developing Countries/ or lower middle income country.mp.	77958
52		exp Developing Countries/ or lower middle income countries.mp.	78135
53		exp Developing Countries/ or upper middle income countries.mp.	77998
54		exp Developing Countries/ or upper middle income country.mp.	77880
55		Afghanistan.mp. or exp Afghanistan/	6592
56		Albania.mp. or exp Albania/	1248
57		Albania.mp. or exp Albania/	1248
58		American Samoa.mp. or exp American Samoa/	376
59		Angola.mp. or exp Angola/ ()	1583
60		Armenia.mp. or exp Armenia/	1842
61		Azerbaijan.mp. or exp Azerbaijan/	1762
62		Bangladesh.mp. or exp Bangladesh/	15112
63		Belarus.mp. or exp "Republic of Belarus"/	2679
64		Belize.mp. or exp Belize/	886
65		Benin.mp. or exp Benin/	3688
66		Bhutan.mp. or exp Bhutan/	809
67		Bolivia.mp. or exp Bolivia/	3888
68		(Bosnia and Herzegovina).mp. [mp=title, abstract, original title, name of substance word, subject heading word, floating sub-heading word, keyword heading word, organism supplementary concept word, protocol supplementary concept word, rare disease supplementary concept word, unique identifier, synonyms]	2655
69		Botswana.mp. or exp Botswana/	2537
70		Brazil.mp. or exp Brazil/	123075
71		Bulgaria.mp. or exp Bulgaria/	7816

72		Burkina Faso.mp. or exp Burkina Faso/	4440
73		Burundi.mp. or exp Burundi/	956
74		Cabo Verde.mp. or exp Cabo Verde/ ()	285
75		Cambodia.mp. or exp Cambodia/ ()	4644
76		Cameroon.mp. or exp Cameroon/ ()	7506
77		Central African Republic.mp. or exp Central African Republic/	1169
78		Chad.mp. or exp Chad/	1276
79		China.mp. or exp China/	276588
80		Colombia.mp. or exp Colombia/	14863
81		Comoros.mp. or exp Comoros/	508
82		exp "Democratic Republic of the Congo"/ or DRC.mp.	5714
83		Congo.mp. or exp Congo/ or exp "Democratic Republic of the Congo"/	14858
84		Costa Rica.mp. or exp Costa Rica/	5210
85		Ivory Coast.mp. or exp Cote d'Ivoire/	3830
86		Cuba.mp. or exp Cuba/	6536
87		Djibouti.mp. or exp Djibouti/	425
88		Dominica.mp. or exp Dominica/	445
89		Dominican Republic.mp. or exp Dominican Republic/	2294
90		Ecuador.mp. or exp Ecuador/	5474
91		exp Egypt/ or Egypt.mp.	20542
92		El Salvador.mp. or exp El Salvador/	1403
93		Equatorial Guinea.mp. or exp Equatorial Guinea/	476
94		Eritrea.mp. or exp Eritrea/	610
95		Ethiopia.mp. or exp Ethiopia/	16915
96		Fiji.mp. or exp Fiji/	1833
97		Gabon.mp. or exp Gabon/	2075
98		Gambia.mp. or exp Gambia/	3147
99		exp "Georgia (Republic)"/ or Georgia.mp.	16712
100		Ghana.mp. or exp Ghana/	11187
101		Grenada.mp. or exp Grenada/	325
102		Guatemala.mp. or exp Guatemala/	4218
103		Guinea.mp. or exp Guinea/	160323
104		Guinea-Bissau.mp. or exp Guinea-Bissau/	1223
105		Guyana.mp. or exp Guyana/	1181
106		Haiti.mp. or exp Haiti/	4001
107		Honduras.mp. or exp Honduras/	1895
108		India.mp. or exp India/	131814
109		Indonesia.mp. or exp Indonesia/	15431

110		Iran.mp. or exp Iran/	36150
111		exp Iraq/ or Iraq.mp.	9595
112		Jamaica.mp. or exp Jamaica/	4338
113		Jordan.mp. or exp Jordan/	6664
114		Kazakhstan.mp. or exp Kazakhstan/ (	3461
115		Kenya.mp. or exp Kenya/	21100
116		Kiribati.mp. or exp Micronesia/	2160
117		exp Korea/ or Korea.mp. or exp "Democratic People's Republic of Korea"/ or exp "Republic of Korea"/	64323
118		Kosovo.mp. or exp Kosovo/	840
119		exp Kyrgyzstan/ or Kyrgyz.mp.	1397
120		Lao.mp. or exp Laos/	3050
121		Lebanon.mp. or exp Lebanon/	5871
122		Lesotho.mp. or exp Lesotho/ ()	720
123		Liberia.mp. or exp Liberia/ ()	1809
124		Libya.mp. or exp Libya/	1526
125		exp "Macedonia (Republic)"/ or Macedonia.mp. ()	1212
126		Madagascar.mp. or exp Madagascar/	5100
127		Malawi.mp. or exp Malawi/	7388
128		Malaysia.mp. or exp Malaysia/	20092
129		Maldives.mp. or exp Indian Ocean Islands/	12867
130		Mali.mp. or exp Mali/	3870
131		Marshall Islands.mp. or exp Micronesia/	2167
132		Mauritania.mp. or exp Mauritania/	699
133		Mauritius.mp. or exp Mauritius/	985
134		exp Mexico/ or Mexico.mp.	57909
135		Micronesia.mp. or exp Micronesia/	2419
136		Moldova.mp. or exp Moldova/	1012
137		Mongolia.mp. or exp Mongolia/	4370
138		Montenegro.mp. or exp Montenegro/	799
139		Morocco.mp. or exp Morocco/	7636
140		Mozambique.mp. or exp Mozambique/	3784
141		Myanmar.mp. or exp Myanmar/	4020
142		Namibia.mp. or exp Namibia/	1713
143		Nauru.mp. or exp Micronesia/	2141
144		Nepal.mp. or exp Nepal/	11131
145		Nicaragua.mp. or exp Nicaragua/	2083
146		exp Niger/ or Niger.mp.	12614
147		exp Niger/ or Niger.mp.	12614
148		Nigeria.mp. or exp Nigeria/	34325

149		Pakistan.mp. or exp Pakistan/	23756
150		Papua New Guinea.mp. or exp Papua New Guinea/	5174
151		Paraguay.mp. or exp Paraguay/	1603
152		Peru.mp. or exp Peru/	12821
153		Philippines.mp. or exp Philippines/	11581
154		Romania.mp. or exp Romania/	12371
155		exp Russia/ or Russia.mp.	65165
156		Rwanda.mp. or exp Rwanda/	3437
157		exp Samoa/ or Samoa.mp. or exp "Independent State of Samoa"/	1107
158		Timor.mp.	662
159		Senegal.mp. or exp Senegal/	7527
160		Serbia.mp. or exp Serbia/ ()	5193
161		Sierra Leone.mp. or exp Sierra Leone/ ()	2347
162		Solomon Islands.mp. or exp Melanesia/ ()	6994
163		Somalia.mp. or exp Somalia/	2259
164		South Africa.mp. or exp South Africa/	52109
165		exp Sudan/ or exp South Sudan/ or Sudan.mp.	8651
166		Sri Lanka.mp. or exp Sri Lanka/	8063
167		Saint Lucia.mp. or exp Saint Lucia/	121
168		(Vincent and the Grenadines).mp. [mp=title, abstract, original title, name of substance word, subject heading word, floating sub-heading word, keyword heading word, organism supplementary concept word, protocol supplementary concept word, rare disease supplementary concept word, unique identifier, synonyms]	8651
169		exp Sudan/ or Sudan.mp.	1168
170		Suriname.mp. or exp Suriname/	921
171		Swaziland.mp. or exp Swaziland/	2927
172		Syria.mp. or exp Syria/	999
173		Tajikistan.mp. or exp Tajikistan/	14642
174		Tanzania.mp. or exp Tanzania/	35246
175		Thailand.mp. or exp Thailand/	399
176		Timor-Leste.mp. or exp Timor-Leste/	1678
177		Togo.mp. or exp Togo/	451
178		Tonga.mp. or exp Tonga/	90
179		Tunisia.mp. or Tunisia/	10240
180		exp Turkey/ or Turkey.mp. ()	52290
181		Turkmenistan.mp. or exp Turkmenistan/ ()	747
182		Tuvalu.mp. ()	65

183		Uganda.mp. or exp Uganda/	16034
184		Ukraine.mp. or exp Ukraine/ ()	17586
185		Uzbekistan.mp. or exp Uzbekistan/ ()	2255
186		Vanuatu.mp. or exp Vanuatu/ ()	672
187		Venezuela.mp. or exp Venezuela/	6794
188		exp Vietnam/ or Vietnam.mp.	17976
189		(West Bank and Gaza).mp. [mp=title, abstract, original title, name of substance word, subject heading word, floating sub-heading word, keyword heading word, organism supplementary concept word, protocol supplementary concept word, rare disease supplementary concept word, unique identifier, synonyms] ()	205
190		Yemen.mp. or exp Yemen/	2008
191		Zambia.mp. or exp Zambia/	6101
192		Zimbabwe.mp. or exp Zimbabwe/	7411
193		43 or 44 or 45 or 46 or 47 or 48 or 49 or 50 or 51 or 52 or 53 or 54	138004
194		55 or 56 or 57 or 58 or 59 or 60 or 61 or 62 or 63 or 64 or 65 or 66 or 67 or 68 or 69 or 70 or 71 or 72 or 73 or 74 or 75 or 76 or 77 or 78 or 79 or 80 or 81 or 82 or 83 or 84 or 85 or 86 or 87 or 88 or 89 or 90 or 91 or 92 or 93 or 94 or 95 or 96 or 97 or 98 or 99 or 100 or 101 or 102 or 103 or 104 or 105 or 106 or 107 or 108 or 109 or 110 or 111 or 112 or 113 or 114 or 115 or 116 or 117 or 118 or 119 or 120 or 121 or 122 or 123 or 124 or 125 or 126 or 127 or 128 or 129 or 130 or 131 or 132 or 133 or 134 or 135 or 136 or 137 or 138 or 139 or 140 or 141 or 142 or 143 or 144 or 145 or 146 or 147 or 148 or 149 or 150 or 151 or 152 or 153 or 154 or 155 or 156 or 157 or 158 or 159 or 160 or 161 or 162 or 163 or 164 or 165 or 166 or 167 or 168 or 169 or 170 or 171 or 172 or 173 or 174 or 175 or 176 or 177 or 178 or 179 or 180 or 181 or 182 or 183 or 184 or 185 or 186 or 187 or 188 or 189 or 190 or 191 or 192	1530131
195		193 or 194	1590932
196		18 and 32 and 41 and 195	425

Final search output: 425

Search Summary-2

Name of the database

EMBASE (via Ovid)

Date of search

08 October 2021

Timeline

Not applicable

Search Language

Line #	Theme	Search Language	Output #
1.	Hypoxaemia	hypoxemia.mp. or exp hypoxemia	185053
2.		hypoxaemia.mp. or exp hypoxemia	179756
3.		anoxia.mp. or exp anoxia	18565
4.		hypoxia.mp. or exp hypoxia	220645
5.		exp pulse oximetry/ or exp oximetry/ or oximetry.mp.	32644
6.		oximeter.mp. or exp pulse oximeter/ or exp oximeter	9570
7.		exp pulse oximeter/ or oximetre.mp.	4696
8.		oxymetry.mp. or exp oximetry	29785
9.		oxymeter.mp. or exp oximeter	7032
10.		oxymetre.mp.	10
11.		blood oxygen saturation.mp. or exp blood oxygen tension/ or exp oxygen blood level	13047
12.		arterial oxygen saturation.mp. or exp arterial oxygen saturation/ or exp arterial oxygen tension	23200
13.		exp arterial oxygen tension/ or exp arterial gas/ or exp blood gas analysis/ or exp blood gas/ or blood gas monitoring.mp	96898

14.		blood gas analysis.mp. or exp blood gas analysis	26011
15.		exp arterial gas/ or exp blood gas analysis/ or analysis blood gas.mp. or exp blood gas	96821
16.		exp arterial oxygen saturation/ or exp oxygen saturation/ or exp pulse oximetry/ or SpO2.mp. or exp oximetry	98190
17.		exp arterial oxygen saturation/ or exp oxygen saturation/ or exp pulse oximetry/ or SPO2.mp. or exp oximetry	98190
18.		1 or 2 or 3 or 4 or 5 or 6 or 7 or 8 or 9 or 10 or 11 or 12 or 13 or 14 or 15 or 16 or 17	419024
19.	Pneumo nia	exp pneumonia/ or exp bacterial pneumonia/ or pneumonia.mp. or exp virus pneumonia	378952
20.		exp pneumonia/ or severe pneumonia.mp. or exp bacterial pneumonia	335707
21.		severe disease.mp.	32437
22.		very severe disease.mp.	386
23.		bronchiolitis.mp. or exp bronchiolitis/ or exp viral bronchiolitis/ or exp bronchiolitis obliterans organizing pneumonia	26335
24.		acute respiratory infection.mp. or exp respiratory tract infection	422221
25.		exp pneumonia/ or exp respiratory tract infection/ or ARI.mp.	623374
26.		acute respiratory infections.mp. or exp respiratory tract infection	422453
27.		acute respiratory tract infection.mp. or exp respiratory tract infection	421869
28.		exp respiratory tract infection/ or ARTI.mp	422904
29.		acute respiratory tract infections.mp. or exp respiratory tract infection	421932
30.		respiratory tract disease/ or exp acute respiratory tract disease/ or exp respiratory tract infection/ or exp<br><br>upper respiratory tract congestion/ or respiratory illness.mp.	478690
31.		respiratory tract disease/ or exp acute respiratory tract disease/ or exp respiratory tract infection/ or exp	477203

		upper respiratory tract congestion/ or respiratory illnesses.mp.	
32.		19 or 20 or 21 or 22 or 23 or 24 or 25 or 26 or 27 or 28 or 29 or 30 or 31	757123
33.	children under 5 years of age	exp hospitalized child/ or child*.mp. or exp child care/ or exp child hospitalization/ or exp child health/ or<br><br>exp child health care	274980<br>1
34.		exp hospitalized child/ or child*.mp. or exp child hospitalization/ or exp child health/ or exp child health<br><br>care	274693<br>3
35.		exp infant care/ or exp hospitalized infant/ or infant*.mp.	868324
36.		exp infant care/ or exp hospitalized infant/ or infant*.mp.	868324
37.		Pediatric.mp. or exp pediatrics	575529
38.		Pediatrics.mp. or exp pediatrics	154873
39.		Paediatric.mp. or exp pediatrics	208410
40.		Paediatrics.mp. or exp pediatrics	118440
41.		33 or 34 or 35 or 36 or 37 or 38 or 39 or 40	320399<br>7
42.	Low and Middle Income Country	exp developing country/ or developing countr*.mp.	140390
43.		low-and middle income countr*.mp.	22831
44.		exp middle income country/ or LMIC*.mp. or exp developing country	109400
45.		exp middle income country/ or exp low income country/ or low income countr*.mp. or exp developing country	114651
46.		low income country/ or exp middle income country/ or exp developing country/ or middle income countr*.mp.	124293
47.		exp developing country/ or exp low income country/ or lower middle income countr*.mp.	102519

48.		upper middle income countr*.mp.	661
49.		Afghanistan.mp. or exp Afghanistan	9229
50.		Albania.mp. or exp Albania	2326
51.		American Samoa.mp. or exp American Samoa	470
52.		Angola.mp. or exp Angola	1968
53.		Armenia.mp. or exp Armenia	2288
54.		Azerbaijan.mp. or exp Azerbaijan	2495
55.		Bangladesh.mp. or exp Bangladesh	22201
56.		Belarus.mp. or exp Belarus	3346
57.		Belize.mp. or exp Belize	1098
58.		Benin.mp. or exp Benin	5566
59.		Bhutan.mp. or exp Bhutan	1185
60.		Bolivia.mp. or exp Bolivia	4793
61.		(Bosnia and Herzegovina).mp. [mp=title, abstract, heading word, drug trade name, original title, device manufacturer, drug manufacturer, device trade name, keyword, floating subheading word, candidate term word]	3562
62.		Botswana.mp. or exp Botswana	3454
63.		exp Brazil/ or Brazil.mp.	156620
64.		Bulgaria.mp. or exp Bulgaria	9681
65.		Burkina Faso.mp. or exp Burkina Faso	5913
66.		Burundi.mp. or exp Burundi	1173
67.		Cabo Verde.mp. or exp Cape Verde	479
68.		Cambodia.mp. or exp Cambodia	6483
69.		Cameroon.mp. or exp Cameroon	9641

70.		Central African Republic.mp. or exp Central African Republic	1349
71.		Chad.mp. or exp Chad	1734
72.		China.mp. or exp China	405192
73.		Colombia.mp. or exp Colombia	25591
74.		Comoros.mp. or exp Comoros	559
75.		exp Democratic Republic Congo/ or exp Congo/ or Congo.mp.	20650
76.		Costa Rica.mp. or exp Costa Rica	6278
77.		Ivory Coast.mp. or exp Cote d'Ivoire	3959
78.		Cuba.mp. or Cuba	8837
79.		Djibouti.mp. or exp Djibouti)	553
80.		Djibouti.mp. or exp Djibouti	553
81.		exp "Dominican (Dominica)"/ or Dominica.mp. or exp Dominica	764
82.		Dominican Republic.mp. or exp Dominican Republic	3271
83.		Ecuador.mp. or exp Ecuador	7640
84.		exp Egypt/ or Egypt.mp	30517
85.		exp Egypt/ or Egypt.mp	30517
86.		El Salvador.mp. or exp El Salvador	2423
87.		Equatorial Guinea.mp. or exp Equatorial Guinea	705
88.		Eritrea.mp. or exp Eritrea	840
89.		Ethiopia.mp. or exp Ethiopia	23227
90.		Fiji.mp. or exp Fiji	2666
91.		Gabon.mp. or exp Gabon	2465
92.		Gambia.mp. or exp Gambia	3471

93.		Georgia.mp. or exp "Georgia (republic)"/ or exp "South Georgia and the South Sandwich Islands"	12977
94.		Ghana.mp. or exp Ghana)	15280
95.		Grenada.mp. or exp Grenada)	458
96.		Guatemala.mp. or exp Guatemala	5258
97.		exp Guinea-Bissau/ or Guinea.mp. or exp Guinea	112538
98.		Guinea-Bissau.mp. or exp Guinea-Bissau	1377
99.		Guyana.mp. or exp Guyana	1447
100		Haiti.mp. or exp Haiti	5084
101		Honduras.mp. or exp Honduras	2574
102		exp India/ or India.mp.	231518
103		exp India/ or India.mp.	231518
104		exp India/ or India.mp.	231518
105		Indonesia.mp. or exp Indonesia	28042
106		Iran.mp. or exp Iran	76031
107		Iraq.mp. or exp Iraq	15085
108		Jamaica.mp. or exp Jamaica	4562
109		Jordan.mp. or exp Jordan	11118
110		Kazakhstan.mp. or exp Kazakhstan	4775
111		Kenya.mp. or exp Kenya	26424
112		Kiribati.mp. or exp Kiribati	250
113		exp North Korea/ or Korea.mp. or exp Korea/ or exp South Korea	103268
114		Kosovo.mp. or exp Kosovo	1431
115		Kyrgyz Republic.mp. or exp Kyrgyzstan	1302

116		Laos/ or Lao PDR.mp.	2756
117		Lebanon.mp. or exp Lebanon	7757
118		Lesotho.mp. or exp Lesotho	1009
119		Liberia.mp. or exp Liberia	2270
120		Libya.mp. or exp Libyan Arab Jamahiriya	2177
121		exp "Macedonia (republic)"/ or Macedonia.mp.	2357
122		Madagascar.mp. or exp Madagascar	6097
123		Malawi.mp. or exp Malawi	9928
124		Malaysia.mp. or exp Malaysia	31159
125		Maldives.mp. or exp Maldives	551
126		Mali.mp. or exp Mali	5296
127		exp Marshall Islands/ or Marshall Islands.mp.	372
128		Mauritania.mp. or exp Mauritania	902
129		Mauritius.mp. or exp Mauritius	1345
130		exp Mexico/ or exp Mexico City/ or Mexico.mp.	69945
131		Micronesia.mp. or exp "Federated States of Micronesia"	1261
132		Micronesia.mp. or exp "Federated States of Micronesia"	1261
133		Moldova.mp. or exp Moldova)	1441
134		exp Mongolia/ or Mongolia.mp.	6282
135		Montenegro.mp. or exp "Montenegro (republic)"	1461
136		Morocco.mp. or exp Morocco	10176
137		Mozambique.mp. or exp Mozambique	5141
138		Myanmar.mp. or exp Myanmar	5561
139		Namibia.mp. or exp Namibia	2342

140		Nauru.mp. or exp Nauru	191
141		Nepal.mp. or exp Nepal	15738
142		Nicaragua.mp. or exp Nicaragua	2880
143		exp Niger/ or Niger.mp.	22149
144		Nigeria.mp. or exp Nigeria	46805
145		Pakistan.mp. or exp Pakistan)	38546
146		Papua New Guinea.mp. or exp Papua New Guinea	6455
147		Paraguay.mp. or exp Paraguay	2289
148		Peru.mp. or exp Peru	17534
149		Philippines.mp. or exp Philippines	14269
150		Romania.mp. or exp Romania	15655
151		Russian Federation.mp. or exp Russian Federation	58634
152		Rwanda.mp. or exp Rwanda	4780
153		exp Samoa/ or Samoa.mp.	1258
154		(Sao Tome and Principe).mp. [mp=title, abstract, heading word, drug trade name, original title, device manufacturer, drug manufacturer, device trade name, keyword, floating subheading word, candidate term word]	223
155		exp Senegalia senegal/ or exp Senegal/ or Senegal.mp.	8356
156		Serbia.mp. or exp Serbia	8866
157		Sierra Leone.mp. or exp Sierra Leone	3277
158		Solomon Islands.mp. or exp Solomon Islands	998
159		Somalia.mp. or exp Somalia	2583
160		South Africa.mp. or exp South Africa	63344
161		South Sudan.mp. or exp Sudan/ or exp South Sudan/	6815

162		Sri Lanka.mp. or exp Sri Lanka	10832
163		Saint Lucia.mp. or exp Saint Lucia	154
164		(Saint Vincent and the Grenadines).mp. [mp=title, abstract, heading word, drug trade name, original title,<br><br>device manufacturer, drug manufacturer, device trade name, keyword, floating subheading word, candidate term word]	90
165		exp Sudan/ or Sudan.mp. or exp South Sudan	10875
166		Suriname.mp. or exp Suriname	1225
167		Swaziland.mp. or exp Swaziland	1565
168		Syrian Arab Republic.mp. or exp Syrian Arab Republic/	2965
169		Tajikistan.mp. or exp Tajikistan	1116
170		Tanzania.mp. or exp Tanzania	18529
171		Thailand.mp. or exp Thailand	45080
172		Timor-Leste.mp. or exp Timor-Leste	760
173		Togo.mp. or exp Togo	2096
174		exp Tonga/ or Tonga.mp.	598
175		Tunisia.mp. or exp Tunisia	12789
176		Turkey.mp.	75844
177		Turkmenistan.mp. or exp Turkmenistan	599
178		Tuvalu.mp. or exp Tuvalu	102
179		Uganda.mp. or exp Uganda	21051
180		Ukraine.mp. or exp Ukraine	15286
181		Uzbekistan.mp. or exp Uzbekistan	2386
182		Vanuatu.mp. or exp Vanuatu	822
183		Venezuela.mp. or exp Venezuela	7906

184		Vietnam.mp. or exp Viet Nam	22219
185		exp Palestine/ or Gaza.mp. or exp Palestinian	3329
186		Yemen.mp. or exp Yemen	2713
187		Zambia.mp. or exp Zambia	7731
188		Zimbabwe.mp. or exp Zimbabwe	8083
189		42 or 43 or 44 or 45 or 46 or 47 or 48 or 49 or 50 or 51 or 52 or 53 or 54 or 55 or 56 or 57 or 58 or 59 or 60<br>or 61 or 62 or 63 or 64 or 65 or 66 or 67 or 68 or 69 or 70 or 71 or 72 or 73 or 74 or 75 or 76 or 77 or 78 or 79 or 80<br>or 81 or 82 or 83 or 84 or 85 or 86 or 87 or 88 or 89 or 90 or 91 or 92 or 93 or 94 or 95 or 96 or 97 or 98 or 99 or 100<br>or 101 or 102 or 103 or 104 or 105 or 106 or 107 or 108 or 109 or 110 or 111 or 112 or 113 or 114 or 115 or 116 or 117<br>or 118 or 119 or 120 or 121 or 122 or 123 or 124 or 125 or 126 or 127 or 128 or 129 or 130 or 131 or 132 or 133 or 134<br>or 135 or 136 or 137 or 138 or 139 or 140 or 141 or 142 or 143 or 144 or 145 or 146 or 147 or 148 or 149 or 150 or 151<br>or 152 or 153 or 154 or 155 or 156 or 157 or 158 or 159 or 160 or 161 or 162 or 163 or 164 or 165 or 166 or 167 or 168<br>or 169 or 170 or 171 or 172 or 173 or 174 or 175 or 176 or 177 or 178 or 179 or 180 or 181 or 182 or 183 or 184 or 185<br>or 186 or 187 or 188	2085410
190		18 and 32 and 41 and 189	807

Final search output: 807

Search Summary-3

Name of the database

GLOBAL HEALTH (via Ovid)

Date of search

05 February 2019

Timeline

Not applicable

Search Language

Line #	Theme	Search Language	Output #
1.	Hypoxaemia	hypoxemia.mp. [mp=abstract, title, original title, broad terms, heading words, identifiers, cabicodes]	505
2.		hypoxaemia.mp. [mp=abstract, title, original title, broad terms, heading words, identifiers, cabicodes]	217
3.		anoxia.mp. [mp=abstract, title, original title, broad terms, heading words, identifiers, cabicodes]	393
4.		hypoxia.mp. [mp=abstract, title, original title, broad terms, heading words, identifiers, cabicodes]	4325
5.		oximetry.mp. [mp=abstract, title, original title, broad terms, heading words, identifiers, cabicodes]	350
6.		oximeter.mp. [mp=abstract, title, original title, broad terms, heading words, identifiers, cabicodes]	97
7.		oximetre.mp. [mp=abstract, title, original title, broad terms, heading words, identifiers, cabicodes]	1
8.		oxymetry.mp. [mp=abstract, title, original title, broad terms, heading words, identifiers, cabicodes]	12
9.		oxymeter.mp. [mp=abstract, title, original title, broad terms, heading words, identifiers, cabicodes]	6
10.		oxymetre.mp. [mp=abstract, title, original title, broad terms, heading words, identifiers, cabicodes]	0
11.		blood oxygen saturation.mp. [mp=abstract, title, original title, broad terms, heading words, identifiers, cabicodes]	83
12.		arterial oxygen saturation.mp. [mp=abstract, title, original title, broad terms, heading words, identifiers, cabicodes]	111
13.		blood gas monitoring.mp. [mp=abstract, title, original title, broad terms, heading words, identifiers, cabicodes]	6
14.		blood gas analysis.mp. [mp=abstract, title, original title, broad terms, heading words, identifiers, cabicodes]	291

15.		analysis blood gas.mp. [mp=abstract, title, original title, broad terms, heading words, identifiers, cabicodes]	0
16.		SpO2.mp. [mp=abstract, title, original title, broad terms, heading words, identifiers, cabicodes]	395
17.		SPO2.mp. [mp=abstract, title, original title, broad terms, heading words, identifiers, cabicodes]	395
18.		1 or 2 or 3 or 4 or 5 or 6 or 7 or 8 or 9 or 10 or 11 or 12 or 13 or 14 or 15 or 16 or 17	6094
19.	Pneumonia	pneumonia.mp. [mp=abstract, title, original title, broad terms, heading words, identifiers, cabicodes]	23912
20.		severe pneumonia.mp. [mp=abstract, title, original title, broad terms, heading words, identifiers, cabicodes]	1056
21.		severe disease.mp. [mp=abstract, title, original title, broad terms, heading words, identifiers, cabicodes]	3526
22.		very severe disease.mp. [mp=abstract, title, original title, broad terms, heading words, identifiers, cabicodes]	46
23.		bronchiolitis.mp. [mp=abstract, title, original title, broad terms, heading words, identifiers, cabicodes]	1597
24.		acute respiratory infection.mp. [mp=abstract, title, original title, broad terms, heading words, identifiers, cabicodes]	846
25.		ARI.mp. [mp=abstract, title, original title, broad terms, heading words, identifiers, cabicodes]	846
26.		acute respiratory infections.mp. [mp=abstract, title, original title, broad terms, heading words, identifiers, cabicodes]	985
27.		acute respiratory tract infections.mp. [mp=abstract, title, original title, broad terms, heading words, identifiers, cabicodes]	368
28.		ARTI.mp. [mp=abstract, title, original title, broad terms, heading words, identifiers, cabicodes]	149
29.		acute respiratory tract infections.mp. [mp=abstract, title, original title, broad terms, heading words, identifiers, cabicodes]	368
30.		respiratory illness*.mp. [mp=abstract, title, original title, broad terms, heading words, identifiers, cabicodes]	2031
31.		respiratory illnesses*.mp. [mp=abstract, title, original title, broad terms, heading words, identifiers, cabicodes]	577
32.		19 or 20 or 21 or 22 or 23 or 24 or 25 or 26 or 27 or 28 or 29 or 30 or 31	31692
33.	children under 5 years of age	child.mp. [mp=abstract, title, original title, broad terms, heading words, identifiers, cabicodes]	55244
34.		children.mp. [mp=abstract, title, original title, broad terms, heading words, identifiers, cabicodes]	209960
35.		infant.mp. [mp=abstract, title, original title, broad terms, heading words, identifiers, cabicodes]	34055
36.		infants.mp. [mp=abstract, title, original title, broad terms, heading words, identifiers, cabicodes]	65307
37.		pediatric.mp. [mp=abstract, title, original title, broad terms, heading words, identifiers, cabicodes]	22238

38.		pediatrics.mp. [mp=abstract, title, original title, broad terms, heading words, identifiers, cabicodes]	4805
39.		paediatric.mp. [mp=abstract, title, original title, broad terms, heading words, identifiers, cabicodes]	8904
40.		paediatrics.mp. [mp=abstract, title, original title, broad terms, heading words, identifiers, cabicodes]	3341
41.		33 or 34 or 35 or 36 or 37 or 38 or 39 or 40	263930
42.	Low and Middle Income Country	Afghanistan.mp. [mp=abstract, title, original title, broad terms, heading words, identifiers, cabicodes]	1515
43.		Albania.mp. [mp=abstract, title, original title, broad terms, heading words, identifiers, cabicodes]	735
44.		Albania.mp. [mp=abstract, title, original title, broad terms, heading words, identifiers, cabicodes]	735
45.		American Samoa.mp. [mp=abstract, title, original title, broad terms, heading words, identifiers, cabicodes]	112
46.		Angola.mp. [mp=abstract, title, original title, broad terms, heading words, identifiers, cabicodes]	672
47.		Armenia.mp. [mp=abstract, title, original title, broad terms, heading words, identifiers, cabicodes]	326
48.		Azerbaijan.mp. [mp=abstract, title, original title, broad terms, heading words, identifiers, cabicodes]	546
49.		Bangladesh.mp. [mp=abstract, title, original title, broad terms, heading words, identifiers, cabicodes]	7717
50.		Belarus.mp. [mp=abstract, title, original title, broad terms, heading words, identifiers, cabicodes]	568
51.		Belize.mp. [mp=abstract, title, original title, broad terms, heading words, identifiers, cabicodes]	181
52.		Benin.mp. [mp=abstract, title, original title, broad terms, heading words, identifiers, cabicodes]	2179
53.		Bhutan.mp. [mp=abstract, title, original title, broad terms, heading words, identifiers, cabicodes]	417
54.		Bolivia.mp. [mp=abstract, title, original title, broad terms, heading words, identifiers, cabicodes]	1276
55.		(Bosnia and Herzegovina).mp. [mp=abstract, title, original title, broad terms, heading words, identifiers, cabicodes]	578
56.		Botswana.mp. [mp=abstract, title, original title, broad terms, heading words, identifiers, cabicodes]	1097
57.		Brazil.mp. [mp=abstract, title, original title, broad terms, heading words, identifiers, cabicodes]	57836
58.		Bulgaria.mp. [mp=abstract, title, original title, broad terms, heading words, identifiers, cabicodes]	3181
59.		Burkina Faso.mp. [mp=abstract, title, original title, broad terms, heading words, identifiers, cabicodes]	2593
60.		Burundi.mp. [mp=abstract, title, original title, broad terms, heading words, identifiers, cabicodes]	291

61.		Cabo Verde.mp. [mp=abstract, title, original title, broad terms, heading words, identifiers, cabicodes]	17
62.		Cambodia.mp. [mp=abstract, title, original title, broad terms, heading words, identifiers, cabicodes]	2188
63.		Cameroon.mp. [mp=abstract, title, original title, broad terms, heading words, identifiers, cabicodes]	3674
64.		Central African Republic.mp. [mp=abstract, title, original title, broad terms, heading words, identifiers, cabicodes]	398
65.		Chad.mp. [mp=abstract, title, original title, broad terms, heading words, identifiers, cabicodes]	501
66.		China.mp. [mp=abstract, title, original title, broad terms, heading words, identifiers, cabicodes]	140022
67.		Colombia.mp. [mp=abstract, title, original title, broad terms, heading words, identifiers, cabicodes]	6489
68.		Comoros.mp. [mp=abstract, title, original title, broad terms, heading words, identifiers, cabicodes]	168
69.		(Democratic Republic of Congo or DR Congo or DRC or the Congo).mp. [mp=abstract, title, original title, broad terms, heading words, identifiers, cabicodes]	4453
70.		Congo.mp. [mp=abstract, title, original title, broad terms, heading words, identifiers, cabicodes]	4298
71.		Costa Rica.mp. [mp=abstract, title, original title, broad terms, heading words, identifiers, cabicodes]	1257
72.		Ivory Coast.mp. [mp=abstract, title, original title, broad terms, heading words, identifiers, cabicodes]	1864
73.		Cuba.mp. [mp=abstract, title, original title, broad terms, heading words, identifiers, cabicodes]	2711
74.		Djibouti.mp. [mp=abstract, title, original title, broad terms, heading words, identifiers, cabicodes]	159
75.		Dominica.mp. [mp=abstract, title, original title, broad terms, heading words, identifiers, cabicodes]	148
76.		Dominican Republic.mp. [mp=abstract, title, original title, broad terms, heading words, identifiers, cabicodes]	598
77.		Ecuador.mp. [mp=abstract, title, original title, broad terms, heading words, identifiers, cabicodes]	1523
78.		Egypt.mp. [mp=abstract, title, original title, broad terms, heading words, identifiers, cabicodes]	8075
79.		El Salvador.mp. [mp=abstract, title, original title, broad terms, heading words, identifiers, cabicodes]	471
80.		Equatorial Guinea.mp. [mp=abstract, title, original title, broad terms, heading words, identifiers, cabicodes]	201
81.		Eritrea.mp. [mp=abstract, title, original title, broad terms, heading words, identifiers, cabicodes]	227
82.		Ethiopia.mp. [mp=abstract, title, original title, broad terms, heading words, identifiers, cabicodes]	7844

83.		Fiji.mp. [mp=abstract, title, original title, broad terms, heading words, identifiers, cabicodes]	382
84.		Gabon.mp. [mp=abstract, title, original title, broad terms, heading words, identifiers, cabicodes]	777
85.		Gambia.mp. [mp=abstract, title, original title, broad terms, heading words, identifiers, cabicodes]	948
86.		Georgia.mp. [mp=abstract, title, original title, broad terms, heading words, identifiers, cabicodes]	3603
87.		Ghana.mp. [mp=abstract, title, original title, broad terms, heading words, identifiers, cabicodes]	5846
88.		Grenada.mp. [mp=abstract, title, original title, broad terms, heading words, identifiers, cabicodes]	134
89.		Guatemala.mp. [mp=abstract, title, original title, broad terms, heading words, identifiers, cabicodes]	1227
90.		(Guinea-Bissau or Guinea Bissau).mp. [mp=abstract, title, original title, broad terms, heading words, identifiers, cabicodes]	414
91.		Guyana.mp. [mp=abstract, title, original title, broad terms, heading words, identifiers, cabicodes]	281
92.		Haiti.mp. [mp=abstract, title, original title, broad terms, heading words, identifiers, cabicodes]	1216
93.		Honduras.mp. [mp=abstract, title, original title, broad terms, heading words, identifiers, cabicodes]	518
94.		India.mp. [mp=abstract, title, original title, broad terms, heading words, identifiers, cabicodes]	71629
95.		Indonesia.mp. [mp=abstract, title, original title, broad terms, heading words, identifiers, cabicodes]	6099
96.		Iran.mp. [mp=abstract, title, original title, broad terms, heading words, identifiers, cabicodes]	39859
97.		Iraq.mp. [mp=abstract, title, original title, broad terms, heading words, identifiers, cabicodes]	2448
98.		Jamaica.mp. [mp=abstract, title, original title, broad terms, heading words, identifiers, cabicodes]	819
99.		Jordan.mp. [mp=abstract, title, original title, broad terms, heading words, identifiers, cabicodes]	2043
100.		Kazakhstan.mp. [mp=abstract, title, original title, broad terms, heading words, identifiers, cabicodes]	806
101.		Kenya.mp. [mp=abstract, title, original title, broad terms, heading words, identifiers, cabicodes]	8859
102.		Kiribati.mp. [mp=abstract, title, original title, broad terms, heading words, identifiers, cabicodes]	70
103.		Korea.mp. [mp=abstract, title, original title, broad terms, heading words, identifiers, cabicodes]	19978
104.		Kosovo.mp. [mp=abstract, title, original title, broad terms, heading words, identifiers, cabicodes]	400
105.		Kyrgyz.mp. [mp=abstract, title, original title, broad terms, heading words, identifiers, cabicodes]	47

106.		Lao.mp. [mp=abstract, title, original title, broad terms, heading words, identifiers, cabicodes]	1180
107.		Lebanon.mp. [mp=abstract, title, original title, broad terms, heading words, identifiers, cabicodes]	1592
108.		Lesotho.mp. [mp=abstract, title, original title, broad terms, heading words, identifiers, cabicodes]	369
109.		Liberia.mp. [mp=abstract, title, original title, broad terms, heading words, identifiers, cabicodes]	694
110.		Libya.mp. [mp=abstract, title, original title, broad terms, heading words, identifiers, cabicodes]	575
111.		Macedonia.mp. [mp=abstract, title, original title, broad terms, heading words, identifiers, cabicodes]	849
112.		Madagascar.mp. [mp=abstract, title, original title, broad terms, heading words, identifiers, cabicodes]	1400
113.		Malawi.mp. [mp=abstract, title, original title, broad terms, heading words, identifiers, cabicodes]	3882
114.		Malaysia.mp. [mp=abstract, title, original title, broad terms, heading words, identifiers, cabicodes]	8375
115.		Maldives.mp. [mp=abstract, title, original title, broad terms, heading words, identifiers, cabicodes]	109
116.		Mali.mp. [mp=abstract, title, original title, broad terms, heading words, identifiers, cabicodes]	1738
117.		Marshall Islands.mp. [mp=abstract, title, original title, broad terms, heading words, identifiers, cabicodes]	87
118.		Mauritania.mp. [mp=abstract, title, original title, broad terms, heading words, identifiers, cabicodes]	231
119.		Mauritius.mp. [mp=abstract, title, original title, broad terms, heading words, identifiers, cabicodes]	327
120.		Mexico.mp. [mp=abstract, title, original title, broad terms, heading words, identifiers, cabicodes]	13948
121.		Micronesia.mp. [mp=abstract, title, original title, broad terms, heading words, identifiers, cabicodes]	469
122.		Moldova.mp. [mp=abstract, title, original title, broad terms, heading words, identifiers, cabicodes]	349
123.		Mongolia.mp. [mp=abstract, title, original title, broad terms, heading words, identifiers, cabicodes]	1699
124.		Montenegro.mp. [mp=abstract, title, original title, broad terms, heading words, identifiers, cabicodes]	487
125.		Morocco.mp. [mp=abstract, title, original title, broad terms, heading words, identifiers, cabicodes]	4146
126.		Mozambique.mp. [mp=abstract, title, original title, broad terms, heading words, identifiers, cabicodes]	1877
127.		Myanmar.mp. [mp=abstract, title, original title, broad terms, heading words, identifiers, cabicodes]	2052
128.		Namibia.mp. [mp=abstract, title, original title, broad terms, heading words, identifiers, cabicodes]	601

129.		Nauru.mp. [mp=abstract, title, original title, broad terms, heading words, identifiers, cabicodes]	44
130.		Nepal.mp. [mp=abstract, title, original title, broad terms, heading words, identifiers, cabicodes]	4514
131.		Nicaragua.mp. [mp=abstract, title, original title, broad terms, heading words, identifiers, cabicodes]	732
132.		Niger.mp. [mp=abstract, title, original title, broad terms, heading words, identifiers, cabicodes]	7584
133.		Pakistan.mp. [mp=abstract, title, original title, broad terms, heading words, identifiers, cabicodes]	11064
134.		Papua New Guinea.mp. [mp=abstract, title, original title, broad terms, heading words, identifiers, cabicodes]	1144
135.		Paraguay.mp. [mp=abstract, title, original title, broad terms, heading words, identifiers, cabicodes]	621
136.		Peru.mp. [mp=abstract, title, original title, broad terms, heading words, identifiers, cabicodes]	4376
137.		Philippines.mp. [mp=abstract, title, original title, broad terms, heading words, identifiers, cabicodes]	2927
138.		Romania.mp. [mp=abstract, title, original title, broad terms, heading words, identifiers, cabicodes]	5990
139.		Russia.mp. [mp=abstract, title, original title, broad terms, heading words, identifiers, cabicodes]	7296
140.		Rwanda.mp. [mp=abstract, title, original title, broad terms, heading words, identifiers, cabicodes]	1357
141.		Samoa.mp. [mp=abstract, title, original title, broad terms, heading words, identifiers, cabicodes]	277
142.		(Timor-Liste or Timor Liste or Timor).mp. [mp=abstract, title, original title, broad terms, heading words, identifiers, cabicodes]	286
143.		Senegal.mp. [mp=abstract, title, original title, broad terms, heading words, identifiers, cabicodes]	2443
144.		Serbia.mp. [mp=abstract, title, original title, broad terms, heading words, identifiers, cabicodes]	3061
145.		Sierra Leone.mp. [mp=abstract, title, original title, broad terms, heading words, identifiers, cabicodes]	999
146.		Solomon Islands.mp. [mp=abstract, title, original title, broad terms, heading words, identifiers, cabicodes]	263
147.		Somalia.mp. [mp=abstract, title, original title, broad terms, heading words, identifiers, cabicodes]	644
148.		South Africa.mp. [mp=abstract, title, original title, broad terms, heading words, identifiers, cabicodes]	17163
149.		South Sudan.mp. [mp=abstract, title, original title, broad terms, heading words, identifiers, cabicodes]	386
150.		Sri Lanka.mp. [mp=abstract, title, original title, broad terms, heading words, identifiers, cabicodes]	2841
151.		Saint Lucia.mp. [mp=abstract, title, original title, broad terms, heading words, identifiers, cabicodes]	65

152.		(Vincent and the Grenadines).mp. [mp=abstract, title, original title, broad terms, heading words, identifiers, cabicodes]	44
153.		Sudan.mp. [mp=abstract, title, original title, broad terms, heading words, identifiers, cabicodes]	3106
154.		Suriname.mp. [mp=abstract, title, original title, broad terms, heading words, identifiers, cabicodes]	279
155.		Swaziland.mp. [mp=abstract, title, original title, broad terms, heading words, identifiers, cabicodes]	501
156.		Syria.mp. [mp=abstract, title, original title, broad terms, heading words, identifiers, cabicodes]	820
157.		Tajikistan.mp. [mp=abstract, title, original title, broad terms, heading words, identifiers, cabicodes]	278
158.		Tanzania.mp. [mp=abstract, title, original title, broad terms, heading words, identifiers, cabicodes]	6627
159.		Thailand.mp. [mp=abstract, title, original title, broad terms, heading words, identifiers, cabicodes]	13856
160.		Timor-Leste.mp. [mp=abstract, title, original title, broad terms, heading words, identifiers, cabicodes]	173
161.		Togo.mp. [mp=abstract, title, original title, broad terms, heading words, identifiers, cabicodes]	707
162.		Tonga.mp. [mp=abstract, title, original title, broad terms, heading words, identifiers, cabicodes]	132
163.		Tunisia.mp. [mp=abstract, title, original title, broad terms, heading words, identifiers, cabicodes]	3836
164.		Turkey.mp. [mp=abstract, title, original title, broad terms, heading words, identifiers, cabicodes]	25147
165.		Turkmenistan.mp. [mp=abstract, title, original title, broad terms, heading words, identifiers, cabicodes]	90
166.		Tuvalu.mp. [mp=abstract, title, original title, broad terms, heading words, identifiers, cabicodes]	29
167.		Uganda.mp. [mp=abstract, title, original title, broad terms, heading words, identifiers, cabicodes]	7227
168.		Ukraine.mp. [mp=abstract, title, original title, broad terms, heading words, identifiers, cabicodes]	1415
169.		Uzbekistan.mp. [mp=abstract, title, original title, broad terms, heading words, identifiers, cabicodes]	337
170.		Vanuatu.mp. [mp=abstract, title, original title, broad terms, heading words, identifiers, cabicodes]	211
171.		Venezuela.mp. [mp=abstract, title, original title, broad terms, heading words, identifiers, cabicodes]	2273
172.		Vietnam.mp. [mp=abstract, title, original title, broad terms, heading words, identifiers, cabicodes]	5464
173.		(West Bank and Gaza).mp. [mp=abstract, title, original title, broad terms, heading words, identifiers, cabicodes]	101
174.		Yemen.mp. [mp=abstract, title, original title, broad terms, heading words, identifiers, cabicodes]	916

175.		Zambia.mp. [mp=abstract, title, original title, broad terms, heading words, identifiers, cabicodes]	2634
176.		Zimbabwe.mp. [mp=abstract, title, original title, broad terms, heading words, identifiers, cabicodes]	2171
177.		42 or 43 or 44 or 45 or 46 or 47 or 48 or 49 or 50 or 51 or 52 or 53 or 54 or 55 or 56 or 57 or 58 or 59 or 60 or 61 or 62 or 63 or 64 or 65 or 66 or 67 or 68 or 69 or 70 or 71 or 72 or 73 or 74 or 75 or 76 or 77 or 78 or 79 or 80 or 81 or 82 or 83 or 84 or 85 or 86 or 87 or 88 or 89 or 90 or 91 or 92 or 93 or 94 or 95 or 96 or 97 or 98 or 99 or 100 or 101 or 102 or 103 or 104 or 105 or 106 or 107 or 108 or 109 or 110 or 111 or 112 or 113 or 114 or 115 or 116 or 117 or 118 or 119 or 120 or 121 or 122 or 123 or 124 or 125 or 126 or 127 or 128 or 129 or 130 or 131 or 132 or 133 or 134 or 135 or 136 or 137 or 138 or 139 or 140 or 141 or 142 or 143 or 144 or 145 or 146 or 147 or 148 or 149 or 150 or 151 or 152 or 153 or 154 or 155 or 156 or 157 or 158 or 159 or 160 or 161 or 162 or 163 or 164 or 165 or 166 or 167 or 168 or 169 or 170 or 171 or 172 or 173 or 174 or 175 or 176	569993
178.		developing countr*.mp. [mp=abstract, title, original title, broad terms, heading words, identifiers, cabicodes]	567746
179.		low-and middle income countr*.mp. [mp=abstract, title, original title, broad terms, heading words, identifiers, cabicodes]	5367
180.		LMIC*.mp. [mp=abstract, title, original title, broad terms, heading words, identifiers, cabicodes]	1389
181.		low income countr*.mp. [mp=abstract, title, original title, broad terms, heading words, identifiers, cabicodes]	2855
182.		middle income countr*.mp. [mp=abstract, title, original title, broad terms, heading words, identifiers, cabicodes]	6787
183.		lower middle income countr*.mp. [mp=abstract, title, original title, broad terms, heading words, identifiers, cabicodes]	285
184.		upper middle income countr*.mp. [mp=abstract, title, original title, broad terms, heading words, identifiers, cabicodes]	143
185.		178 or 179 or 180 or 181 or 182 or 183 or 184	570617
186.		177 or 185	623022
187.		18 and 32 and 41 and 186	130

Final search output

130

Search Summary-4

Name of the database

Cochrane (Advanced)

Date of search

30 September, 2021

Timeline

Not applicable

Search Language

Line #	Theme	Search Language	Output #
1	Hypoxaemia	hypoxemia	3,210
2		hypoxaemia	3,210
3		anoxia	264
4		hypoxia	7,110
5		oximetry	4,239
6		oximeter	1,611
7		oximetre	10
8		oxymetry	212
9		oxymeter	126
10		oxymetre	1
11		blood oxygen saturation	7,914
12		arterial oxygen saturation	5.098
13		blood gas monitoring	1,513
14		blood gas analysis	5,573
15		analysis blood gas	5,573
16		SpO2	4,599
17		SPO2	4,599
18		1 or 2 or 3 or 4 or 5 or 6 or 7 or 8 or 9 or 10 or 11 or 12 or 13 or 14 or 15 or 16 or 17<br>hypoxemia OR hypoxaemia OR anoxia OR hypoxia OR oximetry OR oximeter OR oximetre OR oxymetry OR oxymeter OR oxymetre OR (blood oxygen saturation) OR (arterial oxygen saturation) OR (blood gas monitoring) OR (blood gas analysis) OR (analysis blood gas) OR SpO2 OR SPO2	27,530
19	Pneumonia	pneumonia	18,602
20		severe pneumonia	4,571
21		severe disease	49,561
22		very severe disease	8,482

23		bronchiolitis	1,648
24		acute respiratory infection	5,442
25		ARI	6,581
26		acute respiratory infections	4,112
27		acute respiratory tract infection	2,953
28		ARTI	116
29		acute respiratory tract infections	2,394
30		respiratory illness*	6,484
31		respiratory illnesses*	997
32		19 or 20 or 21 or 22 or 23 or 24 or 25 or 26 or 27 or 28 or 29 or 30 or 31<br>pneumonia OR (severe pneumonia) OR (severe disease) OR (very severe disease) OR bronchiolitis OR (acute respiratory infection) OR ari OR (acute respiratory infections) OR (acute respiratory tract infection) OR arti OR (acute respiratory tract infections) OR (respiratory illness*) OR (respiratory illnesses*)	77,649
33	children under 5 years of age	child	176,881
34		children	176,879
35		infant	53,917
36		infants	35,995
37		pediatric	55,900
38		pediatrics	28,695
39		paediatric	55,893
40		paediatrics	28,695
41		33 or 34 or 35 or 36 or 37 or 38 or 39 or 40<br>Child* OR children* OR infant* OR infants* OR pediatric* OR pediatrics* OR paediatric* OR paediatrics*	226,372
42	**low and middle income countries**	Albania OR Algeria OR Angola OR American Samoa OR Armenia OR Azerbaijan OR Bangladesh OR Benin OR Belarus OR Belize OR Bhutan OR Bolivia OR “Bosnia and Herzegovina” OR Botswana OR Brazil OR Bulgaria OR "Burkina Faso" OR Burundi OR Cambodia OR Cameroon OR "Cape Verde" OR “Central African Republic” OR Chad OR China OR Colombia OR Comoros OR ”Congo, Dem. Rep” OR “Congo, Rep.” OR "Costa Rica" OR "Cote d'Ivoire" OR Cuba OR Djibouti OR Dominica OR “Dominican Republic” OR “Ecuador” OR " Egypt, Arab Rep." OR "El Salvador" OR Eritrea OR Ethiopia OR Ethiopia OR Fiji OR Gabon OR “Gambia, The” OR Georgia OR Ghana OR Grenada OR Guatemala OR Guinea OR Guyana OR “Guinea-Bissau “ OR Haiti OR Honduras OR India OR Maldives OR Indonesia OR “Iran, Islamic Rep.” OR Iraq OR Jamaica OR Jordan OR Kazakhstan OR Kenya OR Kiribati	184,808

		OR “Korea, Dem. People's Rep.” OR Kosovo OR “Kyrgyz Republic” OR “Lao PDR” OR Lebanon OR Lesotho OR Liberia OR Libya OR “Macedonia, FYR” OR Madagascar OR Malaysia OR Malawi OR Mali OR “Marshall Islands” OR Mauritania OR Mauritius OR Mexico OR “Micronesia, Fed. Sts.” OR Moldova OR Mongolia OR Montenegro OR Morocco OR Mozambique OR Myanmar OR Namibia OR Nepal OR Nauru OR Nicaragua OR Niger OR Nigeria OR Pakistan OR “Papua New Guinea” OR Paraguay OR Peru OR Philippines OR Romania OR “Russian Federation” OR Rwanda OR Samoa OR " São Tomé and Principe" OR Senegal OR Serbia OR " Sierra Leone" OR Somalia OR Grenadines OR “Samoan Islands” OR " South Africa" OR “St. Lucia” OR “St. Vincent and the Grenadines” OR Sudan OR Suriname OR Swaziland OR " Syrian Arab Republic" OR "Sri Lanka" OR Tajikistan OR Tanzania OR Thailand OR Togo OR Tonga OR Tunisia OR Turkey OR Turkmenistan OR Tuvalu OR “Timor-Leste” OR Uganda OR Ukraine OR Uzbekistan OR Vanuatu OR Venezuela, RB OR Vietnam OR “West Bank and Gaza” OR “Yemen, Rep.” OR Zambia OR Zimbabwe OR (developing countr*) OR (low-and middle income countr*) OR (LMIC*) OR (low income countr*) OR (middle income countr*) OR (lower middle income countr*) OR (upper middle income countr*)	
43		18 AND 32 AND 41 AND 42	153

Final search output: 153

Search Summary 5

Name of the database

Clinicaltrails.gov

Date of search

30 September, 2021

Timeline

Not applicable

Search Language

Line #	Theme	hypoxemia	Output #
1	Hypoxaemia	hypoxemia	884
2		hypoxaemia	884
3		anoxia	35
4		hypoxia	2383
5		oximetry	1978
6		oximeter	960
7		oximetre	0
8		oxymetry	1978
9		oxymeter	41
10		oxymetre	2
11		blood oxygen saturation	2514
12		arterial oxygen saturation	4182
13		blood gas monitoring	198
14		blood gas analysis	1433
15		analysis blood gas	1413
16		spo2	1883
17		spo2	1883
18		1 or 2 or 3 or 4 or 5 or 6 or 7 or 8 or 9 or 10 or 11 or 12 or 13 or 14 or 15 or 16 or 17<br>hypoxemia OR hypoxaemia OR anoxia OR hypoxia OR oximetry OR oximeter OR oximetre OR oxymetry OR oxymeter OR oxymetre OR blood oxygen saturation OR arterial oxygen saturation OR blood gas monitoring OR blood gas analysis OR analysis blood gas OR spo2 OR spo2	9238
19	Pneumonia	pneumonia	10129
20		“severe pneumonia”	1707
21		“severe disease”	40422
22		“very severe disease”	6447
23		bronchiolitis	447
24		acute respiratory infection	2159
25		ari	406
26		acute respiratory infections	2159

27		acute respiratory tract infection	2335
28		arti	63
29		acute respiratory tract infections	2335
30		respiratory illness	1233
31		respiratory illnesses	1233
32		19 or 20 or 21 or 22 or 23 or 24 or 25 or 26 or 27 or 28 or 29 or 30 or 31<br>pneumonia OR "severe pneumonia" OR "severe disease" OR "very severe disease" OR bronchiolitis OR acute respiratory infection OR ari OR acute respiratory infections OR acute respiratory tract infection OR arti OR acute respiratory tract infections OR respiratory illness OR respiratory illnesses	9,179
33	children under 5 years of age	child	85169
34		children	85169
35		infant	14246
36		infants	14246
37		pediatric	15308
38		pediatrics	6618
39		paediatric	6609
40		paediatrics	6618
41		33 or 34 or 35 or 36 or 37 or 38 or 39 or 40<br>child OR children OR infant OR infants OR pediatric OR pediatrics OR paediatric OR paediatrics	89244
42		developing country OR developing countries OR low-and middle income country OR low-and middle income countries OR LMIC OR LMICs OR low income country OR low income countries OR middle income country OR middle income countries OR lower middle income country OR lower middle income countries OR upper middle income country OR upper middle income countries OR Afghanistan OR Albania OR Albania OR American Samoa OR Angola OR Armenia OR Azerbaijan OR Bangladesh OR Belarus OR Belize OR Benin OR Bhutan OR Bolivia OR Bosnia and Herzegovina OR Botswana OR Brazil OR Bulgaria OR Burkina Faso OR Burundi OR Cabo Verde OR Cambodia OR Cameroon OR Central African Republic OR Chad OR China OR Colombia OR Comoros OR DRC OR Congo OR Costa Rica OR Ivory Coast OR Cuba OR Djibouti OR Dominica OR Dominican Republic OR Ecuador OR Egypt OR El Salvador OR Guinea OR Eritrea OR Ethiopia OR Fiji OR Gabon OR Gambia OR Georgia OR Ghana OR	154,847

		Grenada OR Guatemala OR Guinea OR Guinea-Bissau OR Guyana OR Haiti OR Honduras OR India OR Indonesia OR Iran OR Iraq OR Jamaica OR Jordan OR Kazakhstan OR Kenya OR Kiribati OR Korea OR Kosovo OR Kyrgyz OR Lao OR Lebanon OR Lesotho OR Liberia OR Libya OR Macedonia, FYR OR Madagascar OR Malawi OR Malaysia OR Maldives OR Mali OR Marshall Islands OR Mauritania OR Mauritius OR Mexico OR Micronesia OR Moldova OR Mongolia OR Montenegro OR Morocco OR Mozambique OR Myanmar OR Namibia OR Nauru OR Nepal OR Nicaragua OR Niger OR Nigeria OR Pakistan OR Papua New Guinea OR Paraguay OR Peru OR Philippines OR Romania OR Russia OR Rwanda OR Samoa OR Timor OR Timor-Liste OR Senegal OR Serbia OR Sierra Leone OR Solomon Islands OR Somalia OR South Africa OR Sudan OR Sri Lanka OR Saint Lucia OR St. Vincent and the Grenadines OR Sudan OR Suriname OR Swaziland OR Syria OR Tajikistan OR Tanzania OR Thailand OR Timor-Leste OR Togo OR Tonga OR Tunisia OR Turkey OR Turkmenistan OR Tuvalu OR Uganda OR Ukraine OR Uzbekistan OR Vanuatu OR Venezuela OR Vietnam OR Gaza OR Yemen OR Zambia OR Zimbabwe	
43		18 AND 32 AND 41 AND 42<br>(hypoxemia OR hypoxaemia OR anoxia OR hypoxia OR oximetry OR oximeter OR oximetre OR oxymetry OR oxymeter OR oxymetre OR blood oxygen saturation OR arterial oxygen saturation OR blood gas monitoring OR blood gas analysis OR analysis blood gas OR spo2 OR spo2) AND (pneumonia OR "severe pneumonia" OR "severe disease" OR "very severe disease" OR bronchiolitis OR acute respiratory infection OR ari OR acute respiratory infections OR acute respiratory tract infection OR arti OR acute respiratory tract infections OR respiratory illness OR respiratory illnesses) AND (child OR children OR infant OR infants OR pediatric OR pediatrics OR paediatric OR paediatrics) AND (developing country OR Developing countries OR low-and middle income country OR low-and middle income countries OR LMIC OR LMICs OR low income country OR low income countries OR middle income country OR middle income countries OR lower middle income country OR lower middle income countries	85

		OR upper middle income country OR upper middle income countries OR Afghanistan OR Albania OR Albania OR “American Samoa” OR Angola OR Armenia OR Azerbaijan OR Bangladesh OR Belarus OR Belize OR Benin OR Bhutan OR Bolivia OR Bosnia and Herzegovina OR Botswana OR Brazil OR Bulgaria OR Burkina Faso OR Burundi OR Cabo Verde OR Cambodia OR Cameroon OR Central African Republic OR Chad OR China OR Colombia OR Comoros OR DRC OR Congo OR Costa Rica OR Ivory Coast OR Cuba OR Djibouti OR Dominica OR Dominican Republic OR Ecuador OR Egypt OR El Salvador OR Guinea OR Eritrea OR Ethiopia OR Fiji OR Gabon OR Gambia OR Georgia OR Ghana OR Grenada OR Guatemala OR Guinea OR Guinea-Bissau OR Guyana OR Haiti OR Honduras OR India OR Indonesia OR Iran OR Iraq OR Jamaica OR Jordan OR Kazakhstan OR Kenya OR Kiribati OR Korea OR Kosovo OR Kyrgyz OR Lao OR Lebanon OR Lesotho OR Liberia OR Libya OR Macedonia, FYR OR Madagascar OR Malawi OR Malaysia OR Maldives OR Mali OR Marshall Islands OR Mauritania OR Mauritius OR Mexico OR Micronesia OR Moldova OR Mongolia OR Montenegro OR Morocco OR Mozambique OR Myanmar OR Namibia OR Nauru OR Nepal OR Nicaragua OR Niger OR Nigeria OR Pakistan OR Papua New Guinea OR Paraguay OR Peru OR Philippines OR Romania OR Russia OR Rwanda OR Samoa OR Timor OR Timor-Liste OR Senegal OR Serbia OR Sierra Leone OR Solomon Islands OR Somalia OR South Africa OR Sudan OR Sri Lanka OR Saint Lucia OR St. Vincent and the Grenadines OR Sudan OR Suriname OR Swaziland OR Syria OR Tajikistan OR Tanzania OR Thailand OR Timor-Leste OR Togo OR Tonga OR Tunisia OR Turkey OR Turkmenistan OR Tuvalu OR Uganda OR Ukraine OR Uzbekistan OR Vanuatu OR Venezuela OR Vietnam OR Gaza OR Yemen OR Zambia OR Zimbabwe)	

Final search output: 85

Search Summary 6

Name of the database

SCOPUS (Advanced)

Date of search

29 September, 2021

Timeline

Not applicable

Search Language

Line #	Theme	Search Language	Output #
1	Hypoxaemia	hypoxemia	77,572
2		hypoxaemia	18,843
3		anoxia	126,145
4		hypoxia	703,759
5		oximetry	58,162
6		oximeter	15,210
7		oximetre	1
8		oxymetry	2,156
9		oxymeter	400
10		oxymetre	93
11		“blood oxygen saturation”	5,699
12		“arterial oxygen saturation”	16,179
13		“blood gas monitoring”	3,389
14		“blood gas analysis”	38,710
15		“analysis blood gas”	23
16		SpO2	9,072
17		SPO2	9,072
18		1 or 2 or 3 or 4 or 5 or 6 or 7 or 8 or 9 or 10 or 11 or 12 or 13 or 14 or 15 or 16 or 17<br>(hypoxemia) OR (hypoxaemia) OR (anoxia) OR (hypoxia) OR (oximetry) OR (oximeter) OR (oximetre) OR (oxymetr y) OR (oxymeter) OR (oxymetre) OR ("blood oxygen saturation") OR ("arterial oxygen saturation") OR ("blood gas monitoring") OR ("blood gas analysis") OR ("analysis blood gas") OR (spo2) OR (spo2)	910,266
19	Pneumonia	pneumonia	608,133
20		“severe pneumonia”	15,974
21		“severe disease”	43,407
22		“very severe disease”	302

23		bronchiolitis	51,454
24		“acute respiratory infection”	28,443
25		ARI	176,104
26		“acute respiratory infections”	28,443
27		“acute respiratory tract infection”	13,820
28		ARTI	30,077
29		“acute respiratory tract infections”	13,820
30		“respiratory illness*”	38,309
31		“respiratory illnesses*”	9,267
32		19 or 20 or 21 or 22 or 23 or 24 or 25 or 26 or 27 or 28 or 29 or 30 or 31<br>(pneumonia) OR ("severe pneumonia") OR ("severe disease") OR ("very severe disease") OR (bronchiolitis) OR ("acute respiratory infection") OR (ari) OR ("acute respiratory infections") OR ("acute respiratory infections") OR ("acute respiratory tract infection") OR (arti) OR ("acute respiratory tract infections") OR ("respiratory illness*") OR ("respiratory illnesses*")	910,802
33	children under 5 years of age	Child*	7,610,183
34		Children*	5,027,116
35		Infant*	2,626,853
36		Infants*	1,074,853
37		Pediatric*	3,941,378
38		Pediatrics*	2,339,629
39		Paediatric*	985,834
40		Paediatrics*	437,141
41		33 or 34 or 35 or 36 or 37 or 38 or 39 or 40<br>(child*) OR (children*) OR (infant*) OR (infants*) OR (pediatric*) OR (pediatrics*) OR (paediatric*) OR (paediatrics*)	9,337,152
42	low and middle income countries	Albania OR Algeria OR Angola OR American Samoa OR Armenia OR Azerbaijan OR Bangladesh OR Benin OR Belarus OR Belize OR Bhutan OR Bolivia OR “Bosnia and Herzegovina” OR Botswana OR Brazil OR Bulgaria OR "Burkina Faso" OR Burundi OR Cambodia OR Cameroon OR "Cape Verde" OR “Central African Republic” OR Chad OR China OR Colombia OR Comoros OR ”Congo, Dem. Rep” OR “Congo, Rep.” OR "Costa Rica" OR "Cote d'Ivoire" OR Cuba OR Djibouti OR Dominica OR “Dominican Republic” OR “Ecuador” OR " Egypt, Arab Rep." OR "El Salvador" OR Eritrea OR Ethiopia OR Ethiopia OR Fiji OR Gabon OR “Gambia, The” OR Georgia OR Ghana OR Grenada OR Guatemala OR Guinea OR Guyana OR “Guinea-Bissau “ OR Haiti OR Honduras OR India OR Maldives OR Indonesia OR “Iran, Islamic	758,189

		Rep." OR Iraq OR Jamaica OR Jordan OR Kazakhstan OR Kenya OR Kiribati OR "Korea, Dem. People's Rep." OR Kosovo OR "Kyrgyz Republic" OR "Lao PDR" OR Lebanon OR Lesotho OR Liberia OR Libya OR "Macedonia, FYR" OR Madagascar OR Malaysia OR Malawi OR Mali OR "Marshall Islands" OR Mauritania OR Mauritius OR Mexico OR "Micronesia, Fed. Sts." OR Moldova OR Mongolia OR Montenegro OR Morocco OR Mozambique OR Myanmar OR Namibia OR Nepal OR Nauru OR Nicaragua OR Niger OR Nigeria OR Pakistan OR "Papua New Guinea" OR Paraguay OR Peru OR Philippines OR Romania OR "Russian Federation" OR Rwanda OR Samoa OR " São Tomé and Principe" OR Senegal OR Serbia OR " Sierra Leone" OR Somalia OR Grenadines OR "Samoan Islands" OR " South Africa" OR "St. Lucia" OR "St. Vincent and the Grenadines" OR Sudan OR Suriname OR Swaziland OR " Syrian Arab Republic" OR "Sri Lanka" OR Tajikistan OR Tanzania OR Thailand OR Togo OR Tonga OR Tunisia OR Turkey OR Turkmenistan OR Tuvalu OR "Timor-Leste" OR Uganda OR Ukraine OR Uzbekistan OR Vanuatu OR Venezuela, RB OR Vietnam OR "West Bank and Gaza" OR "Yemen, Rep." OR Zambia OR Zimbabwe OR (developing countr*) OR ("low-and middle income countr*") OR (LMIC*) OR ("low income countr*") OR ("middle income countr*") OR ("lower middle income countr*") OR ("upper middle income countr*")	
43		18 AND 32 AND 41 AND 42<br>(with filter<br>NURSING, MEDICINE-Limit to<br>HUMAN, HUMANS- Limit to<br>JOURNAL- Limit to)	665

Final search output : 665

Search Summary 7

Name of the database

CINHAL

Date of search

30 September, 2021

Timeline

Not applicable

Search Language

Line #	Theme	Search Language	Output #
1	Hypoxaemia	hypoxemia	3,895
2		hypoxaemia	678
3		anoxia	7,135
4		hypoxia	14,894
5		oximetry	6,768
6		oximeter	3,134
7		oximetre	0
8		oxymetry	47
9		oxymeter	19
10		oxymetre	4
11		blood oxygen saturation	1,351
12		arterial oxygen saturation	990
13		blood gas monitoring	645
14		blood gas analysis	5,354
15		analysis blood gas	1,293
16		SpO2	1,718
17		SPO2	1,718
18		1 or 2 or 3 or 4 or 5 or 6 or 7 or 8 or 9 or 10 or 11 or 12 or 13 or 14 or 15 or 16 or 17<br>hypoxemia OR hypoxaemia OR anoxia OR hypoxia OR oximetry OR oximeter OR oximetre OR oxymetry OR oxymeter OR oxymetre OR (blood oxygen saturation) OR (arterial oxygen saturation) OR (blood gas monitoring) OR (blood gas analysis) OR (analysis blood gas) OR SpO2 OR SPO2	33,731
19	Pneumonia	pneumonia	46,202
20		severe pneumonia	786
21		severe disease	3,915
22		very severe disease	45

23		bronchiolitis	3,219
24		acute respiratory infection	1,114
25		ARI	4,183
26		acute respiratory infections	1,114
27		acute respiratory tract infection	386
28		ARTI	6,416
29		acute respiratory tract infections	386
30		respiratory illness*	4,802
31		respiratory illnesses*	627
32		19 or 20 or 21 or 22 or 23 or 24 or 25 or 26 or 27 or 28 or 29 or 30 or 31<br>pneumonia OR (severe pneumonia) OR (severe disease) OR (very severe disease) OR bronchiolitis OR (acute respiratory infection) OR ari OR (acute respiratory infections) OR (acute respiratory tract infection) OR arti OR (acute respiratory tract infections) OR (respiratory illness*) OR (respiratory illnesses*)	67,715
33	children under 5 years of age	Child*	848,219
34		Children*	401,406
35		Infant*	314,473
36		Infants*	76,280
37		Pediatric*	182,754
38		Pediatrics*	33,177
39		Paediatric*	27,335
40		Paediatrics*	12,813
41		33 or 34 or 35 or 36 or 37 or 38 or 39 or 40<br>Child* OR children* OR infant* OR infants* OR pediatric* OR pediatrics* OR paediatric* OR paediatrics*	998,872
42	**low and middle income countries**	Albania OR Algeria OR Angola OR American Samoa OR Armenia OR Azerbaijan OR Bangladesh OR Benin OR Belarus OR Belize OR Bhutan OR Bolivia OR “Bosnia and Herzegovina” OR Botswana OR Brazil OR Bulgaria OR "Burkina Faso" OR Burundi OR Cambodia OR Cameroon OR "Cape Verde" OR “Central African Republic” OR Chad OR China OR Colombia OR Comoros OR ”Congo, Dem. Rep” OR “Congo, Rep.” OR "Costa Rica" OR "Cote d'Ivoire" OR Cuba OR Djibouti OR Dominica OR “Dominican Republic” OR “Ecuador” OR " Egypt, Arab Rep." OR "El Salvador" OR Eritrea OR Ethiopia OR Ethiopia OR Fiji OR Gabon OR “Gambia, The” OR Georgia OR Ghana OR Grenada OR Guatemala OR Guinea OR Guyana OR “Guinea-Bissau “ OR Haiti OR Honduras OR India OR Maldives OR Indonesia OR “Iran, Islamic Rep.” OR Iraq OR Jamaica OR Jordan OR Kazakhstan OR Kenya OR Kiribati OR “Korea, Dem. People's Rep.” OR Kosovo OR “Kyrgyz Republic” OR “Lao PDR” OR Lebanon OR	438,431

		Lesotho OR Liberia OR Libya OR “Macedonia, FYR” OR Madagascar OR Malaysia OR Malawi OR Mali OR “Marshall Islands” OR Mauritania OR Mauritius OR Mexico OR “Micronesia, Fed. Sts.” OR Moldova OR Mongolia OR Montenegro OR Morocco OR Mozambique OR Myanmar OR Namibia OR Nepal OR Nauru OR Nicaragua OR Niger OR Nigeria OR Pakistan OR “Papua New Guinea” OR Paraguay OR Peru OR Philippines OR Romania OR “Russian Federation” OR Rwanda OR Samoa OR " São Tomé and Principe" OR Senegal OR Serbia OR " Sierra Leone" OR Somalia OR Grenadines OR “Samoan Islands” OR " South Africa" OR “St. Lucia” OR “St. Vincent and the Grenadines” OR Sudan OR Suriname OR Swaziland OR " Syrian Arab Republic" OR "Sri Lanka" OR Tajikistan OR Tanzania OR Thailand OR Togo OR Tonga OR Tunisia OR Turkey OR Turkmenistan OR Tuvalu OR “Timor-Leste” OR Uganda OR Ukraine OR Uzbekistan OR Vanuatu OR Venezuela, RB OR Vietnam OR “West Bank and Gaza” OR “Yemen, Rep.” OR Zambia OR Zimbabwe OR (developing countr*) OR (low-and middle income countr*) OR (LMIC*) OR (low income countr*) OR (middle income countr*) OR (lower middle income countr*) OR (upper middle income countr*)	
43		18 AND 32 AND 41 AND 42	66

Final search output : 66

Search Summary 8

Name of the database

PubMed (Advanced)

Date of search

29 September, 2021

Timeline

Not applicable

Search Language

Line #	Theme	Search Language	Output #
1	Hypoxaemia	hypoxemia	181,951
2		hypoxaemia	181,951
3		anoxia	174,123
4		hypoxia	170,128
5		oximetry	20,991
6		oximeter	4,324
7		oximetre	0
8		oxymetry	21,276
9		oxymeter	157
10		oxymetre	11
11		blood oxygen saturation	37,918
12		arterial oxygen saturation	17,275
13		blood gas monitoring	13,164
14		blood gas analysis	60,188
15		analysis blood gas	60,188
16		SpO2	7,221
17		SPO2	7,221
18		Search ((((((((((((((((hypoxemia) OR hypoxaemia) OR anoxia) OR hypoxia) OR oximetry) OR oximeter) OR oximetre) OR oxymetry) OR oxymeter) OR oxymetre) OR blood oxygen saturation) OR arterial oxygen saturation) OR blood gas monitoring) OR blood gas analysis) OR analysis blood gas) OR SpO2) OR SPO2	263,664
19	Pneumonia	pneumonia	335,840
20		severe pneumonia	64,569
21		severe disease	763,868
22		very severe disease	763,868
23		bronchiolitis	14,288

24		acute respiratory infection	86,480
25		ARI	7,231
26		acute respiratory infections	80,388
27		acute respiratory tract infection	73,381
28		ARTI	2,155
29		acute respiratory tract infections	71,954
30		respiratory illness*	47,137
31		respiratory illnesses*	6,388
32		Search (((((((((((pneumonia) OR severe pneumonia) OR severe disease) OR very severe disease) OR bronchiolitis) OR acute respiratory infection) OR ARI) OR acute respiratory infections) OR acute respiratory tract infection) OR ARTI) OR acute respiratory tract infections) OR respiratory illness*) OR respiratory illnesses*	1,122,148
33	children under 5 years of age	Child*	2,960,040
34		Children*	1,515,039
35		Infant*	1,351,126
36		Infants*	282,393
37		Pediatric*	988,382
38		Pediatrics*	524,507
39		Paediatric*	187,928
40		Paediatrics*	79,099
41		Search (((((((Child*) OR Children*) OR Infant*) OR Infants*) OR Pediatric*) OR Pediatrics*) OR Paediatric*) OR Paediatrics*	3,722,177
42	**low and middle income countries**	Search (Albania OR Algeria OR Angola OR American Samoa OR Armenia OR Azerbaijan OR Bangladesh OR Benin OR Belarus OR Belize OR Bhutan OR Bolivia OR “Bosnia and Herzegovina” OR Botswana OR Brazil OR Bulgaria OR "Burkina Faso" OR Burundi OR Cambodia OR Cameroon OR "Cape Verde" OR “Central African Republic” OR Chad OR China OR Colombia OR Comoros OR ”Congo, Dem. Rep” OR “Congo, Rep.” OR "Costa Rica" OR "Cote d'Ivoire" OR Cuba OR Djibouti OR Dominica OR “Dominican Republic” OR “Ecuador” OR " Egypt, Arab Rep." OR "El Salvador" OR Eritrea OR Ethiopia OR Ethiopia OR Fiji OR Gabon OR “Gambia, The” OR Georgia OR Ghana OR Grenada OR Guatemala OR Guinea OR Guyana OR “Guinea-Bissau “ OR Haiti OR Honduras OR India OR Maldives OR Indonesia OR “Iran, Islamic Rep.” OR Iraq OR Jamaica OR Jordan OR Kazakhstan OR Kenya OR Kiribati OR “Korea, Dem. People's Rep.” OR Kosovo OR “Kyrgyz Republic” OR “Lao PDR” OR Lebanon OR Lesotho OR Liberia OR Libya	5,178,516

		OR “Macedonia, FYR” OR Madagascar OR Malaysia OR Malawi OR Mali OR “Marshall Islands” OR Mauritania OR Mauritius OR Mexico OR “Micronesia, Fed. Sts.” OR Moldova OR Mongolia OR Montenegro OR Morocco OR Mozambique OR Myanmar OR Namibia OR Nepal OR Nauru OR Nicaragua OR Niger OR Nigeria OR Pakistan OR “Papua New Guinea” OR Paraguay OR Peru OR Philippines OR Romania OR “Russian Federation” OR Rwanda OR Samoa OR " São Tomé and Principe" OR Senegal OR Serbia OR " Sierra Leone" OR Somalia OR Grenadines OR “Samoan Islands” OR " South Africa" OR “St. Lucia” OR “St. Vincent and the Grenadines” OR Sudan OR Suriname OR Swaziland OR " Syrian Arab Republic" OR "Sri Lanka" OR Tajikistan OR Tanzania OR Thailand OR Togo OR Tonga OR Tunisia OR Turkey OR Turkmenistan OR Tuvalu OR “Timor-Leste” OR Uganda OR Ukraine OR Uzbekistan OR Vanuatu OR Venezuela, RB OR Vietnam OR “West Bank and Gaza” OR “Yemen, Rep.” OR Zambia OR Zimbabwe OR (developing countr*) OR (low-and middle income countr*) OR (LMIC*) OR (low income countr*) OR (middle income countr*) OR (lower middle income countr*) OR (upper middle income countr*))	
43		18 AND 32 AND 41 AND 42 ((((((((((((((((((hypoxemia) OR hypoxaemia) OR anoxia) OR hypoxia) OR oximetry) OR oximeter) OR oximetry) OR oxymetry) OR oximeter) OR oxymetry) OR blood oxygen saturation) OR arterial oxygen saturation) OR blood gas monitoring) OR blood gas analysis) OR analysis blood gas) OR SpO2) OR SPO2)) AND ((((((((((((pneumonia) OR severe pneumonia) OR severe disease) OR very severe disease) OR bronchiolitis) OR acute respiratory infection) OR ARI) OR acute respiratory infections) OR acute respiratory tract infection) OR arti) OR acute respiratory tract infections) OR respiratory illness*) OR respiratory illnesses*)) AND ((((((((Child*) OR Children*) OR Infant*) OR Infants*) OR Pediatric*) OR Pediatrics*) OR Paediatric*) OR Paediatrics*)) AND ((albania OR algeria OR angola OR american samoa OR armenia OR azerbaijan OR bangladesh OR benin OR belarus OR belize OR bhutan OR bolivia OR "Bosnia and Herzegovina" OR botswana OR brazil OR bulgaria OR "Burkina Faso" OR burundi OR cambodia OR cameroon OR "Cape Verde" OR "Central African Republic" OR chad OR china OR colombia OR comoros OR "Congo,	907

		Dem. Rep" OR "Congo, Rep." OR "Costa Rica" OR "Cote d'Ivoire" OR cuba OR djibouti OR dominica OR "Dominican Republic" OR "Ecuador" OR "Egypt, Arab Rep." OR "El Salvador" OR eritrea OR ethiopia OR ethiopia OR fiji OR gabon OR "Gambia, The" OR georgia OR ghana OR grenada OR guatemala OR guinea OR guyana OR "Guinea-Bissau " OR haiti OR honduras OR india OR maldives OR indonesia OR "Iran, Islamic Rep." OR iraq OR jamaica OR jordan OR kazakhstan OR kenya OR kiribati OR "Korea, Dem. People's Rep." OR kosovo OR "Kyrgyz Republic" OR "Lao PDR" OR lebanon OR lesotho OR liberia OR libya OR "Macedonia, FYR" OR madagascar OR malaysia OR malawi OR mali OR "Marshall Islands" OR mauritania OR mauritius OR mexico OR "Micronesia, Fed. Sts." OR moldova OR mongolia OR montenegro OR morocco OR mozambique OR myanmar OR namibia OR nepal OR nauru OR nicaragua OR niger OR nigeria OR pakistan OR "Papua New Guinea" OR paraguay OR peru OR philippines OR romania OR "Russian Federation" OR rwanda OR samoa OR "Sao Tome and Principe" OR senegal OR serbia OR "Sierra Leone" OR somalia OR grenadines OR "Samoan Islands" OR "South Africa" OR "St. Lucia" OR "St. Vincent and the Grenadines" OR sudan OR suriname OR swaziland OR "Syrian Arab Republic" OR "Sri Lanka" OR tajikistan OR tanzania OR thailand OR togo OR tonga OR tunisia OR turkey OR turkmenistan OR tuvalu OR "Timor-Leste" OR uganda OR ukraine OR uzbekistan OR vanuatu OR venezuela, rb OR vietnam OR "West Bank and Gaza" OR "Yemen, Rep." OR zambia OR zimbabwe OR (developing countr*) OR (low-and middle income countr*) OR (LMIC*) OR (low income countr*) OR (middle income countr*) OR (lower middle income countr*) OR (upper middle income countr*)))	

<u>Final search output: </u>907

Search Summary 9

Name of the database

Global Index Medicus (WHO)

- African Index Medicus (AFRO)
- literature of Latin America and the Caribbean (AMRO/Pan American Health Organizaton)
- The Index Medicus for the Estern Mediterranean Region (IMEMR/ EMRO)
- The Index Medicus for South- East Asia Region (IMSEAR/ SEARO)
- Western Pacific Region Index Medicus (WPRIM/ WPRO)

Date of search

30 September, 2021

Timeline

Not applicable

Search Language

Line #	Theme	Search Language	Output #
1	Hypoxaemia	(tw:(hypoxemia)) AND (instance:"ghl")	3.987
2		tw:(hypoxaemia) AND (instance:"ghl")	101
3		tw:(anoxia) AND (instance:"ghl")	4,848
4		tw:(hypoxia) AND (instance:"ghl")	9.655
5		tw:(oximetry) AND (instance:"ghl")	1.034
6		tw:(oximeter) AND (instance:"ghl")	297
7		oximetre	0
8		tw:(oxymetry) AND (instance:"ghl")	83
9		tw:(oxymeter) AND (instance:"ghl")	37
10		oxymetre	0
11		tw:(blood oxygen saturation) AND (instance:"ghl")	2.152
12		tw:(arterial oxygen saturation) AND (instance:"ghl")	1.616
13		tw:(blood gas monitoring) AND (instance:"ghl")	620
14		tw:(blood gas analysis) AND (instance:"ghl")	3.715
15		tw:(analysis blood gas) AND (instance:"ghl")	3.715
16		tw:(spo2) AND (instance:"ghl")	1.095
17		tw:(spo2) AND (instance:"ghl")	1.095
18		1 or 2 or 3 or 4 or 5 or 6 or 7 or 8 or 9 or 10 or 11 or 12 or 13 or 14 or 15 or 16 or 17<br>tw:(hypoxemia OR hypoxaemia OR anoxia OR hypoxia OR oximetry OR oximeter OR oximetre OR oxymetry OR	18,269

		oxymeter OR oxymetre OR (blood oxygen saturation) OR (arterial oxygen saturation) OR (blood gas monitoring) OR (blood gas analysis) OR (analysis blood gas) OR spo2 OR spo2) AND (instance:"ghl")	
19	Pneumonia	tw:(pneumonia) AND (instance:"ghl")	22.597
20		tw:(severe pneumonia) AND (instance:"ghl")	4.682
21		tw:(severe disease) AND (instance:"ghl")	41.648
22		tw:(very severe disease) AND (instance:"ghl")	2.533
23		tw:(bronchiolitis) AND (instance:"ghl")	1.540
24		tw:(acute respiratory infection) AND (instance:"ghl")	6.270
25		tw:(ari) AND (instance:"ghl")	703
26		tw:(acute respiratory infections) AND (instance:"ghl")	5.168
27		tw:(acute respiratory tract infection) AND (instance:"ghl")	2.266
28		tw:(arti) AND (instance:"ghl")	140
29		tw:(acute respiratory tract infections) AND (instance:"ghl")	2.084
30		tw:(respiratory illness*) AND (instance:"ghl")	3.477
31		tw:(respiratory illnesses*) AND (instance:"ghl")	1.398
32		19 or 20 or 21 or 22 or 23 or 24 or 25 or 26 or 27 or 28 or 29 or 30 or 31<br>tw:(pneumonia OR (severe pneumonia) OR (severe disease) OR (very severe disease) OR bronchiolitis OR (acute respiratory infection) OR ari OR (acute respiratory infections) OR (acute respiratory tract infection) OR arti OR (acute respiratory tract infections) OR (respiratory illness*) OR (respiratory illnesses*)) AND (instance:"ghl")	68.111
33	children under 5 years of age	tw:(child*) AND (instance:"ghl")	253.850
34		tw:(children*) AND (instance:"ghl")	212.838
35		tw:(infant*) AND (instance:"ghl")	169.620
36		tw:(infants*) AND (instance:"ghl")	92.959
37		tw:(pediatric*) AND (instance:"ghl")	56.773
38		tw:(pediatrics*) AND (instance:"ghl")	13.240
39		tw:(paediatric*) AND (instance:"ghl")	4.003
40		tw:(paediatrics*) AND (instance:"ghl")	669
41		33 or 34 or 35 or 36 or 37 or 38 or 39 or 40<br>tw:(child* OR children* OR infant* OR infants* OR pediatric* OR pediatrics* OR paediatric* OR paediatrics*) AND (instance:"ghl")	327.748
42		18 AND 32 AND 41	376

<u>Final search output: 376</u>

Search Summary 10

Name of the database

IndMed (Advanced)

Date of search

29 January 2019

Timeline

Not applicable

Search Language

Line #	Theme	Search Language	Output #
1	Hypoxaemia	hypoxemia OR hypoxaemia OR anoxia OR hypoxia OR oximetry OR oximeter OR oximetre OR oxymetry OR oxymeter OR oxymetre OR blood oxygen saturation OR arterial oxygen saturation OR blood gas monitoring OR blood gas analysis OR analysis blood gas OR SpO2 OR SPO2	685
2	Pneumonia	pneumonia OR severe pneumonia OR severe disease OR very severe disease OR bronchiolitis OR acute respiratory infection OR ARI OR acute respiratory infections OR acute respiratory tract infection OR ARTI OR acute respiratory tract infections OR respiratory illness OR respiratory illnesses	3081
3	children under 5 years of age	child OR children OR infant OR infants OR pediatric OR pediatrics OR paediatric OR paediatrics	15800
4		1 AND 2 AND 3	33

<u>Final search output :33</u>

Search Summary 11

Name of the database

Web of Science (Advanced)

Date of search

29 September, 2021

Timeline

Not applicable

Search Language

Line #	Theme	Search Language	Output #
1	Hypoxaemia	TS=(Hypoxemia)	19,563
2		TS=(hypoxaemia)	2,790
3		TS=(anoxia)	14,241
4		TS=(hypoxia)	164,284
5		TS=(oximetry)	14,422
6		TS=(oximeter)	4,006
7		TS=(oximetre)	2
8		TS=(oxymetry)	502
9		TS=(oxymeter)	155
10		TS=(oxymetre)	0
11		TS=(blood oxygen saturation)	16,069
12		TS=(arterial oxygen saturation)	10,510
13		TS=(blood gas monitoring)	6,075
14		TS=(blood gas analysis)	15,105
15		TS=(analysis blood gas)	15,105
16		TS=(SpO2)	1,910
17		TS=(SPO2)	1,910
18		#17 OR #16 OR #15 OR #14 OR #13 OR #12 OR #11 OR #10 OR #9 OR #8 OR #7 OR #6 OR #5 OR #4 OR #3 OR #2 OR #1	233,989
19	Pneumonia	TS= (Pneumonia)	144,419
20		TS=(severe pneumonia)	25,168
21		TS=(severe disease)	336,104
22		TS=(very severe disease)	21,394
23		TS=(bronchiolitis)	16,072
24		TS=(acute respiratory infection)	42,365
25		TS=(acute respiratory infections)	42,365
26		TS=(ARI)	4,984

27		TS=(acute respiratory tract infection)	11,598
28		TS=(acute respiratory tract infections)	11,598
29		TS=(ARTI)	705
30		TS=(respiratory illness*)	25,705
31		TS=(respiratory illnesses*)	5,564
32		#31 OR #30 OR #29 OR #28 OR #27 OR #26 OR #25 OR #24 OR #23 OR #22 OR #21 OR #20 OR #19	517,330
33	Children under 5 years of age	TS=(child*)	2,013,398
34		TS=(children*)	1,585,570
35		TS=(infant*)	474,019
36		TS=(infants*s)	7
37		TS=(pediatric*)	379,321
38		TS=(pediatrics*)	44,193
39		TS=(paediatric*)	75,210
40		TS=(paediatrics*)	8,380
41		#40 OR #39 OR #38 OR #37 OR #36 OR #35 OR #34 OR #33	2,472,694
42	Low and Middle Income Country	CU=(Afghanistan) OR CU=(Albania) OR CU=(Algeria) OR CU=(Angola) OR CU=(American Samoa) OR CU=(Armenia) OR CU=(Azerbaijan) OR CU=(Bangladesh) OR CU=(Benin) OR CU=(Belarus) OR CU=(Belize) OR CU=(Bhutan) OR CU=(Bolivia) OR CU=(Bosnia and Herzegovina) OR CU=(Botswana) OR CU=(Brazil) OR CU=(Bulgaria) OR CU=("Burkina Faso") OR CU=(Burundi) OR CU=(Cambodia) OR CU=(Cameroon) OR CU=("Cape Verde") OR CU=(Central African Republic) OR CU=(Chad) OR CU=(China) OR CU=(Colombia) OR CU=(Comoros) OR CU=(Congo, Dem. Rep) OR CU=(Congo, Rep.) OR CU=("Costa Rica") OR CU=("Cote d'Ivoire") OR CU=(Cuba) OR CU=(Djibouti) OR CU=(Dominica) OR CU=(Dominican Republic) OR CU=(Ecuador) OR CU=(Egypt) OR CU=("El Salvador") OR CU=(Eritrea) OR CU=(Ethiopia) OR CU=(Ethiopia) OR CU=(Fiji) OR CU=(Gabon) OR CU=(Gambia) OR CU=(Georgia) OR CU=(Ghana) OR CU=(Grenada) OR CU=(Guatemala) OR CU=(Guinea) OR CU=(Guyana) OR CU=("Guinea-Bissau") OR CU=(Haiti) OR CU=(Honduras) OR CU=(India) OR CU=(Maldives) OR CU=(Indonesia) OR CU=(Iran) OR CU=(Iraq) OR CU=(Jamaica) OR CU=(Jordan) OR CU=(Kazakhstan) OR CU=(Kenya) OR CU=(Kiribati) OR CU=(Korea) OR CU=(Kosovo) OR CU=(Kyrgyz Republic) OR CU=(Lao PDR) OR CU=(Lebanon) OR CU=(Lesotho) OR CU=(Liberia) OR CU=(Libya) OR CU=(Macedonia, FYR) OR CU=(Madagascar) OR CU=(Malaysia) OR CU=(Malawi) OR CU=(Mali) OR TS=(Marshall Islands) OR CU=(Mauritania) OR CU=(Mauritius) OR CU=(Mexico) OR CU=(Micronesia)	17,062,517

		OR CU=(Moldova) OR CU=(Mongolia) OR CU=(Montenegro) OR CU=(Morocco) OR CU=(Mozambique) OR CU=(Myanmar) OR CU=(Namibia) OR CU=(Nepal) OR CU=(Nauru) OR CU=(Nicaragua) OR CU=(Niger) OR CU=(Nigeria) OR CU=(Pakistan) OR CU=(Papua New Guinea) OR CU=(Paraguay) OR CU=(Peru) OR CU=(Philippines) OR CU=(Romania) OR CU=(Russia) OR CU=(Rwanda) OR CU=(Samoa) OR CU=(Senegal) OR CU=(Serbia) OR CU=("Sierra Leone") OR CU=(Somalia) OR CU=(Grenadines) OR CU=(Samoan Islands) OR CU=("South Africa") OR CU=(St. Lucia) OR CU=(St. Vincent and the Grenadines) OR CU=(Sudan) OR CU=(Suriname) OR CU=(Swaziland) OR CU=(Syrian) OR CU=("Sri Lanka") OR CU=(Tajikistan) OR CU=(Tanzania) OR CU=(Thailand) OR CU=(Togo) OR CU=(Tonga) OR CU=(Tunisia) OR CU=(Turkey) OR CU=(Turkmenistan) OR CU=(Tuvalu) OR CU=(Timor-Leste) OR CU=(Uganda) OR CU=(Ukraine) OR CU=(Uzbekistan) OR CU=(Vanuatu) OR CU=(Venezuela) OR CU=(Vietnam) OR CU=(Gaza*) OR CU=(Yemen) OR CU=(Zambia) OR CU=(Zimbabwe) OR CU=(developing countr*) OR CU=(low-and middle income countr*) OR CU= (LMIC*) OR CU=(low income countr*) OR CU=(middle income countr*) OR CU=(lower middle income countr*) OR CU=(upper middle income countr*)	
43		#42 AND #41 AND #32 AND #18	628

Final search output : 628

Supplementary table 2: List of low- and middle-income countries

Serial No	County
1.	Afghanistan
2.	Albania
3.	Albania
4.	American Samoa
5.	Angola
6.	Armenia
7.	Azerbaijan
8.	Bangladesh
9.	Belarus
10.	Belize
11.	Benin
12.	Bhutan

13.	Bolivia
14.	Bosnia and Herzegovina
15.	Botswana
16.	Brazil
17.	Bulgaria
18.	Burkina Faso
19.	Burundi
20.	Cabo Verde
21.	Cambodia
22.	Cameroon
23.	Central African Republic
24.	Chad
25.	China
26.	Colombia
27.	Comoros
28.	Democratic Republic of Congo OR DR Congo OR DRC OR the Congo
29.	Congo, Rep.
30.	Costa Rica
31.	Côte d'Ivoire OR Ivory Coast
32.	Cuba
33.	Djibouti
34.	Dominica
35.	Dominican Republic
36.	Ecuador
37.	Egypt, Arab Rep.
38.	El Salvador
39.	Equatorial Guinea
40.	Eritrea
41.	Ethiopia
42.	Fiji
43.	Gabon
44.	The Gambia
45.	Georgia
46.	Ghana
47.	Grenada
48.	Guatemala
49.	Guinea
50.	Guinea-Bissau or Guinea Bissau
51.	Guyana

52.	Haiti
53.	Honduras
54.	India
55.	Indonesia
56.	Iran, Islamic Rep.
57.	Iraq
58.	Jamaica
59.	Jordan
60.	Kazakhstan
61.	Kenya
62.	Kiribati
63.	Korea, Dem. People's Rep.
64.	Kosovo
65.	Kyrgyz Republic
66.	Lao PDR
67.	Lebanon
68.	Lesotho
69.	Liberia
70.	Libya
71.	Macedonia, FYR
72.	Madagascar
73.	Malawi
74.	Malaysia
75.	Maldives
76.	Mali
77.	Marshall Islands
78.	Mauritania
79.	Mauritius
80.	Mexico
81.	Micronesia, Fed. Sts.
82.	Moldova
83.	Mongolia
84.	Montenegro
85.	Morocco
86.	Mozambique
87.	Myanmar
88.	Namibia
89.	Nauru
90.	Nepal

91.	Nicaragua
92.	Niger
93.	Nigeria
94.	Pakistan
95.	Papua New Guinea
96.	Paraguay
97.	Peru
98.	Philippines
99.	Romania
100.	Russian Federation
101.	Rwanda
102.	Samoa
103.	São Tomé and Principe OR Timor OR Timor-Liste OR Timor Liste
104.	Senegal
105.	Serbia
106.	Sierra Leone
107.	Solomon Islands
108.	Somalia
109.	South Africa
110.	South Sudan
111.	Sri Lanka
112.	Saint Lucia
113.	Vincent and the Grenadines
114.	Sudan
115.	Suriname
116.	Swaziland
117.	Syrian Arab Republic
118.	Tajikistan
119.	Tanzania
120.	Thailand
121.	Timor-Leste
122.	Togo
123.	Tonga
124.	Tunisia
125.	Turkey
126.	Turkmenistan
127.	Tuvalu
128.	Uganda

129.	Ukraine
130.	Uzbekistan
131.	Vanuatu
132.	Venezuela, RB
133.	Vietnam
134.	West Bank and Gaza
135.	Yemen, Rep.
136.	Zambia
137.	Zimbabwe

Supplementary table 3: Structured screening form for abstracts

Reviewer Code					
Abstract ID					
Name of the journal					
Title					
First Author					
Last Author					
Language	1.English<br>2. French<br>3.Chinese	4.Spanish<br>5. German<br>6. -----------------			
Study design	1. Cross sectional study<br>2. Cohort study	3. Non -randomized control trial<br>4. Randomized control trial			
Inclusion Criteria					
Population	Children under the age of five years		Yes	No	N/A
	Classified as pneumonia		Yes	No	N/A
Outcome	SpO2 status measured with pulse oximetry		Yes	No	N/A
Context	Low and middle income countries		Yes	No	N/A
Year of publication	Any study published by Dec 2018		Yes	No	N/A
Exclusion Criteria					
Sample size	Less than hundred		Yes	No	N/A
Use of pulse oximeter	To identify a particular illness		Yes	No	N/A
Screening result					
	Included for data extraction		Yes	No	Need full text review
If not included, reason for exclusion					
Additional comments (if any)					

Supplementary table 4: Extraction of data from selected articles after screening

Sl #	Item	Option		
1	Study ID			
2	Study Title			
3	First Author Last Name			
4	Last Author Last Name			
5	Year of Publication			
6	Name of the Journal			
7	Type of Study	Cross Sectional		
		Case Control		
		Cohort		
		Individual RTC		
		Cluster RCT		
		Quasi-Experimental		
8	Study Settings-Region	Asia		
		Europe		
		Africa		
		South America		
		North America		
9	Study Settings-Clinical	Referral Hospital		
		Non-Referral Health Centres		
		Community		
10	Age group	2-11 months	Yes	No
		11-59 months	Yes	No
		>59 Months	Yes	No
11	Specific Classification Reported		Yes	No
12	Type of Classification Reported	Pneumonia	Yes	No
		Severe Pneumonia	Yes	No
		ARI	Yes	No
		Others, specify	Yes	No
13	PO use Reported		Yes	No
14	Type of PO Used, specify			
15	Hypoxaemia Reported		Yes	No
16	Prevalence of Hypoxaemia in %	Overall		
		2-11 months		
		11-59 months		
		>59 Months		
		Pneumonia		
		Severe Pneumonia		

		ARI		
		Others, specify		
		Referral Hospital		
		Non-Referral Health Centres		
		Community		
		Asia		
		Europe		
		Africa		
		South America		
		North America		
17	Specific SpO2 Measurement Available		Yes	No
18	SpO2 Status in %	Overall		
		2-11 months		
		11-59 months		
		>59 Months		
		Pneumonia		
		Severe Pneumonia		
		ARI		
		Others Classification		
		Referral Hospital		
		Non-Referral Health Centres		
		Community		
		Asia		
		Europe		
		Africa		
		South America		
		North America		
19	Type of Health Care Worker measuring SpO2 reported		Yes	No
20	Type of Health Care Worker	Doctors	Yes	No
		Nurses	Yes	No
		Paramedics	Yes	No
		Community Health Worker	Yes	No
21	Oxygen Given for Hypoxaemia Reported		Yes	No
22	Type of Oxygen Given	High Flow		
		Low Flow		
23	Other Intervention Given for Hypoxaemia Reported		Yes	No
24	Other Intervention Given for Hypoxaemia Reported, specify			

25	Case Fatality Rate Reported among Hypoxaemia Cases (without intervention) Reported		Yes	No
26	Case Fatality Rate (death) among Hypoxaemia Cases (without intervention) in %	Overall		
		2-11 months		
		11-59 months		
		>59 Months		
		Pneumonia		
		Severe Pneumonia		
		ARI		
		Others, specify		
		Referral Hospital		
		Non-Referral Health Centres		
		Community		
		Asia		
		Europe		
		Africa		
		South America		
		North America		
27	Other Outcome Reported		Yes	No
28	Other Outcome Reported, please specific			

Supplementary Table 5: Overview of the studies reporting the prevalence of hypoxaemia among WHO-classified pneumonia

Author	Year	Journal	Region	Country	Altitude	Pneumonia classification	SpO2	Pulse oximetry (maker and model)
Alwadhi et al.	2017	Indian Pediatrics	Asia	India	Low	Very severe or severe pneumonia	90	Ohmeda Biox 3700e pulse oximeter
Asghar et al.	2008	BMJ Open	Multiple Continent	Bangladesh, Ecuador, India, Mexico, Pakistan, Yemen and Zambia	Low	Very severe or severe pneumonia	90	Not mentioned
Ashraf et al.	2008	Archives of Disease in Childhood, BMJ	Asia	Bangladesh	Low	Very severe or severe pneumonia	95	Not mentioned
Ashraf et al.	2010	Pediatrics	Asia	Bangladesh	Low	Very severe or severe pneumonia	95	Not mentioned
Basnet et al.	2015	PLoS ONE	Asia	Nepal	High	Very severe or severe pneumonia	90	Nellcor Puritan Bennett NPB-40, Pleasanton, CA, USA
Bassat et al.	2016	Tropical Medicine & International Health	Africa	Southern Mozambique	Low	Very severe or severe pneumonia	90	Nellcor, Boulder, CO
Breiman et al.	2015	BMC Infectious Diseases	Africa	Kenya	Low	Very severe or severe pneumonia	90	Not mentioned
Chisti et al.	2011	PLoS ONE	Asia	Bangladesh	Low	Very severe or severe pneumonia	90	Not mentioned
Dembele et al.	2019	Bmj Open	Asia	Phillipines	Low	Very severe or severe pneumonia	90	Handheld Pulse Oximeter PalmSAT 2500A with Finger
Fancourt et al.	2017	Clinical Infectious Disease	Multiple Continent	Kenya, The Gambia, Mali, Zambia, South Africa, Thailand, Bangladesh	Low	Very severe or severe pneumonia	92	Not mentioned
Gallagher et al.	2020	Clinical Infectious Diseases	Multiple Continent	Asia and Arfica	Mixed	Very severe or severe pneumonia	92	Not mentioned
Graham et al.	2020	EClinicalMedicine	Africa	Nigeria	Low	Very severe or severe pneumonia	90	Not mentioned
Izadnegahdar et al.	2012	Journal of Pediatric Infectious Diseases	Multiple Continent	Colombia, Ghana, India, Mexico, Pakistan, South Africa, Vietnam and Zambia	Low	Very severe or severe pneumonia	92	Nellcor N-20E, N-25 secsor, Pleasantom, CA, USA
Jain et al.	2013	Indian Pediatrics	Asia	India	Low	Very severe or severe pneumonia	90	Not mentioned
Kuti et al.	2013	ISRN Pediatrics	Africa	Gambia	Low	Very severe or severe pneumonia	90	Nellcor N-200, USA

Ma et al.	2019	American Journal of Tropical Medicine and Hygiene	Africa	Uganda	Low	Very severe or severe pneumonia	90	Not mention ed
McCollum et al.	2019	Lancet Respir Med	Africa	Malawi	Low	Very severe or severe pneumonia	90	Rad5 Masimo, Irvine, CA, USA
Nantanda et al.	2008	Annals of Tropical Paediatrics	Africa	Uganda	Low	Very severe or severe pneumonia	92	Not mention ed
Sigauque et al.	2009	Journal of Tropical Pediatrics	Africa	Mozambican	Low	Very severe or severe pneumonia	90	Nellcor
Semperteg ui et al.	2014	American Journal of Clinical Nutrition	South America	Ecuador	Low	Very severe or severe pneumonia	90	OxiMax N-65; Nellcor
Srinivasan et al.	2012	BMC medicine	Africa	Uganda	Low	Very severe or severe pneumonia	92	Ohmeda Biox 3700 Ohmeda GE healthca re, Pollards Wood Chalfont St. Giles Bucks HP8 4SP UK
Tesfaye et al.	2020	Bmj Open	Africa	Ethiopia	Low	Very severe or severe pneumonia	90	ADC Adimals 2150
Tiewsoh et al.	2009	BMC Pediatrics	Asia	India	Low	Very severe or severe pneumonia	90	Not mention ed
Webb et al.	2012	Journal of Pediatric Infectious Diseases	Africa	Kenya	Low	Very severe or severe pneumonia	90	Neclor USA
Blanc et al.	2019	PLoS One	Africa	PNG	Low	Unclassified pneumonia	90	Nellcor Pulse OxyMax N-20PA
Chisti et al.	2012	Tropical Medicine & International Health	Asia	Bangladesh	Low	Unclassified pneumonia	90	OxiMax N-600; Nellcor, Boulder, CO, USA
Duke et al.	2008	Lancet	Africa	Papua New Guinea	Low	Unclassified pneumonia	90	Not mention ed
Hooli et al.	2020	American Journal of Tropical Medicine and Hygiene	Africa	Malawi	Low	Unclassified pneumonia	90	Lifebox
Janabi et al.	2009	Iraqi Postgrad. Med. J.	Asia	Iraq	Low	Unclassified pneumonia	90	Kontron Medical B.P:7845
Kelly et al.	2014	Journal of the Pediatric Infectious Diseases Society	Africa	Bostwana	Low	Unclassified pneumonia	90	Not mention ed
Kurade et al.	2018	Journal of Tropical Pediatrics	Asia	India	Low	Unclassified pneumonia	90	Not mention ed
Laghari et al.	2019	Cureus	Asia	Pakistan	Low	Unclassified pneumonia	92	Not mention ed
Lima et al.	2015	Pediatric Health, Medicine, and Therapeutics	South America	Brazil	Low	Unclassified pneumonia	92	Not mention ed
Ma et al.	2019	American Journal of Tropical Medicine and Hygiene	Africa	Uganda	Low	Unclassified pneumonia	90	Not mention ed

Mackenzie et al.	2017	Lancet Infectious Disease	Africa	Gambia	Low	Unclassified pneumonia	92	Nellcor N-65, Covidian , Boulder, CO, USA
Mathisen et al.	2010	Pediatric Infectious Disease Journal	Asia	Nepal	High	Unclassified pneumonia	93	Siemens MicroO2 ,Siemens Medical Systems Inc,Danvers, MA with Nellcor, Pleasnton,CA
McCollum et al.	2017	Plos One	Africa	Malawi	Low	Unclassified pneumonia	90	Masimo (R) Hospitals or Lifebox (R)
McCollum et al.	2016	Bulletin of the World Health Organization	Africa	Malawi	Low	Unclassified pneumonia	90	Lifebox
McCracken et al.	2013	The Journal of Infectious Diseases	South America	Guatemala	High	Unclassified pneumonia	90	Not mentioned
Orimadegun et al.	2013	Transactions of the Royal Society of Tropical Medicine and Hygiene	Africa	Nigeria	Low	Unclassified pneumonia	90	True Colour OLED fingertip oximeters
Rao et al.	2012	World Journal of Pediatrics	Asia	India	Low	Unclassified pneumonia	90	Nellcor Puritan Bannet NPB-40
Shrestha et al.	2020	Plos One	Asia	Nepal	High	Unclassified pneumonia	90	Mini SPO2, Criticare Systems, USA
Smith et al.	2011	Lancet	South America	Guatemala	High	Unclassified pneumonia	87	Not mentioned
Thomas et al.	2015	Tropical Medicine & International Health	Asia	Philippines	Low	Unclassified pneumonia	90	Not mentioned
Tran et al.	2016	Epidemiol Infect.	Asia	Vietnam	Low	Unclassified pneumonia	92	Siemens Micro Oe, Siemens Medical Systems Inc, USA
Tran et al.	2014	Journal of Medical Virology	Asia	Vietnam	Low	Unclassified pneumonia	92	Not mentioned
Yalçın et al.	2018	Indian Journal of Pediatrics	Asia	Turkey	Low	Unclassified pneumonia	93	PM-60, Mindray, China
Oktaria et al.	2021	PLoS One	Asia	Indonesia	Low	Unclassified pneumonia	90	Not mentioned
Roux et al.	2021	PLoS One	Africa	South Africa	low	Unclassified pneumonia	92	Not mentioned
Saleh et al.	2021	Padiatric Research	Africa	Egypt	Low	Unclassified pneumonia	94	Not mentioned

Rahman et al.	2021	Journal of Global Health	Asia	Bangladesh	low	Severe pneumonia	90	OxiMax N-600; Nellcor, Boulder, CO, USA
Muro et al.	2020	PLOS ONE	Africa	Tanzania	Low	Severe pneumonia	90	Not mention ed
Lenahan et al.	2020	Erj Open Research	Africa	Malawi	Low	Non-severe pneumonia	90	Not mention ed
Morre et al.	2019	Journal of Tropical Pediatrics	Africa	Papua New Guinea	Low	Non-severe pneumonia	90	Not mention ed
Mwaniki et al.	2009	Bulletin of the World Health Organization	Africa	Kenya	Low	Non-severe pneumonia	90	Nellcor Puritan Bennett NPB-40, USA
Nemani et al.	2016	Clinical Epidemiology and Global health	Asia	India	Low	Non-severe pneumonia	92	SHO 3002, Harsons, India
Puumalaine n et al.	2008	BMC Infectious Diseases	Asia	Philippines	Low	Non-severe pneumonia	90	BCI 3303 ximeter (Waukeh a, USA)

Supplementary Table 6.1: Quality Assessment of the Studies with WHO pneumonia classification

Serial	Author	Was the sample frame appropriate to address the target population?	Were study participants sampled in an appropriate way?	Was the sample size adequate?	Were the study subjects and the setting described in detail?	Was the data analysis conducted with sufficient coverage of the identified sample?	Were valid methods used for the identification of the condition?	Was the condition measured in a standard, reliable way for all participants?	Was there appropriate statistical analysis?	Was the response rate adequate, and if not, was the low response rate managed appropriately?
1	Alwadhi et al.	Yes	Yes	Yes	Yes	Yes	Yes	Yes	Yes	Yes
2	Asghar et al.	Yes	Yes	Yes	Yes	Yes	Yes	Yes	Yes	Yes
3	Ashraf et al.	Yes	Yes	Yes	Yes	Yes	Yes	Yes	Yes	Yes
4	Ashraf et al.	Yes	Yes	Yes	Yes	Yes	Yes	Yes	Yes	Yes
5	Basnet et al.	Yes	Yes	Yes	Yes	Yes	Yes	Yes	Yes	Yes
6	Bassat et al.	Yes	Yes	Yes	Yes	Yes	Yes	Yes	Yes	Yes
7	Breiman et al.	Yes	Yes	Yes	Yes	Yes	Yes	Yes	Yes	Yes
8	Chisti et al.	Yes	Yes	Yes	Yes	Yes	Yes	Yes	Yes	Yes
9	Dembele et al.	Yes	Yes	Yes	Yes	Yes	Yes	Yes	Yes	Yes
10	Fancourt et al.	Yes	Yes	Yes	Yes	Yes	Yes	Yes	Yes	Yes
11	Gallagher et al.	Yes	Yes	Yes	Yes	Yes	Yes	Yes	Yes	Yes
12	Graham et al.	Yes	Yes	Yes	Yes	Yes	Yes	Yes	Yes	Yes
13	Izadnegahdar et al.	Yes	Yes	Yes	Yes	Yes	Yes	Yes	Yes	Yes
14	Jain et al.	Yes	Yes	Yes	Yes	Yes	Yes	Yes	Yes	Yes
15	Kuti et al.	Yes	Yes	Yes	Yes	Yes	Yes	Yes	Yes	Yes
16	Ma et al.	Yes	Yes	Yes	Yes	Yes	Yes	Yes	Yes	Yes
17	McCollum et al.	Yes	Yes	Yes	Yes	Yes	Yes	Yes	Yes	Yes
18	Nantanda et al.	Yes	Yes	Yes	Yes	Yes	Yes	Yes	Yes	Yes
19	Sigauque et al.	Yes	Yes	Yes	Yes	Yes	Yes	Yes	Yes	Yes
20	Sempertegui et al.	Yes	Yes	Yes	Yes	Yes	Yes	Yes	Yes	Yes
21	Srinivasan et al.	Yes	Yes	Yes	Yes	Yes	Yes	Yes	Yes	Yes
22	Tesfaye et al.	Yes	Yes	Yes	Yes	Yes	Yes	Yes	Yes	Yes

23	Tiewsoh et al.	Yes	Yes	Yes	Yes	Yes	Yes	Yes	Yes	Yes
24	Webb et al.	Yes	Yes	Yes	Yes	Yes	Yes	Yes	Yes	Yes
25	Blanc et al.	Yes	Yes	Yes	Yes	Yes	Yes	Yes	Yes	Yes
26	Chisti et al.	Yes	Yes	Yes	Yes	Yes	Yes	Yes	Yes	Yes
27	Duke et al.	Yes	Yes	Yes	Yes	Yes	Yes	Yes	Yes	Yes
28	Hooli et al.	Yes	Yes	Yes	Yes	Yes	Yes	Yes	Yes	Yes
29	Janabi et al.	Yes	Yes	Yes	Yes	Yes	Yes	Yes	Yes	Yes
30	Kelly et al.	Yes	Yes	Yes	Yes	Yes	Yes	Yes	Yes	Yes
31	Kurade et al.	Yes	Unclear	Yes	Yes	Yes	Yes	Yes	Yes	Yes
32	Laghari et al.	Yes	Yes	Yes	Yes	Yes	Yes	Yes	Yes	Yes
33	Lima et al.	Yes	Yes	Yes	Yes	Yes	Yes	Yes	Yes	Yes
34	Ma et al.	Yes	Yes	Yes	Yes	Yes	Yes	Yes	Yes	Yes
35	Mackenzie et al.	Yes	Yes	Yes	Yes	Yes	Yes	Yes	Yes	Yes
36	Mathisen et al.	Yes	Yes	Yes	Yes	Yes	Yes	Yes	Yes	Yes
37	McCollum et al.	Yes	Yes	Yes	Yes	Yes	Yes	Yes	Yes	Yes
38	McCollum et al.	Yes	Yes	Yes	Yes	Yes	Yes	Yes	Yes	Yes
39	McCracken et al.	Yes	Yes	Yes	Yes	Yes	Yes	Yes	Yes	Yes
40	Orimadegun et al.	Yes	Yes	Yes	Yes	Yes	Yes	Yes	Yes	Yes
41	Rao et al.	Unclear	Yes	No	Yes	Yes	Yes	Yes	Yes	Yes
42	Saleh et al.	Yes	Yes	Yes	Yes	Yes	Yes	Yes	Yes	Yes
43	Smith et al.	Yes	Yes	Yes	Yes	Yes	Yes	Yes	Yes	Yes
44	Thomas et al.	Yes	Yes	Yes	Yes	Yes	Yes	Yes	Yes	Yes
45	Tran et al.	Yes	Yes	Yes	Yes	Yes	Yes	Yes	Yes	Yes
46	Tran et al.	Yes	Yes	Yes	Yes	Yes	Yes	Yes	Yes	Yes
47	Yalçın et al.	Yes	Yes	Yes	Yes	Yes	Yes	Yes	Yes	Yes
48	Lenahan et al.	Yes	Yes	Yes	Yes	Yes	Yes	Yes	Yes	Yes
49	Morre et al.	Yes	Yes	Yes	Yes	Yes	Yes	Yes	Yes	Yes
50	Mwaniki et al.	Yes	Yes	Yes	Yes	Yes	Yes	Yes	Yes	Yes
51	Nemani et al.	Yes	Yes	Yes	Yes	Yes	Yes	Yes	Yes	Yes
52	Puumalainen et al.	Yes	Yes	Yes	Yes	Yes	Yes	Yes	Yes	Yes
53	Oktaria et al.	Yes	Yes	Yes	Yes	Yes	Yes	Yes	Yes	Yes
54	Rahman et al.	Yes	Yes	Yes	Yes	Yes	Yes	Yes	Yes	Yes
55	Roux et al.	Yes	Yes	Yes	Yes	Yes	Yes	Yes	Yes	Yes
56	Shrestha et al.	Yes	Yes	Yes	Yes	Yes	Yes	Yes	Yes	Yes
57	Muro et al.	Yes	Yes	Yes	Yes	Yes	Yes	Yes	Yes	Yes

Supplementary Table 6.2: Quality Assessment of the Studies with other lab diagnosed pneumonia classification

Serial	Author	Was the sample frame appropriate to address the target population?	Were study participants sampled in an appropriate way?	Was the sample size adequate?	Were the study subjects and the setting described in detail?	Was the data analysis conducted with sufficient coverage of the identified sample?	Were valid methods used for the identification of the condition?	Was the condition measured in a standard, reliable way for all participants?	Was there appropriate statistical analysis?	Was the response rate adequate, and if not, was the low response rate managed appropriately?
1	Abdulkadir et al.	Yes	Yes	Yes	Yes	Yes	Yes	Yes	Yes	Yes
2	Araya et al.	Yes	Yes	Yes	Yes	Yes	Yes	Yes	Yes	Yes
3	Benet et al.	Yes	Yes	Yes	Yes	Yes	Yes	Yes	Yes	Yes
4	Chisti et al.	No	Yes	No	Yes	Yes	Yes	Yes	Yes	Yes
5	Ciftci et al.	Yes	Yes	Yes	Yes	Yes	Yes	Yes	Yes	Yes
6	Fashanu et al.	Yes	Yes	Yes	Yes	Yes	Yes	Yes	Yes	Yes
7	Kabir et al.	Yes	Yes	Yes	Yes	Yes	Yes	Yes	Yes	Yes
8	Nathan et al.	Yes	Yes	Yes	Yes	Yes	Yes	Yes	Yes	Yes
9	Nyawanda et al.	Yes	Yes	Yes	Yes	Yes	Yes	Yes	Yes	Yes
10	Pascoal et al.	Yes	Yes	Yes	Yes	Yes	Yes	Yes	Yes	Yes
11	Pervaiz et al.	Yes	Yes	Yes	Yes	Yes	Yes	Yes	Yes	Yes
12	Pukai et al.	Yes	Yes	Yes	Yes	Yes	Yes	Yes	Yes	Yes
13	Rani et al.	Yes	Yes	Yes	Yes	Yes	Yes	Yes	Yes	Yes
14	Reed et al.	Yes	Yes	Yes	Yes	Yes	Yes	Yes	Yes	Yes
15	Ueno et al.	Yes	Yes	Yes	Yes	Yes	Yes	Yes	Yes	Yes
16	Falade et al.	Yes	Yes	Yes	Yes	Yes	Yes	Yes	Yes	Yes
17	Alfonso et al.	Yes	Yes	Yes	Yes	Yes	Yes	Yes	Yes	Yes
18	Stoiber et al.	Yes	Yes	Yes	Yes	Yes	Yes	Yes	Yes	Yes
19	Mahmoud et al.	Yes	Yes	Yes	Yes	Yes	Yes	Yes	Yes	Yes

Supplementary Table 7: I^2 and τ^2 value for measure of heterogeneity across studies

Categories	Sub-categories	Heterogeneity statistic	DF	P-value	I^2	τ^2
Region	Asia	2722.26	23	0	99.16%	0.16
	Africa	6815.26	27	0	99.60%	0.13
	South America	1163.17	4	0	99.66%	0.32
	Multiple continent	649.64	3	0	99.54%	0.12
Altitude	High	736.45	5	0	99.32%	0.13
	Low	13687.19	53	0	99.61%	0.17
Facility type	Tertiary level referral facilities	3886.90	31	0	99.20%	0.21
	Primary or secondary referral facilities	1109.11	12	0	98.92%	0.11
	Health centres	604.13	5	0	99.17%	0.17
	Mixed setting	1794.99	5	0	99.72%	0.2
Type of patients	Emergency	476.44	7	0	98.53%	0.25
	Inpatient	4935.40	34	0	99.31%	0.15
	Outpatient	2270	12	0	99.47%	0.16
	Mixed	931.8	4	0	99.57%	0.04
Hospitalisation status	Hospitalised	6166.21	42	0	99.32%	0.18
	Non-hospitalised	2515.64	12	0	99.52%	0.19
	Mixed	918.55	3	0	99.67%	0.04
Clinical severity	Non-severe pneumonia	108.07	4	0	96.30%	0.06
	Unclassified pneumonia	5496.68	27	0	99.51%	0.1
	Very severe or severe pneumonia	5013.99	27	0	99.46%	0.18
Assessor	Doctor	2674.06	15	0	99.44%	0.32
	Nurse/paramedics	3691.52	12	0	99.67%	0.08
WHO IMCI 2014 classification	Pre	13470.43	47	0	99.65%	0.16
	Post	1261.32	12	0	99.05%	0.25
Publication Year	Before 2015	4087.45	27	0	99.34%	0.20
	2015 and after	9981.15	32	0	99.68%	0.15
SpO2 Cut off	90%	10525.38	41	0	99.61%	0.18
	Others	5108.22	18	0	99.65%	0.24

Supplementary table 8: Number of articles using different database

Database	#
MEDLINE	425
EMBASE	807
GLOBAL HEALTH	130
Cochrane	153
Clinicaltrails.gov	85

SCOPUS	665
CINHAL	66
PubMed	907
Global Index Medicus (WHO)	376
IndMed (Advanced)	33
Web of Science (Advanced)	628
	4275

Supplementary table 9: Number of studies across sub-groups among WHO classified pneumonia

Categories	Sub-categories	Number of Studies (#57)
Region	Asia	23
	Africa	26
	South America	4
	Multiple continent	4
Altitude	High	5
	Low	51
	Mixed	1
Facility type	Tertiary level referral facilities	30
	Primary or secondary referral facilities	12
	Health centres	5
	Mixed setting	6
	Not reported	3
Type of patients	Emergency	8
	Inpatient	32
	Outpatient	12
	Mixed	5
Hospitalisation status	Hospitalised	40
	Non-hospitalised	12
	Mixed	4
	Not reported	1
Assessor	Doctor	15
	Nurse/paramedics	12
	Doctors/nurses/paramedics	2
	Non clinical	2
	Not mentioned	23
PO type mentioned	Yes	29
	No	28
PO probe type	Adult	1
	Paediatric	13
	Not reported	43
SpO2 cut off	87%	1
	90%	38

	92%	12
	93%	2
	94%	1
	95%	2
	90-93%	1

Supplementary table 10: Prevalence of hypoxaemia among WHO -classified pneumonia before and after the change in pneumonia classification in 2014

Pneumonia classification	IMCI	Prevalence of hypoxaemia	Lower limit	Upper limit
Very severe or severe	Pre 2014 (n = 24)	41	32	50
	2014 and after (n = 4)	40	37	43
Non-severe	Pre 2014 (n = 3)	10	2	24
	2014 and after (n = 2)	7	5	10
Unclassified	Pre 2014 (n = 21)	23	18	29
	2014 and after (n = 7)	32	9	60
Overall	Pre 2014 (n = 48)	31	26	36
	2014 and after (n = 13)	29	17	42

Supplementary table 11: Hypoxaemia prevalence (presented in %) among children with WHO - classified pneumonia, comparison between random-effects pooled estimate (95% confidence interval) and median (with interquartile range) across different subgroups

			95% Confidence Interval				
		Random Effects pooled estimate	Lower limit	Upper limit	Median	Q1	Q3
	**Overall Pooled WHO Pneumonia**	**31**	**26**	**36**	29	14	49
**Region**	Asia	31	24	39	32	16	54
	Africa	28	22	34	27	9	40
	South America	38	16	62	28	26	51
	Multiple continent	36	21	54	34	24	50

**Altitude**	High	33	20	47	29	26	58
	Low	30	25	35	28	14	49
**Facility Type**	Referral - Tertiary	29	22	36	28	15	44
	Referral - Primary / Secondary	42	33	51	40	28	57
	Health Centre	25	12	40	26	9	44
	Mixed setting	39	23	57	36	13	65
**Type of patients**	Emergency	47	30	64	50	33	60
	Inpatient	32	26	38	32	18	42
	Outpatient	23	15	33	26	7	35
	Mixed	17	11	24	16	8	18
**Hospitalisation Status**	Hospitalised	33	27	39	32	18	49
	Non-hospitalised	29	19	40	30	9	51
	Mixed	17	10	24	12	6	34
**Clinical severity**	Very severe or severe	41	33	49	39	27	57
	Unclassified	25	20	31	26	9	38
	Non-severe	8	3	16	8	4	14
**Assessor**	Doctor	41	28	55	39	23	58
	Nurse / paramedics	16	10	23	9	5	28
**WHO IMCI 2014 classification**	Pre	31	26	36	28	15	51
	Post	29	17	42	32	14	42
**Publication Year**	Before 2015	33	25	41	30	18	54
	2015 and after	29	23	35	28	13	42
**SpO2 Cut off**	≤90%	32	26	38	30	14	55
	Others	27	18	38	29	18	36

Supplementary table 12: List of articles dropped with reason

ID	Title	Reason for exclusion
1	Nasal continuous positive airway pressure combined midazolam for micro pump infusion for severe pneumonia in infants	Full text not found
2	Accuracy of selected devices for the detection of pneumonia symptoms-results from laboratory testing and field evaluations of respiratory rate counters and pulse oximeters with frontline health workers in sub-Saharan Africa and Southeast Asia	Full text not found
3	Non-invasive positive pressure ventilation to reduce childhood mortality from acute respiratory failure in rural Ghana	Full text not found
4	Clinical predictors of hypxemia in children with acute respiratory tract infection admitted in pediatric department of Southe Valley university hospital	Full text not found
5	Cost illness of RSV infection in Colombia	Full text not found
6	Discrimination of pneumonia from other lower respiratory tract infections (LRTI) in children aged 0 to 5 years living at high altitude in Peru	Full text not found
7	Viral and bacterial causes of severe acute respiratory illness among children aged less than 5 years in a high malaria prevalence area of Western Kenya, 2007-2010	Full text not found
8	Pneumocystis jirovecii pneumonia among HIV-exposed, uninfected children in Botswana	Full text not found
9	Can we distinguish pneumonia from wheezy diseases in tachypnoeic children under low-resource conditions? A prospective observational study in four Indian hospitals	Full text not found
10	Impact of Human Immunodeficiency Virus Infection on the Etiology and Outcome of Severe Pneumonia in Malawian Children	Full text not found
11	The combination of CRP,myocardial enzymes measurement and blood gas analysis in neonatal pneumonia	Full text not found
12	Detection of myocardial enzymes, cardiac troponin T and hepatic and renal function in the diagnosis and treatment of severe pneumonia in children	Full text not found
13	Value of lung ultrasound on diagnosing transient tachypnea of newborn	Full text not found
14	Clinical characteristics of Mycoplasma pneumoniae pneumonia with different imaging changes in children	Full text not found
15	Effect of Tanreqing injection assisted Ganciclovir on children with respiratory syncytial virus pneumonia	Full text not found
16	[Nasal continuous positive airway pressure ventilation in children with community-acquired pneumonia under five years of age: a prospective, multi-center clinical study]	Full text not found
17	Role of zinc in severe pneumonia	Full text not found
18	Performance of the world health organization diagnostic criteria for pneumonia in hospitalized Malawian infants with exposure to human immunodeficiency virus	Full text not found
19	Assessment of pneumonia in children in resource-limited settings	Full text not found
20	Stunting predicts poor outcome in children with severe pneumonia	Full text not found
21	Blood oxygen saturation levels associated with respiratory viruses in children hospitalized with pneumonia in Guatemala, 2008-2011	Full text not found
22	Clinical and radiologic features of pneumonia caused by staphylococcus aureus and streptococcus pneumoniae	Full text not found
23	Obstructive respiratory disease complicating pneumonia in children; possible role of viruses in a resource poor setting	Full text not found
24	An observational study of peripheral muscle oxygenation using nirs in a cohort of nourished and malnourished children with pneumonia	Full text not found
25	Predictors of treatment failure among infants with acute lower respiratory infection due to respiratory syncytial virus in Botswana	Full text not found
26	Lactate as a predictor of mortality in Malawian children with WHO-defined pneumonia	Full text not found
27	Predictors of prolonged length of hospital stay for infants with bronchiolitis	Full text not found
28	Efficacy of Bronchodilators in Wheeze Associated Respiratory Tract Infections	Full text not found
29	Prevalence of hypoxemia in under-five children with pneumonia in an emergency pediatrics hospital in Sudan	Full text not found
30	[Analysis of clinical manifestations of 159 hospitalized children infected with 2009 novel influenza A (H1N1) virus]. [Chinese]	Full text not found
31	Prevalence of human coronavirus infection in children with acute lower respiratory tract infection	Full text not found
32	Barriers to pediatric inpatient care guideline adherence: A mixed method assessment of eight hospitals in Asia and Africa	Full text not found
33	Human rhinovirus infections in hospitalized children: clinical, epidemiological and virological features	Full text not found

34	Viral and bacterial etiologies of community-acquired acute lower respiratory infections among hospitalized Cambodian patients	Full text not found
35	Efficacy of mechanical expectoration combined with oxygen inhalation treatment of infant bronchiolitis	Full text not found
36	Interventional bronchoscopy via laryngeal mask airway (LMA) under general anesthesia in children using adult flexible bronchoscope	Full text not found
37	Mortality and morbidity of acute hypoxemic respiratory failure and acute respiratory distress syndrome in infants and young children	Full text not found
38	Response to oxygen therapy using oxygen concentrators run off solar power in children with respiratory distress in remote primary health facilities in Papua New Guinea	Full text not found
39	Clinical Profile and Predictors of Outcome of Pediatric Acute Respiratory Distress Syndrome in a PICU: A Prospective Observational Study	Full text not found
40	Determinants of Outcome among Under-Five Children Hospitalized with Pneumonia at a Tertiary Health Facility in South-West Nigeria	Full text not found
41	Efficacy of high flow nasal oxygen therapy in children with acute respiratory failure	Full text not found
42	Clinical profile, severity pattern and socio-demographic risk factors of acute lower respiratory tract infection (Alrti) in children in Enugu, Nigeria	Full text not found
43	Hypoxemia predicts death from severe falciparum malaria among children under 5 years of age in Nigeria: The need for pulse oximetry in case management	Hypoxaemia prevalence/denominator could not be found
44	Density of upper respiratory colonization with Streptococcus pneumoniae and its role in the diagnosis of pneumococcal pneumonia among children aged <5 years in the PERCH study	Hypoxaemia prevalence/denominator could not be found
45	Nutritional rickets and vitamin D deficiency - Association with the outcomes of childhood very severe pneumonia: A prospective cohort study	Hypoxaemia prevalence/denominator could not be found
46	Zinc supplementation in severe acute lower respiratory tract infection in children: A triple-blind randomized placebo controlled trial	Hypoxaemia prevalence/denominator could not be found
47	High Mortality Risk in Hypoglycemic and Dysglycemic Children Admitted at a Referral Hospital in a Non Malaria Tropical Setting of a Low Income Country	Hypoxaemia prevalence/denominator could not be found
48	Oil instillation pneumonia - A social evil	Hypoxaemia prevalence/denominator could not be found
49	Child mortality after discharge from a health facility following suspected pneumonia, meningitis or septicaemia in rural Gambia: A cohort study	Hypoxaemia prevalence/denominator could not be found
50	Predictors of death in under-five children with diarrhoea admitted to a critical care ward in an urban hospital in Bangladesh	Hypoxaemia prevalence/denominator could not be found
51	Bubble continuous positive airway pressure for children with severe pneumonia and hypoxaemia in Bangladesh: an open, randomised controlled trial	Hypoxaemia prevalence/denominator could not be found
52	Incidence and characteristics of early childhood wheezing, Dhaka, Bangladesh, 2004-2010	Hypoxaemia prevalence/denominator could not be found
53	Safety of induced sputum collection in children hospitalized with severe or very severe pneumonia	Hypoxaemia prevalence/denominator could not be found
54	Humoral immune responses to Pneumocystis jirovecii antigens in HIV-infected and uninfected young children with Pneumocystis pneumonia	Hypoxaemia prevalence/denominator could not be found
55	Utility and feasibility of integrating pulse oximetry into the routine assessment of young infants at primary care clinics in Karachi, Pakistan: A cross-sectional study	Hypoxaemia prevalence/denominator could not be found
56	Early predictors of unresponsiveness to high-flow nasal cannula therapy in a pediatric emergency department	Hypoxaemia prevalence/denominator could not be found
57	Altered regulatory cytokine profiles in cases of pediatric respiratory syncytial virus infection	Hypoxaemia prevalence/denominator could not be found
58	Clinical predictors of chest radiographic abnormalities in young children hospitalized with bronchiolitis: a single center study	Hypoxaemia prevalence/denominator could not be found
59	Improved oxygen systems in district hospitals in Lao PDR: a prospective field trial of the impact on outcomes for childhood pneumonia and equipment sustainability	Hypoxaemia prevalence/denominator could not be found
60	Oral salbutamol for symptomatic relief in mild bronchiolitis a double blind randomized placebo controlled trial	Hypoxaemia prevalence/denominator could not be found
61	Hypoxaemia in hospitalised under-five Nigerian children with pneumonia	Hypoxaemia prevalence/denominator could not be found
62	Etiology of severe pneumonia in Ecuadorian children	Hypoxaemia prevalence/denominator could not be found
63	Pentraxin 3 as a clinical marker in children with lower respiratory tract infection	Hypoxaemia prevalence/denominator could not be found
64	Non-treatment of children with community health worker-diagnosed fast-breathing pneumonia in rural Malawi: Exploratory subanalysis of a prospective cohort study	Hypoxaemia prevalence/denominator could not be found
65	Can we predict oral antibiotic treatment failure in children with fast-breathing pneumonia managed at the community level? A prospective cohort study in Malawi	Hypoxaemia prevalence/denominator could not be found

66	Comparing the efficacy of 7%, 3% and 0.9% saline in moderate to severe bronchiolitis in infants	Hypoxaemia prevalence/denominator could not be found
67	Clinical predictors of hospital admission in acute lower respiratory tract infection in 2 months to 2-year-old children	Hypoxaemia prevalence/denominator could not be found
68	Pneumocystis pneumonia in South African children diagnosed by molecular methods	Hypoxaemia prevalence/denominator could not be found
69	Clinical outcomes of children with acute asthma and pneumonia in Mulago hospital, Uganda: a prospective study	Hypoxaemia prevalence/denominator could not be found
70	Respiratory tract cryptosporidiosis is common in HIV-seronegative children with intestinal cryptosporidium and cough	Hypoxaemia prevalence/denominator could not be found
71	Impaired gas exchange: accuracy of defining characteristics in children with acute respiratory infection. (Special Issue: Doctorate education and producing knowledge in nursing.)	Hypoxaemia prevalence/denominator could not be found
72	Phenotypical characterization of human rhinovirus infections in severely premature children	Hypoxaemia prevalence/denominator could not be found
73	Development and Internal Validation of a Predictive Model Including Pulse Oximetry for Hospitalization of Under-Five Children in Bangladesh	Hypoxaemia prevalence/denominator could not be found
74	Normal saline vs. hypertonic saline nebulization for acute bronchiolitis: a randomized clinical trial	Hypoxaemia prevalence/denominator could not be found
75	Sustainability in the AAP Bronchiolitis Quality Improvement Project	Hypoxaemia prevalence/denominator could not be found
76	Efficacy and safety of Laggera pterodonta in children 3-24 months with acute bronchiolitis: a randomized controlled trial	Hypoxaemia prevalence/denominator could not be found
77	Relation between pulse oximetry and clinical score in infants with acute bronchiolitis	Hypoxaemia prevalence/denominator could not be found
78	Safety and efficacy of phenylephrine nasal drops in bronchiolitis	Hypoxaemia prevalence/denominator could not be found
79	Clinical and epidemiological characteristics of pandemic influenza A/(H1N1) in hospitalized pediatric patients at a University Hospital, Istanbul, Turkey	Hypoxaemia prevalence/denominator could not be found
80	EV-D68 infection in children with asthma exacerbation and pneumonia in Mexico City during 2014 autumn	Hypoxaemia prevalence/denominator could not be found
81	Y Influenza in patients with hematological malignancies: Experience at two comprehensive cancer centers	Hypoxaemia prevalence/denominator could not be found
82	Providing oxygen to children and newborns: a multi-faceted technical and clinical assessment of oxygen access and oxygen use in secondary-level hospitals in southwest Nigeria	Hypoxaemia prevalence/denominator could not be found
83	Antenatal corticosteroids for women at risk of preterm delivery: the "Emperor's New Clothes" tale in medical practice	Hypoxaemia prevalence/denominator could not be found
84	Acute respiratory illness among a prospective cohort of pediatric patients using emergency medical services in India: Demographic and prehospital clinical predictors of mortality	Hypoxaemia prevalence/denominator could not be found
85	High-resolution Chest CT Features and Clinical Characteristics of Patients Infected with COVID-19 in Jiangsu, China	Hypoxaemia prevalence/denominator could not be found
86	Oxygen systems to improve clinical care and outcomes for children and neonates: A stepped-wedge cluster-randomised trial in Nigeria	Hypoxaemia prevalence/denominator could not be found
87	Paediatric acute respiratory distress syndrome incidence and epidemiology (PARDIE): an international, observational study	Hypoxaemia prevalence/denominator could not be found
88	Identify clinical factors related to Mycoplasma pneumoniae pneumonia with hypoxia in children	Hypoxaemia prevalence/denominator could not be found
89	Respiratory Syncytial Virus Vaccination during Pregnancy and Effects in Infants	Hypoxaemia prevalence/denominator could not be found
90	Respiratory compromise in children presenting to an urban emergency department of a tertiary hospital in Tanzania: a descriptive cohort study	Hypoxaemia prevalence/denominator could not be found
91	Treatment of respiratory distress syndrome in premature infants: A comparison of LISA and INSURE methods in the Vietnamese context	Hypoxaemia prevalence/denominator could not be found
92	Nebulised normal saline in moderate acute bronchiolitis and pneumonia in a low- to middle-income country: a randomised trial in Papua New Guinea	Hypoxaemia prevalence/denominator could not be found
93	Bubble CPAP and oxygen for child pneumonia care in Malawi: a CPAP IMPACT time motion study	Hypoxaemia prevalence/denominator could not be found
94	Usability and acceptability of a multimodal respiratory rate and pulse oximeter device in case management of children with symptoms of pneumonia: A cross-sectional study in Ethiopia	Hypoxaemia prevalence/denominator could not be found
95	Seizure in Children Under Five Presenting with Pneumonia in a Critical Care Ward in Bangladesh: Prevalence, Associated Factors, and Outcome	Hypoxaemia prevalence/denominator could not be found
96	Response to oxygen therapy using oxygen concentrators run off solar power in children with respiratory distress in remote primary health facilities in Papua New Guinea	Hypoxaemia prevalence/denominator could not be found

97	Randomised controlled trial of oxygen therapy and high-low nasal therapy in African children with pneumonia	Hypoxaemia prevalence/denominator could not be found
98	Performance of five pulse oximeters to detect hypoxaemia as an indicator of severe illness in children under five by frontline health workers in low resource settings A prospective, multicentre, single-blinded, trial in Cambodia, Ethiopia, South Sudan, and Uganda	Hypoxaemia prevalence/denominator could not be found
99	Oxygen systems and quality of care for children with pneumonia, malaria and diarrhoea: Analysis of a stepped-wedge trial in Nigeria	Hypoxaemia prevalence/denominator could not be found
100	Limitations to current methods to estimate cause of death: a validation study of a verbal autopsy model	Hypoxaemia prevalence/denominator could not be found
101	Human bocavirus-1 screening in infants with acute lower respiratory tract infection	Hypoxaemia prevalence/denominator could not be found
102	Etiology, clinical characteristics and coinfection status of bronchiolitis in Suzhou	Hypoxaemia prevalence/denominator could not be found
103	Near-Infrared Spectroscopy Monitoring of Cerebral Oxygenation and Influencing Factors in Neonates from High-Altitude Areas	Hypoxaemia prevalence/denominator could not be found
104	Estimated Cost-effectiveness of Solar-Powered Oxygen Delivery for Pneumonia in Young Children in Low-Resource Settings	Hypoxaemia prevalence/denominator could not be found
105	Employing learning health system principles to advance research on severe neonatal and paediatric illness in Kenya	Hypoxaemia prevalence/denominator could not be found
106	Effect of ten-valent pneumococcal conjugate vaccine introduction on pneumonia hospital admissions in Fiji: a time-series analysis	Hypoxaemia prevalence/denominator could not be found
107	Clinical characteristics of 967 children with pertussis: a single-center analysis over an 8-year period in Beijing, China	Hypoxaemia prevalence/denominator could not be found
108	Acute respiratory illness among a prospective cohort of pediatric patients using emergency medical services in India: Demographic and prehospital clinical predictors of mortality	Hypoxaemia prevalence/denominator could not be found
109	The impact of pulse oximetry and Integrated Management of Childhood Illness (IMCI) training on antibiotic prescribing practices in rural Malawi: A mixed-methods study	Hypoxaemia prevalence/denominator could not be found
110	Aerosol inhalation of ambroxol hydrochloride combined with terbutaline can promote recovery of children with severe pneumonia	Hypoxaemia prevalence/denominator could not be found
111	Clinico-virological profile, intensive care needs, and outcome of infants with acute viral bronchiolitis: A prospective observational study	Hypoxaemia prevalence/denominator could not be found
112	Risk factors for mortality from severe community-acquired pneumonia in hospitalized children transferred to the pediatric intensive care unit	Hypoxaemia prevalence/denominator could not be found
113	Comparison of 3% saline and 0.9% normal saline nebulization as diluent in children with bronchiolitis	Hypoxaemia prevalence/denominator could not be found
114	Morbidity pattern and outcome of children admitted to a paediatric intensive care unit of Eastern India	Hypoxaemia prevalence/denominator could not be found
115	Effect of cefoperazone sulbactam sodium combined with meropenem on the immune function in the treatment of neonatal pneumonia caused by multidrug-resistant bacteria	Hypoxaemia prevalence/denominator could not be found
116	A multicenter survey of pediatric flexible bronchoscopy in western China	Hypoxaemia prevalence/denominator could not be found
117	Epidemiology and genetic characterization of respiratory syncytial virus in children with acute respiratory infections: Findings from the influenza sentinel surveillance network in Central African Republic, 2015 to 2018	Hypoxaemia prevalence/denominator could not be found
118	Survival and predictors of mortality among severe acute malnourished under-five children admitted at Felege-Hiwot comprehensive specialized hospital, northwest, Ethiopia: a retrospective cohort study	Hypoxaemia prevalence/denominator could not be found
119	Severe recurrent pneumonia in children: Underlying causes and clinical profile in Vietnam	Hypoxaemia prevalence/denominator could not be found
120	Antibiotic-Resistant Bacteremia in Young Children Hospitalized with Pneumonia in Bangladesh Is Associated with a High Mortality Rate	Hypoxaemia prevalence/denominator could not be found
121	Saturation oxygenation pressure index: a non-invasive bedside measure for severity of respiratory disease in neonates on CPAP	Hypoxaemia prevalence/denominator could not be found
122	Optimal level of positive end-expiratory pressure during nasal continuous airway pressure for severe bronchiolitis: a prospective study	Hypoxaemia prevalence/denominator could not be found
123	Complications of pneumonia and its associated factors in a pediatric population in Osogbo, Nigeria	Hypoxaemia prevalence/denominator could not be found
124	A multicentered study on efficiency of noninvasive ventilation procedures (SAFE-NIV)	Hypoxaemia prevalence/denominator could not be found
125	Risk Factors for Pneumonia Mortality in Under-five Children in a Tertiary Care Hospital of Darjeeling District of West Bengal: A Prospective Case-Control Study	Hypoxaemia prevalence/denominator could not be found
126	Hypoglycaemia in children aged 1 month to 17 years admitted to the children's emergency room of Nnamdi Azikiwe University Teaching Hospital, Nnewi, Nigeria	Hypoxaemia prevalence/denominator could not be found

127	The role of body temperature on respiratory rate in children with acute respiratory infections	Hypoxaemia prevalence/denominator could not be found
128	Venoarterial Extracorporeal Membrane Oxygenation for Severe Neonatal Acute Respiratory Distress Syndrome in a Developing Country	Hypoxaemia prevalence/denominator could not be found
129	Community-based amoxicillin treatment for fast breathing pneumonia in young infants 7-59 days old: a cluster randomised trial in rural Bangladesh, Ethiopia, India and Malawi	Hypoxaemia prevalence/denominator could not be found
130	A Self-criticism of Diagnostic and Therapeutic Decision Making in Children Admitted With Acute Lower Respiratory Infection at a Single Pediatric Emergency Department	Hypoxaemia prevalence/denominator could not be found
131	Oxigenoterapia inalatória em pacientes pediátricos internados em hospital universitário	Hypoxaemia prevalence/numerator could not be found
132	[Clinical features and treatment of macrolide-resistant Mycoplasma pneumoniae pneumonia in children]	Hypoxaemia prevalence/ numerator could not be found
133	Ventilação não invasiva na insuficiência respiratória aguda na bronquiolite por vírus sincicial respiratório	Hypoxaemia prevalence/ numerator could not be found
134	Methods of a double-blind randomized controlled clinical trial of three versus five days amoxicillin dispersible tablets for chest-indrawing pneumonia among children 2-59 months of age presenting to kamuzu central hospital in Lilongwe, Malawi	Protocol paper
135	Performance, Acceptability, and Usability of Respiratory Rate Timers and Pulse Oximeters When Used by Frontline Health Workers to Detect Symptoms of Pneumonia in Sub-Saharan Africa and Southeast Asia: Protocol for a Two-Phase, Multisite, Mixed-Methods Trial	Protocol paper
136	Improving oxygen therapy for children and neonates in secondary hospitals in Nigeria: study protocol for a stepped-wedge cluster randomised trial	Protocol paper
137	Children's Oxygen Administration Strategies Trial (COAST): A randomised controlled trial of high flow versus oxygen versus control in African children with severe pneumonia	Protocol paper
138	CPAP Improving Mortality for Pneumonia in African Children Trial	Protocol paper
139	Effectiveness Trial of Day-care vs. Usual Care of Severe Pneumonia & Malnutrition in Children	Protocol paper
140	RCT of Efficacy of Amoxicillin Over Ampicillin on Severe Pneumonia	Protocol paper
141	Impact of Pulse Oximetry on Hospital Referral Acceptance in Children Under 5 With Severe Pneumonia	Protocol paper
142	Immunomodulation by Zinc Supplementation in Children With Pneumonia	Protocol paper
143	Solar-powered oxygen delivery: study protocol for a randomized controlled trial	Protocol paper
144	CPAP IMPACT: a protocol for a randomised trial of bubble continuous positive airway pressure versus standard care for high-risk children with severe pneumonia using adaptive design methods	Protocol paper
145	Automated Respiratory Rate Counter to Assess Children for Symptoms of Pneumonia: Protocol for Cross-Sectional Usability and Acceptability Studies in Ethiopia and Nepal	Protocol paper
146	Solar-powered oxygen delivery for the treatment of children with hypoxemia: protocol for a cluster-randomized stepped-wedge controlled trial in Uganda	Protocol paper
147	Smart triage: Triage and management of sepsis in children using the point-of-care Pediatric Rapid Sepsis Trigger (PRST) tool	Protocol paper
148	Design and conduct of facility-based surveillance for severe childhood pneumonia in the Household Air Pollution Intervention Network (HAPIN) trial	Protocol paper
149	Development of a Robust and Reliable Pulse Oximeter for Children With Pneumonia in Low-income Countries	Published before 2008
150	Use of Bubble Continuous Positive Airway Pressure Compared to Nasal Prong Oxygen or Humidified High Flow in Children Under Five With Severe Pneumonia and Hypoxaemia	Published before 2008
151	Assessing the Feasibility and Effectiveness of Introducing Pulse Oximetry in IMCI Services	Published before 2008
152	CPAP Improving Mortality for Pneumonia in African Children Trial	Published before 2008
153	Infant Pulse Oximetry in Pakistan Study	Published before 2008
154	Vitamin-D Supplementation: Impact on Severe Pneumonia Among Under-five Children	Published before 2008
155	Electronic Algorithms Based on Host Biomarkers to Manage Febrile Children	Published before 2008
156	Impact of Pulse Oximetry on Hospital Referral Acceptance in Children Under 5 With Severe Pneumonia	Published before 2008
157	Multimometer - Vital Signs Device for Diagnosis of Pneumonia	Published before 2008
158	Randomized Controlled Trial (RCT) in Children With Severe Pneumonia	Published before 2008
159	Innovative Treatments in Pneumonia (ITIP) 3	Published before 2008
160	Immunomodulation by Zinc Supplementation in Children With Pneumonia	Published before 2008
161	RCT of Efficacy of Amoxicillin Over Ampicillin on Severe Pneumonia	Published before 2008
162	The Efficacy of Zinc as Adjunct Therapy in the Treatment of Severe Pneumonia in Children	Published before 2008
163	High Flow Oxygen Therapy vs Standard Care in Infants With Viral Bronchiolitis	Published before 2008
164	Vitamin D Supplementation and Respiratory Index of Severity in Children (RISC) in Pneumonia	Published before 2008
165	Nebulized Hypertonic Saline (3%) Versus Nebulized Adrenaline for Treatment of Bronchiolitis	Published before 2008

166	Solar Powered Oxygen Delivery	Published before 2008
167	Advancement of Modified Bubble CPAP	Published before 2008
168	Montelukast for Acute Bronchiolitis and Postbronchiolitis Viral Induced Wheezing	Published before 2008
169	Ceftriaxone Versus Chloramphenicol for Treatment of Severe Pneumonia in Children	Published before 2008
170	A Study to Evaluate a Single IM Dose of Motavizumab Treatment of Children With RSV (Respiratory Syncytial Virus) Illness	Published before 2008
171	Salbutamol versus epinephrine in the treatment of acute bronchiolitis	Published before 2008
172	Oral amoxicillin versus injectable penicillin for severe pneumonia in children aged 3 to 59 months: a randomised multicentre equivalency study	Published before 2008
173	Three day versus five day treatment with amoxicillin for non-severe pneumonia in young children: a multicentre randomised controlled trial	Published before 2008
174	Respiratory syncytial virus and human metapneumovirus in children with acute respiratory infections in Yemen	Published before 2008
175	Epidemiology and clinical characteristics of respiratory syncytial virus infections in Jordan	Published before 2008
176	Oral amoxicillin is as effective as injected penicillin for severe pneumonia in young children	Published before 2008
177	Hypoxemia in children with pneumonia and its clinical predictors	Published before 2008
178	Efficacy of zinc in the treatment of severe pneumonia in hospitalized children <2 y old	Published before 2008
179	Zinc for severe pneumonia in very young children: double-blind placebo-controlled trial	Published before 2008
180	Pneumonia case-finding in the RESPIRE Guatemala indoor air pollution trial: standardizing methods for resource-poor settings	Published before 2008
181	Acquired pneumonia's behavior in a community in the pediatrics service at the Fundacion Cardio Infantil, Bogota, Colombia. [Spanish]	Published before 2008
182	Risk factors for hypoxemia and respiratory failure in respiratory syncytial virus bronchiolitis	Published before 2008
183	Case fatality proportions and predictive factors for mortality among children hospitalized with severe pneumonia in a rural developing country setting	Published before 2008
184	Treatment of respiratory syncytial virus infection with vitamin A: A randomized, placebo-controlled trial in Santiago, Chile	Published before 2008
185	Hypoxaemia in acute respiratory and non-respiratory illnesses in neonates and children in a developing country	Published before 2008
186	Hypoxaemia in children with severe pneumonia in Papua New Guinea	Published before 2008
187	Chloramphenicol versus benzylpenicillin and gentamicin for the treatment of severe pneumonia in children in Papua New Guinea: A randomised trial	Published before 2008
188	Predicting hypoxia in children with acute lower respiratory infection: A study in the highlands of Papua New Guinea	Published before 2008
189	PREDICTING HYPOXIA IN CHILDREN WITH ACUTE LOWER RESPIRATORY-INFECTION - A STUDY IN THE HIGHLANDS OF PAPUA-NEW-GUINEA	Published before 2008
190	Hypothetical performance of syndrome-based management of acute paediatric admissions of children aged more than 60 days in a Kenyan district hospital	Published before 2008
191	Causes and outcome of young infant admissions to a Kenyan district hospital	Published before 2008
192	Clinical overlap between malaria and severe pneumonia in African children in hospital	Published before 2008
193	Clinical indicators of Pneumocystis jiroveci pneumonia (PCP) in South African children infected with the human immunodeficiency virus	Published before 2008
194	Vitamin A supplementation and severity of pneumonia in children admitted to the hospital in Dar es Salaam, Tanzania	Published before 2008
195	Brief hospitalization and pulse oximetry for predicting amoxicillin treatment failure in children with severe pneumonia	Published before 2008
196	ORAL VERSUS NEBULIZED ALBUTEROL IN THE MANAGEMENT OF BRONCHIOLITIS IN EGYPT	Published before 2008
197	Caretaker recognition of respiratory signs in children: Correlation with physical examination findings, X-ray diagnosis and pulse oximetry	Published before 2008
198	Clinical presentation and outcome of Pneumocystis carinii pneumonia in Malawian children	Published before 2008
199	Relación entre signos clínicos e hipoxemia en niños menores de 5 años con enfermedad respiratoria aguda baja	Published before 2008
200	A study of some viral pathogens in infants and children with lowe respiratory tract infection and attending the intensive care unit	Published before 2008
201	MATERNAL REPORTING OF ACUTE RESPIRATORY-INFECTION IN EGYPT	Published before 2008
202	The spectrum of hypoxaemia in children admitted to hospital in The Gambia, West Africa	Published before 2008
203	Evaluation of clinical and radiological signs in diagnosis of pneumonia in the first five years of life	Published before 2008
204	Remark on the changes of blood gas in newborn babies with respiratory impairment due to pneumonia in the Department of Neonate at the National Institute of Pediatry -2001 - 2002 period	Published before 2008
205	High rates of Chlamydia trachomatis infections in young Papua New Guinean infants	Published before 2008

206	Can Clinical Symptoms or Signs Accurately Predict Hypoxemia in Children with Acute Lower Respiratory Tract Infections?	Published before 2008
207	Clinical predictors of acute radiological pneumonia and hypoxaemia at high altitude	Published before 2008
208	Central nervous system infection is an important cause of death in underfives hospitalised with World Health Organization (WHO) defined severe and very severe pneumonia	Published before 2008
209	The role of pulse oximetry: Its use as an indicator of severe respiratory disease in Peruvian children living at sea level	Published before 2008
210	Equivalency Of Oral Amoxicillin Vs Injectable Penicillin In Children With Severe Pneumonia	Published before 2008
211	Hypoxaemia in young Kenyan children with acute lower respiratory infection	Published before 2008
212	Analysis of Acid-Base Disturbance Caused by Severe Pneumonia in Newbern Infants	Published before 2008
213	Prevalence and prediction of hypoxemia in children with respiratory infections in the Peruvian Andes	Published before 2008
214	Respiratory Syncytial Virus (RSV) in infants hospitalized for acute lower respiratory tract disease: Incidence and associated risks	Published before 2008
215	Características clínicas e da saturação transcutânea de oxigênio em lactentes hospitalizados com bronquiolite viral aguda	Published before 2008
216	Clinical predictors of hypoxaemia in children with pneumonia	Published before 2008
217	Efficacy of dexamethasone injection for acute bronchiolitis in hospitalized children: A randomized, double-blind, placebo-controlled trial	Published before 2008
218	Effectiveness of intramuscular penicillin versus oral amoxicillin in the early treatment of outpatient pediatric pneumonia	Published before 2008
219	Clinical predictors of hypoxaemia in Gambian children with acute lower respiratory tract infection: Prospective cohort study	Published before 2008
220	Hypoxaemia among children in rural hospitals in Papua New Guinea: Epidemiology and resource availability - A study to support a national oxygen programme	Published before 2008
221	Clinical Analysis of Complications of 115 Children with Serious Pneumonia with Ventilatory Support	Published before 2008
222	The clinical spectrum of respiratory syncytial virus disease in The Gambia	Published before 2008
223	An epidemiological study of RSV infection in the Gambia	Published before 2008
224	Long-term morbidity and mortality following hypoxaemic lower respiratory tract infection in Gambian children	Published before 2008
225	Observational follow-up study following two cohorts of children with severe pneumonia after discharge from day care clinic/hospital in Dhaka, Bangladesh	Same study reported prevalence
226	Co-morbidity: Exploring the clinical overlap between pneumonia and diarrhoea in a hospital in Dhaka, Bangladesh	Same study reported prevalence
227	Risk factors for mortality in childhood pneumonia in a rural West African region	Same study reported prevalence
228	Predictors of prolonged hospitalisation in childhood pneumonia in a rural health centre	Same study reported prevalence
229	Safety, Effectiveness and Feasibility of Outpatient Management of Children with Pneumonia with Chest Indrawing at Port Moresby General Hospital, Papua New Guinea	Same study reported prevalence
230	Impaired Gas Exchange: Prognostic Clinical Indicators of Short-Term Survival in Children with Acute Respiratory Infection	Same study reported prevalence

Supplementary Figure 1: Hypoxaemia prevalence among non-WHO-classified pneumonia * (presented in %)

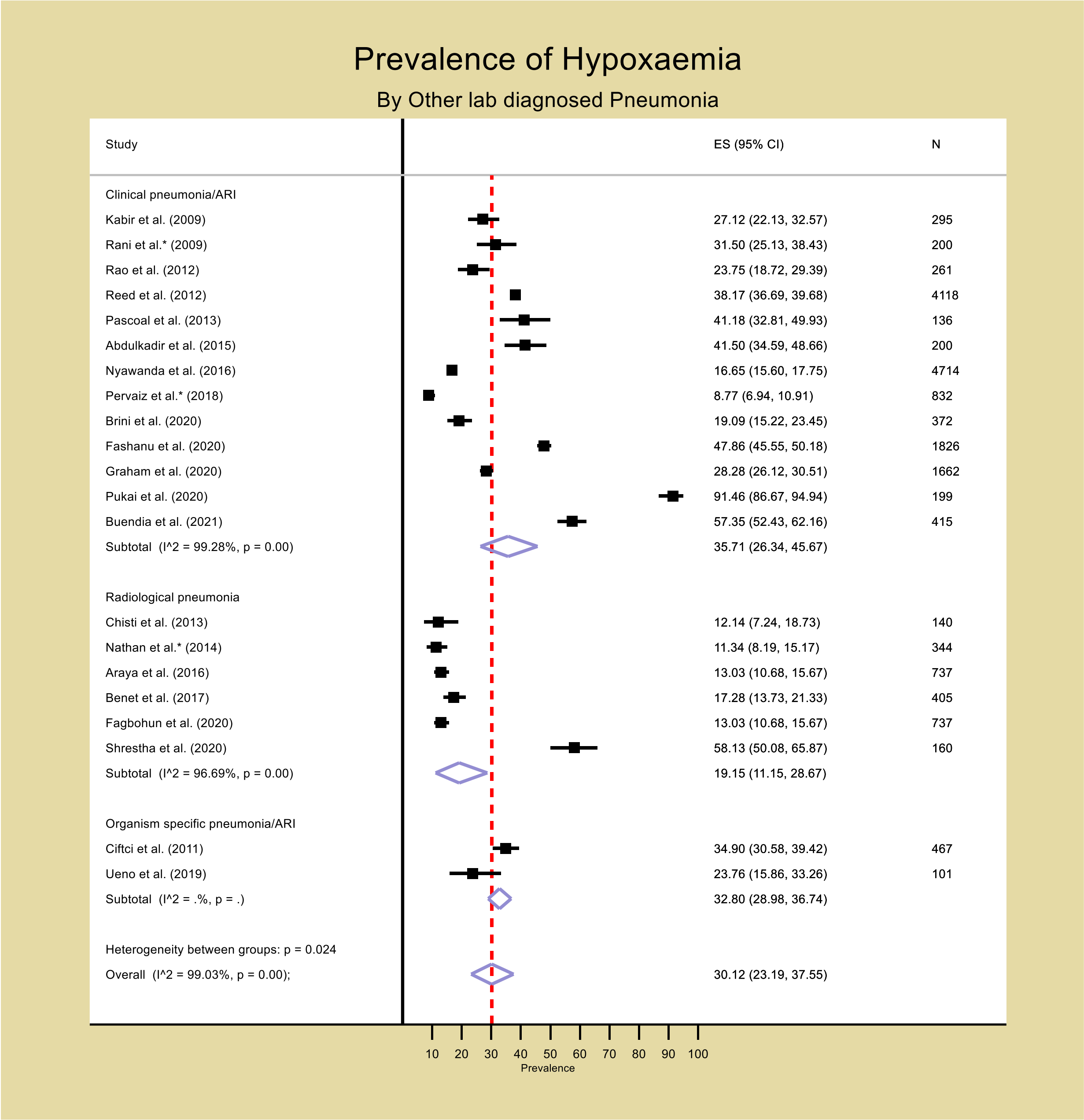

The broken red line shows the overall pooled prevalence of hypoxaemia.
*Studies where SpO2 cut-off is >90%

Supplementary figure 2a: Funnel plot to assess bias in the studies which reported hypoxaemia among non-severe cases

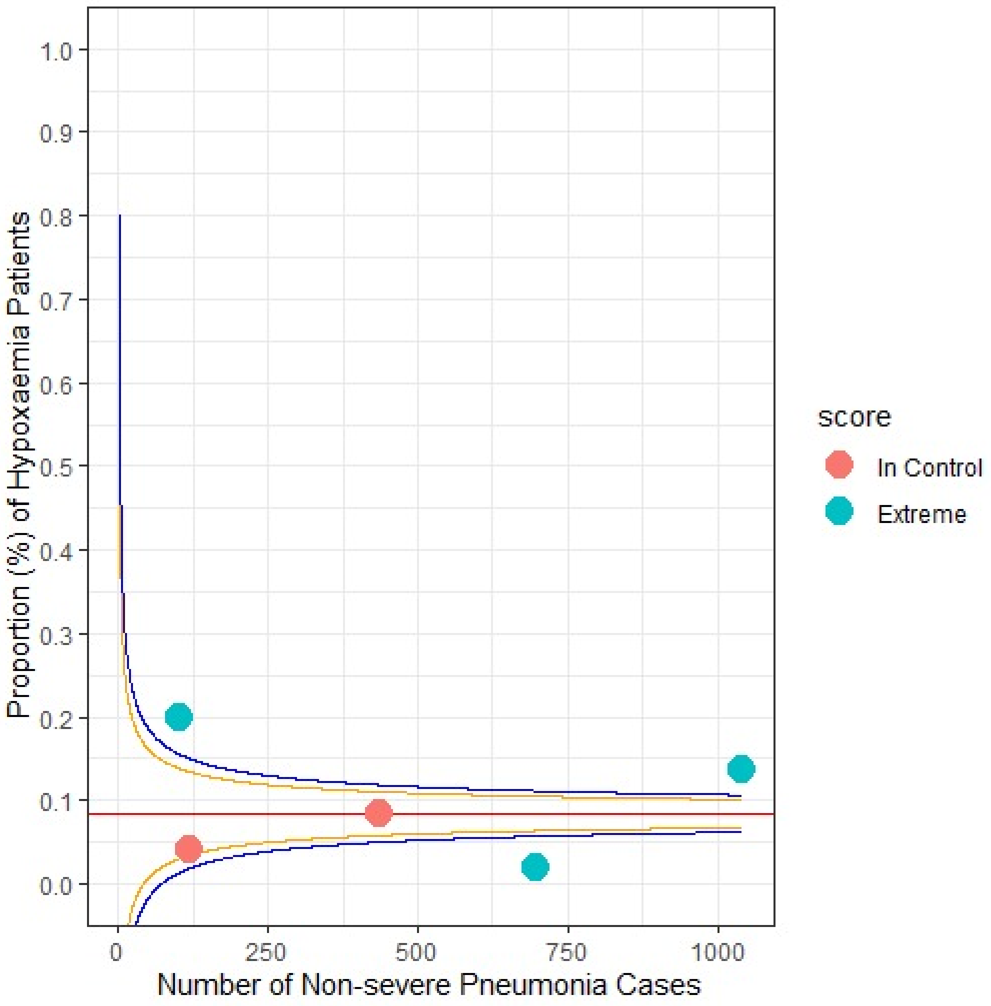

Supplementary figure 2b: Funnel plot to assess bias in the studies which reported hypoxaemia among severe or very severe cases

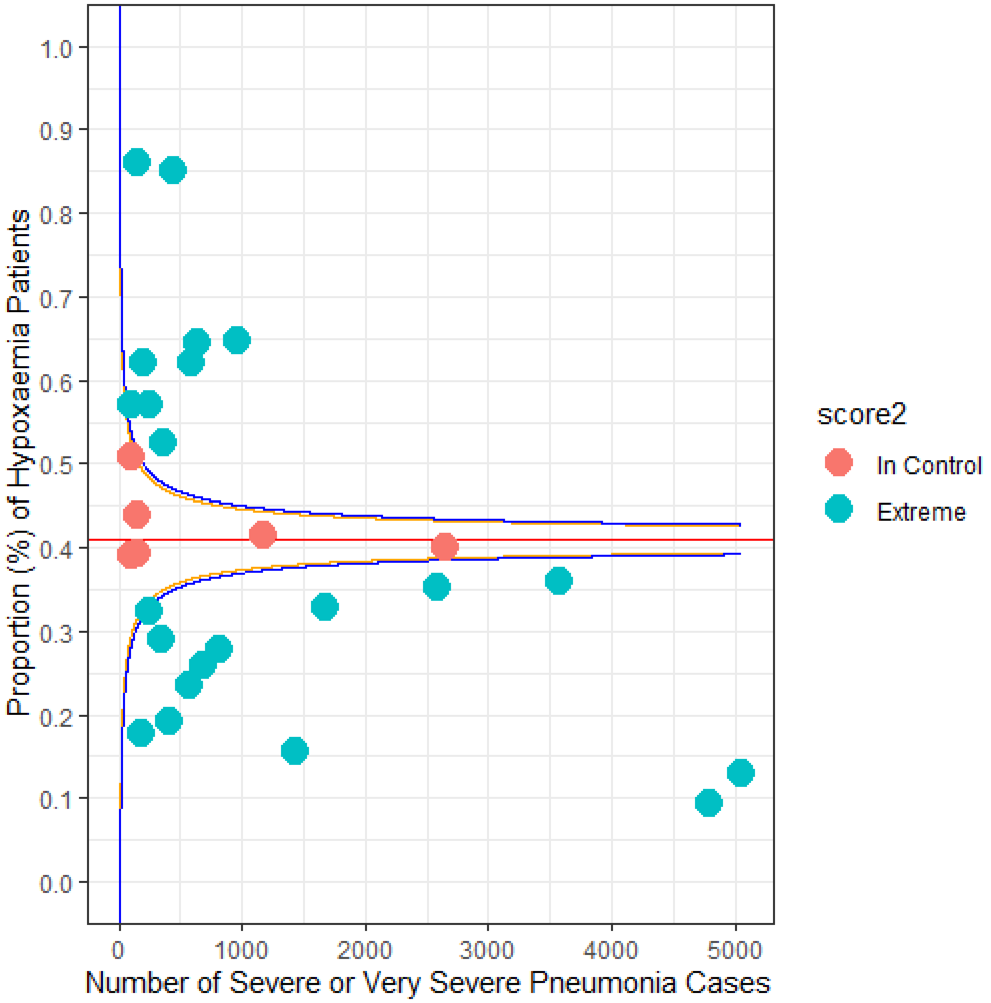

Supplementary figure 2c: Funnel plot to assess bias in the studies which reported hypoxaemia among unclassified cases

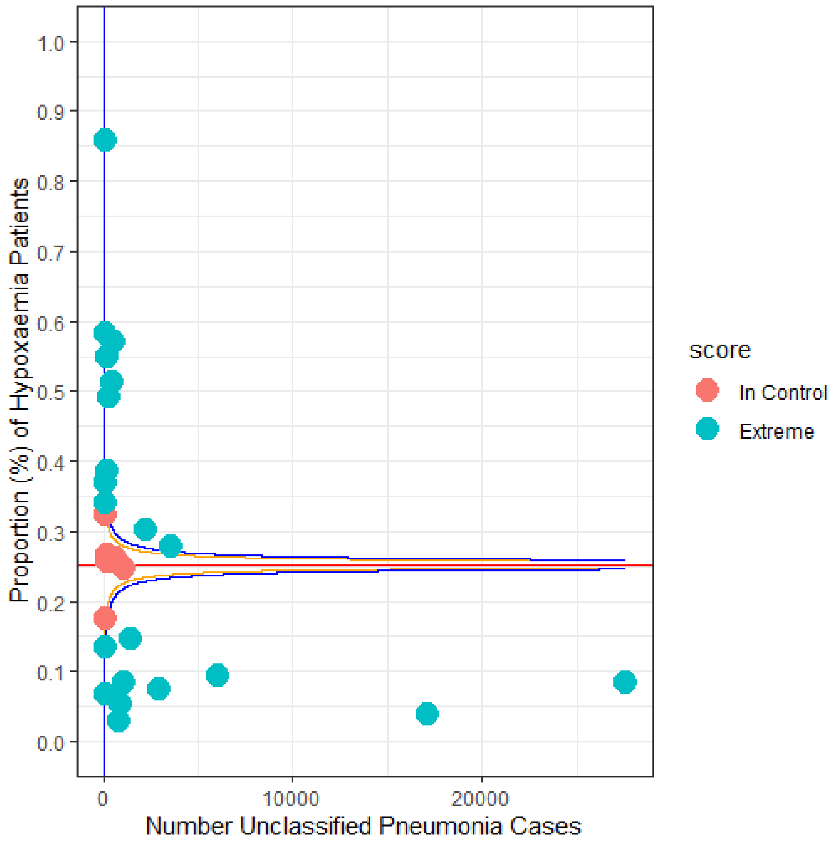

Supplementary Figure 3: Hypoxaemia prevalence (presented in %) among children with WHO-classified pneumonia by type of patients

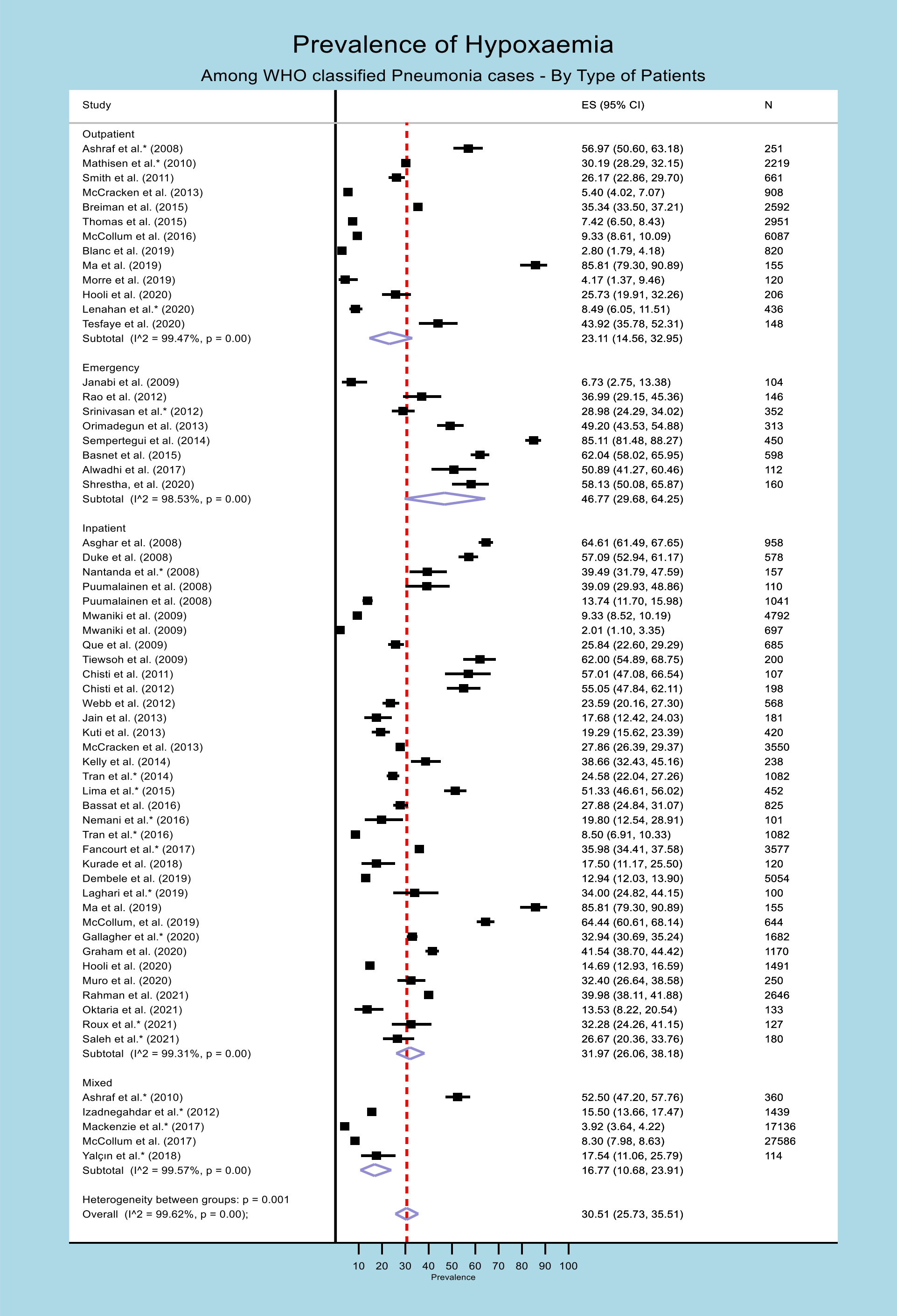

The broken red line shows the overall pooled prevalence of hypoxaemia.
*Studies where SpO2 cut-off is >90%

Supplementary Figure 4: Hypoxaemia prevalence (presented in %) among WHO-classified pneumonia by region*

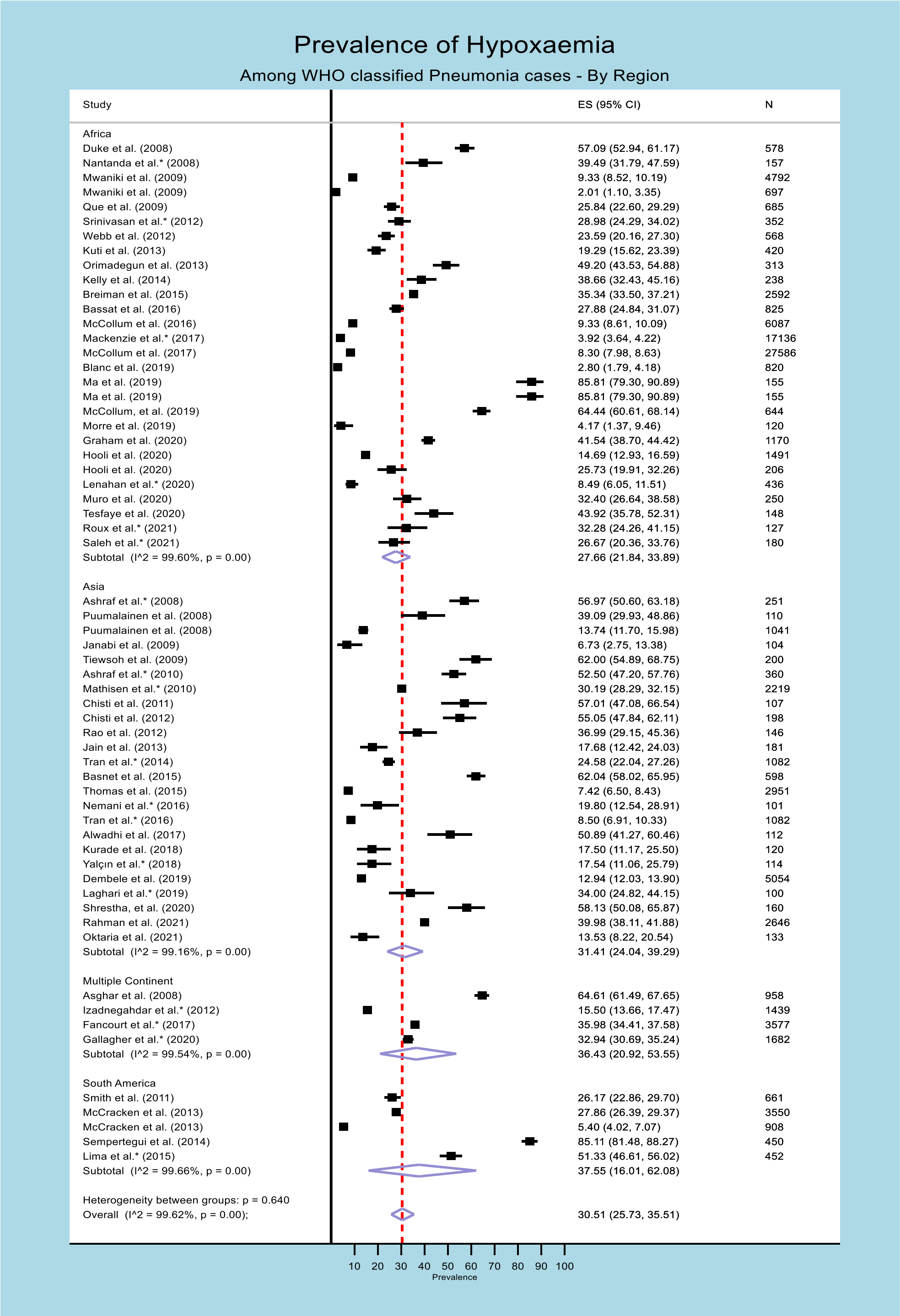

The broken red line shows the overall pooled estimate of prevalence of hypoxaemia.
*Studies where SpO2 cut-off is >90%

Supplementary Figure 5: Hypoxaemia prevalence (presented in %) among WHO-classified pneumonia by publication year*

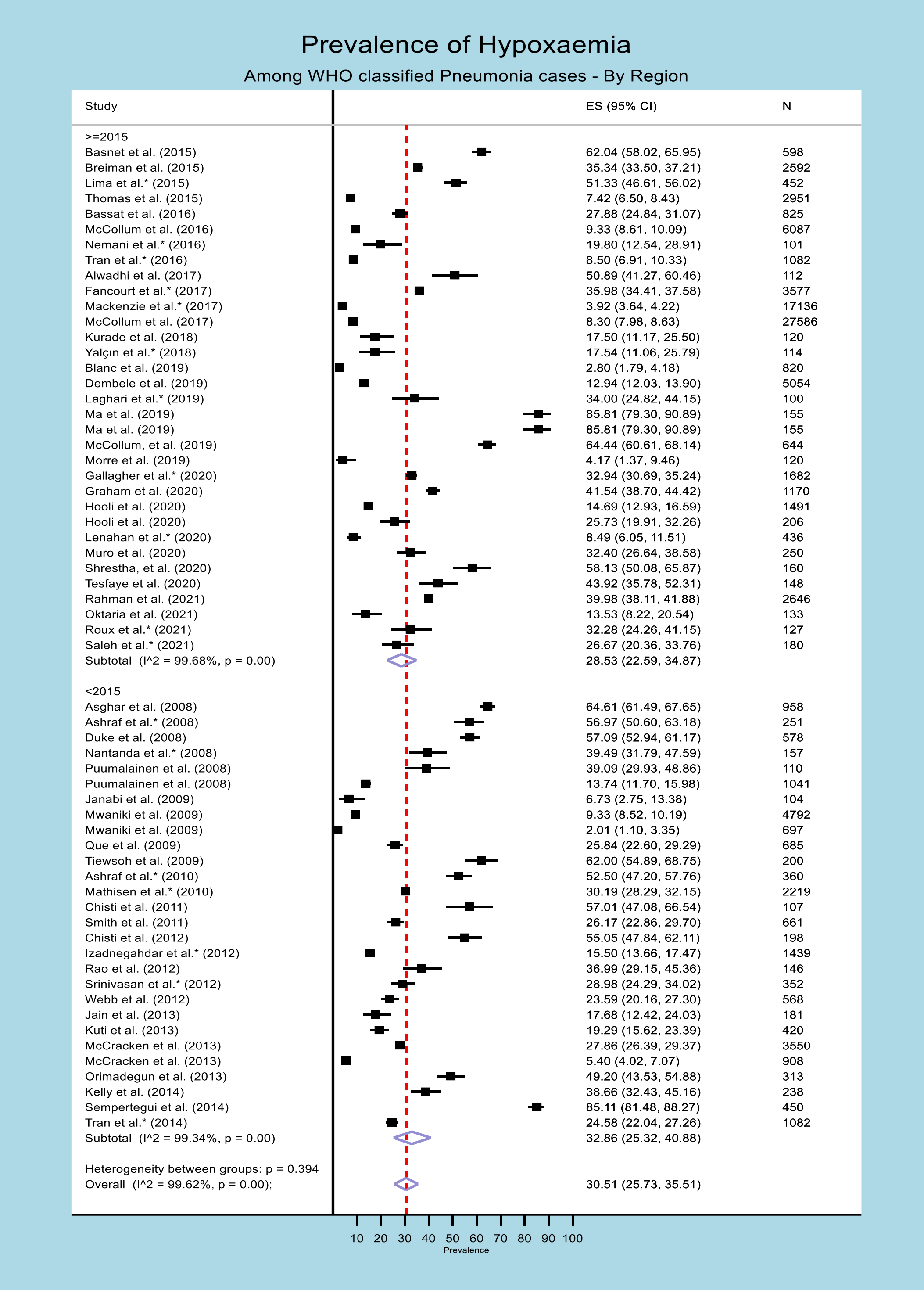

*Studies where SpO2 cut-off is >90%

Supplementary Figure 6: Hypoxaemia prevalence among WHO -classified pneumonia by Facility Type*

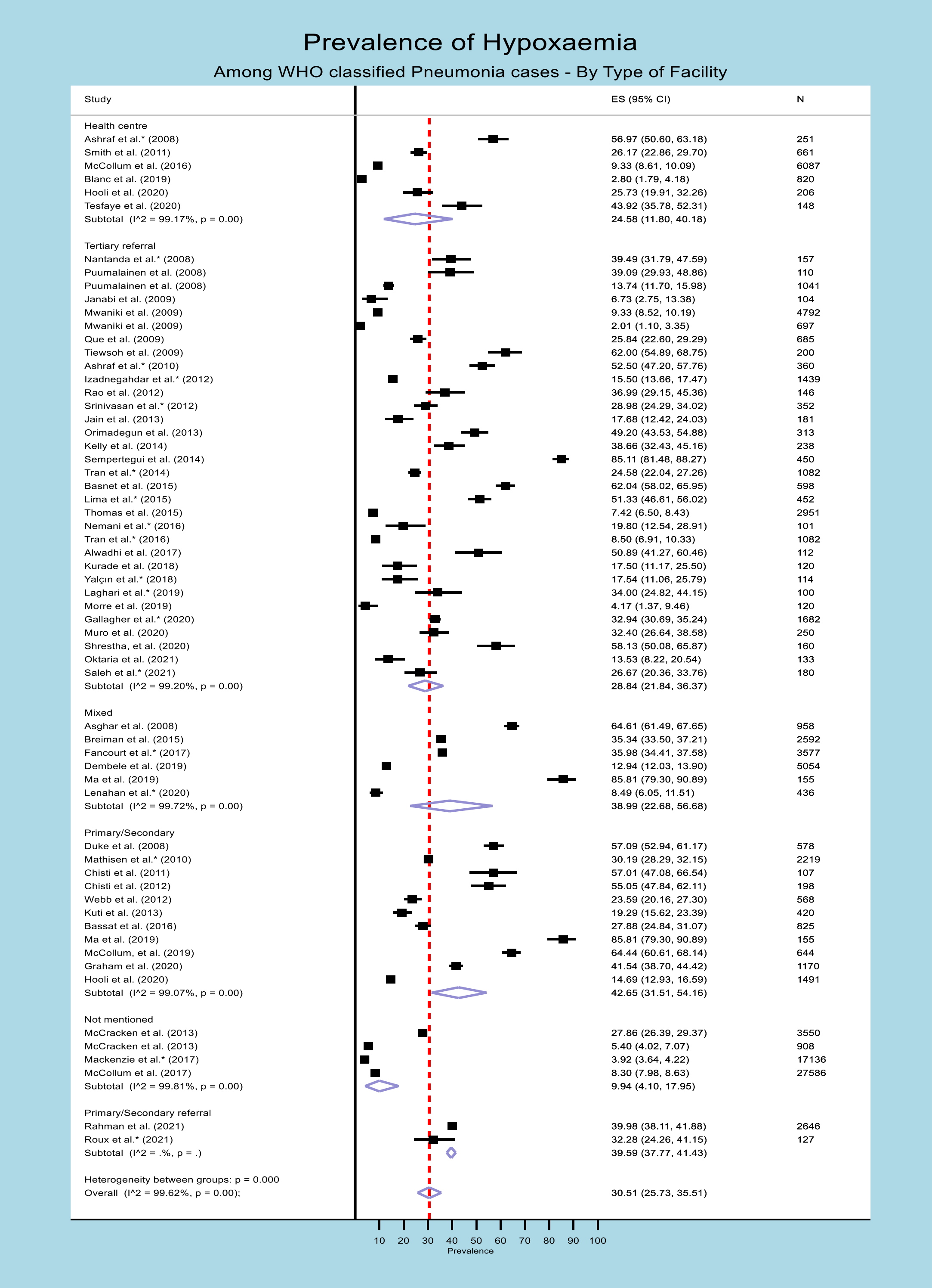

*Studies where SpO2 cut-off is >90%

Supplementary Figure 7: Hypoxaemia prevalence among WHO-classified pneumonia by Patient Status*

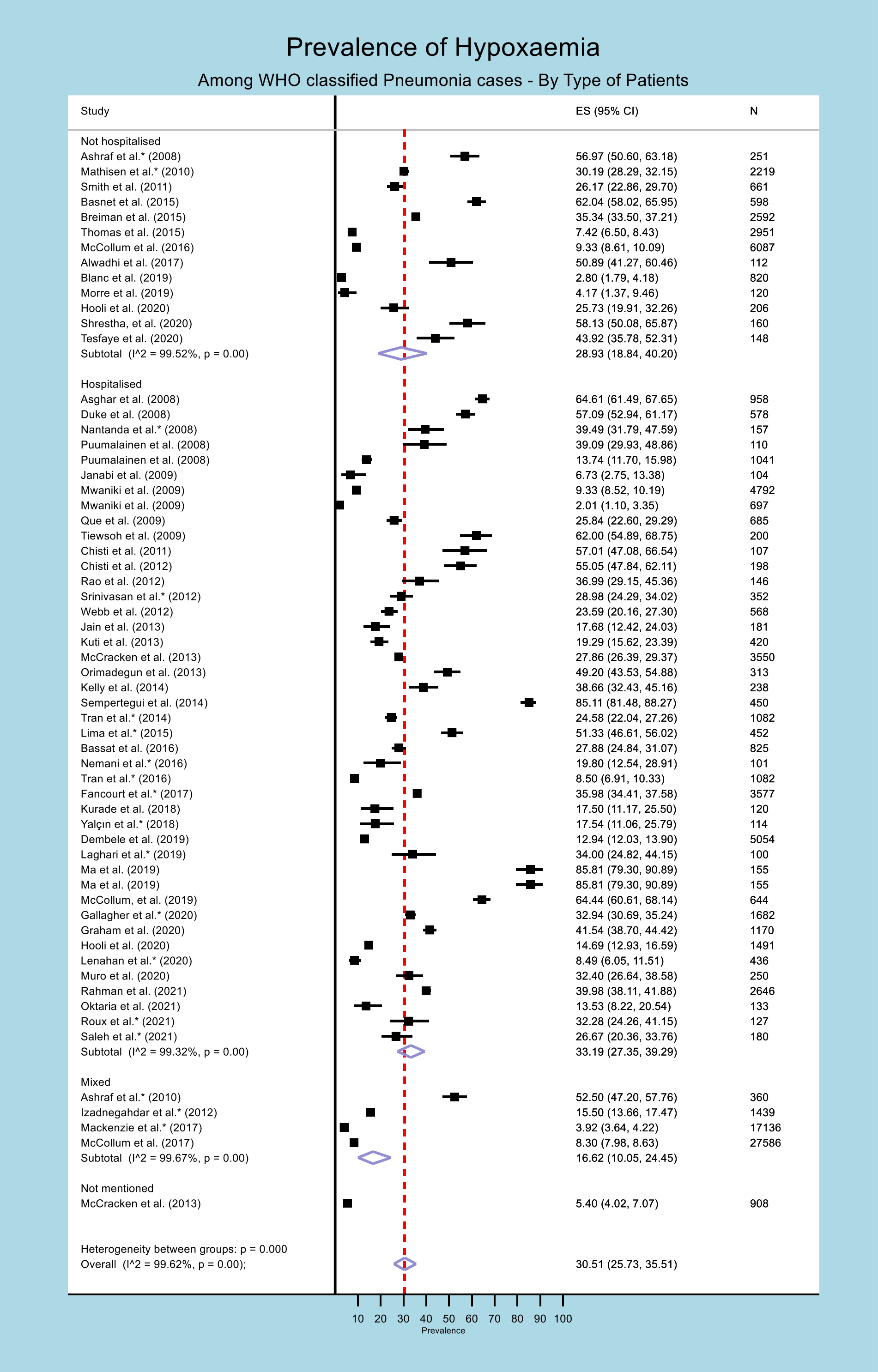

The broken red line shows the overall pooled prevalence of hypoxaemia.
*Studies where SpO2 cut-off is >90%

Supplementary Material-1

Key revisions in the IMCI guideline by WHO from versions 2008 to 2014

The previous IMCI guidelines (published in 2008) recommend that any child presenting with cough or difficult breathing should be assessed clinically for the presence of any danger sign, chest in-drawing, stridor, wheezing; and would be classified as one of the followings (**Error! Reference source not found.**):1. 'Severe Pneumonia/Very Severe Disease' ; 2. 'Pneumonia', 3. 'Cough or Cold'

The sensitivity of the previous definition of 'Severe Pneumonia/Very Severe Disease' was very high but it had issues with low specificity. This resulted in over referral of 'Severe Pneumonia/Very Severe Disease' cases to the hospital for inpatient care. As a results, it was imperative to reassess the validity of 'severe pneumonia' classification as outlined in the previous IMCI guidelines (2008). Prognostic value and treatment options for different signs of 'severe pneumonia' including chest in-drawing required a critical evaluation.

The new IMCI guidelines (2014) recommends that any child (2 months to 59 months) presenting with fast breathing or chest in-drawing (and no other danger signs) should be classified as 'Pneumonia', and would be treated through home-based management with oral antibiotics (amoxicillin for five days). In addition to other changes in the management to malnutrition, this was the most significant shift from the previous classification and treatment recommendation for pneumonia management. Previously, any child with chest indrawing would be classified as 'Severe Pneumonia', therefore would have been urgently referred to the hospital.

An illustrative comparison of the 2008 and 2014 version of IMCI guidelines is presented in Figure 1.

Figure 1: Difference in classification and treatment of children age 2-59 months presenting with cough or difficult breathing according to the IMCI guidelines (2008 vs. 2014) (adopted from the WHO evidence summary)

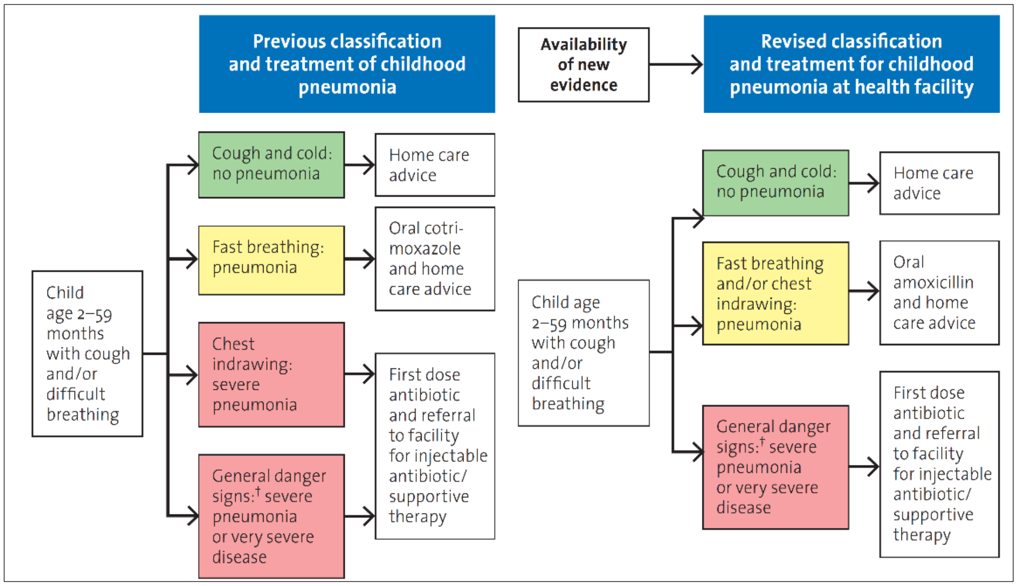

† Not able to drink, persistent vomiting, convulsions, lethargic or unconscious, stridor in a calm child or severe malnutrition.
